# Enzymatic Synthesis and Structural Characterization of Novel Trehalose-Based Oligosaccharides

**DOI:** 10.1021/acs.jafc.1c03768

**Published:** 2021-10-12

**Authors:** Pablo Gallego-Lobillo, Elisa G. Doyagüez, María Luisa Jimeno, Mar Villamiel, Oswaldo Hernandez-Hernandez

**Affiliations:** † Institute of Food Science Research (CIAL), Spanish Council of Scientific Research, (CSIC)−Autonomous University of Madrid (UAM), Campus de la Universidad Autónoma de Madrid, c/Nicolás Cabrera, 9, Madrid E-28049, Spain; ‡ Centro de Química Orgánica “Lora Tamayo” (CSIC), c/Juan de la Cierva, 3, Madrid E-28006, Spain

**Keywords:** trehalose, oligosaccharide, β-galactosidase, prebiotic, transgalactosylation, GOS

## Abstract

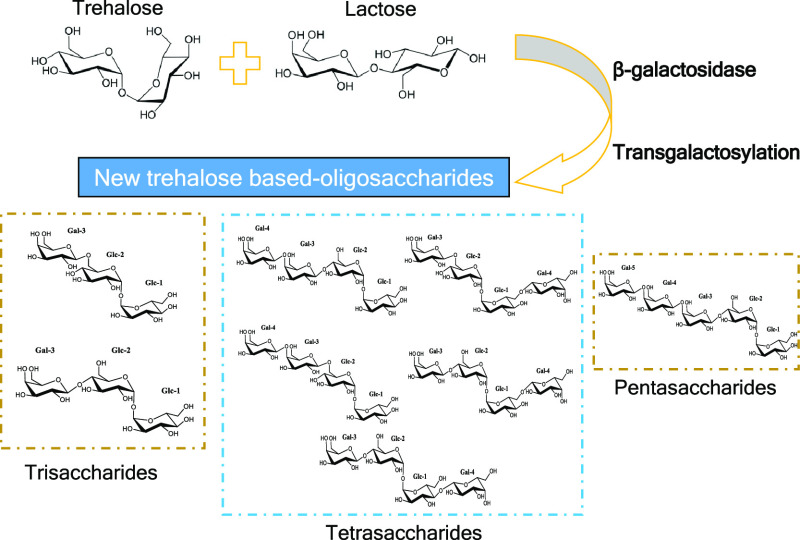

Trehalose, α-d-glucopyranosyl-(1↔1)-α-d-glucopyranoside, is a disaccharide with multiple effects on the human body. Synthesis of new trehalose derivatives was investigated through transgalactosylation reactions using β-galactosidase from four different species. β-galactosidases from *Bacillus circulans* (*B. circulans*) and *Aspergillus oryzae* (*A. oryzae*) were observed to be the best biocatalysts, using lactose as the donor and trehalose as the acceptor. Galactosyl derivatives of trehalose were characterized using nuclear magnetic resonance spectroscopy. Trisaccharides were the most abundant oligosaccharides obtained followed by the tetrasaccharide fraction (19.5% *vs* 8.2% carbohydrates). Interestingly, the pentasaccharide [β-Gal*p*-(1→4)]^3^-trehalose was characterized for the first time. Greater oligosaccharide production was observed using β-galactosidase from *B. circulans* than that obtained from *A. oryzae*, where the main structures were based on galactose monomers linked by β-(1→6) and β-(1→4) bonds with trehalose in the ending. These results indicate the feasibility of commercially available β-galactosidases for the synthesis of trehalose-derived oligosaccharides, which might have functional properties, excluding the adverse effects of the single trehalose.

## 1 Introduction

Trehalose, α-d-glucopyranosyl-(1↔1)-α-d-glucopyranoside, is a nonreducing disaccharide with a wide range of applications as a food additive as well as in cosmetics and pharmaceutical sciences.^1,2^ Trehalose can be found in multiple sources such as invertebrates, fungi, and bacteria, holding a protective function against dryness and stress.^1,3^ This disaccharide was generally recognized as safe (GRAS) in 2000 by the Food & Drug Administration (FDA, USA), with an equivalent sweetness of 45% sucrose and being mainly used in the food industry as a sweetener, stabilizer, and moisturizer.^4^ In this context, it is important to emphasize its high stability in terms of temperature and pH. In addition, its α-bond is not hydrolyzed by the pancreatic α-amylase, and it has considerable resistance to acids.^5,6^

Multiple functionalities of trehalose such as a decrease in the postprandial glucose level,^7,8^ improvement of the insulin resistance,^8,9^ prevention of adipocyte hypertrophy,^9,10^ suppression of bone resorption,^11^ and induction of autophagy^12^ have been reported. Regarding the latter, it is of paramount importance in the case of neurodegenerative and metabolic diseases. Autophagy is a conserved degradation mechanism of the cell, which can be regulated by trehalose, due to the chemical chaperone properties.^6,13^ This fact opens a new field in the development of some rare diseases, such as Huntington’s and Parkinson’s diseases.^14−16^ Furthermore, the latest studies have found promising results of trehalose in amyotrophic lateral sclerosis (ALS).^17^ All these beneficial properties occur in response to its absorption in the small intestine and then the transport to the target organs through the bloodstream.^6,14^

Enzymatic complexes of disaccharidases are placed in the brush border membranes of the enterocytes, including the enzyme trehalase, which hydrolyzes the trehalose into two molecules of glucose.^18^ The content of trehalase in the small intestine is lower than other small-intestinal disaccharidases,^19^ and a considerable amount of the disaccharide (∼75 g) is necessary to observe a real benefit in the organism.^20,21^ Then, a substantial quantity of the saccharide might not be hydrolyzed or absorbed in the small intestine, and therefore, it would reach the colon. This fact could become a drawback, especially due to the feeding of some species of pathogenic microorganisms, which can be present in the gut, such as *Clostridium difficile* (*C. difficile*).

Recent findings have shown the development of genetic mechanisms by *C. difficile* species to metabolize trehalose.^22,23^ It has been observed that these bacteria could increase significantly the risk of death when they were fed with trehalose, causing strong diarrhea.^24^ Trehalose does not induce bacterial growth; nevertheless, it seems to increase the production of toxins that could exacerbate the infection.^22^ Synthesis of trehalose derivatives might be a solution to reduce the adverse effects of this carbohydrate. These derivatives would suppose a change in the chemical structure of the classic trehalose; therefore, their behavior in the human body would be different. In addition, these new compounds might have new beneficial properties.

Concerning the synthesis processes of new oligosaccharides, enzymatic procedures, such as the use of β-galactosidases (β-gal), are proven to be reliable, cost-effective, and environmentally friendly. β-Gal of *Escherichia coli* (*E. coli*) were used to produce trisaccharides from trehalose through transgalactosylation, obtaining structures such as β-Gal*p*-(1→4)-trehalose and β-Gal*p*-(1→6)-trehalose.^25^ In previous works, β-gal activities from different microorganisms have shown an excellent ability to form lactose derivatives such as galactooligosaccharides (β-GOS);^26−29^ however, to the best of our knowledge, few studies with trehalose as an acceptor have been carried out so far. A straightforward process of synthesis may permit the obtainment of new bioactive molecules from trehalose, avoiding the negative effects by *C. difficile*. Therefore, the aim of this work was to study the usefulness of four commercial β-gal to synthesize new oligosaccharides derived from trehalose, through transgalactosylation reactions. Different sources of the enzymes have been tested: *Bacillus circulans* (*B. circulans*), *Aspergillus oryzae* (*A. oryzae*)*,Bifidobacterium bifidum* (*B. bifidum*), and *Kluyveromyces lactis* (*K. lactis*). Syntheses were studied with lactose (as a galactosyl donor) and trehalose (as an acceptor). Trehalose derivatives were characterized by nuclear magnetic resonance spectroscopy (NMR spectroscopy).

## 2 Materials and Methods

### 2.1 Chemicals and Standards

Standards of d-galactose (Gal), d-glucose (Glc), trehalose, lactose, raffinose, nystose, phenyl-β-d-glucoside, and activated charcoal were purchased from Sigma-Aldrich (St. Louis, MO, USA). Lactose was supplied by ACROS Organics (Geel, Belgium). HPLC-grade acetonitrile and ethanol (96%) were obtained from VWR (Barcelona, Spain). All reagents were of analytical grade (purity of >95%). Standard GOS (3′-galactosyllactose, 4′-galactosyllactose, 6′-galactosyllactose, 3′-galactobiose, 4′-galactobiose, and 6′-galactobiose) were purchased from Biosynth Carbosynth (Reading, UK).

### 2.2 Commercial Enzyme Preparations

Four commercial β-gal preparations were evaluated in this work: Lactozym Pure 6500 L [*Kluyveromyces lactis* (6500 U g^–1^)] and Saphera 2600 L [*Bifidobacterium bifidum* (2600 U g^–1^)] were kindly provided by Novozymes A/S (Bagsvaerd, Denmark), biolactase [*Bacillus circulans* (1500 U g^–1^)] was supplied by Biocon (Barcelona, Spain), and β-gal from *Aspergillus oryzae* (111,000 U g^–1^) was provided by Sigma-Aldrich (St. Louis, MO, USA).

### 2.3 Enzymatic Synthesis of Trehalose Derivatives

Enzymatic synthesis with *K. lactis* and *B. bifidum* β-gal was performed in 50 mM sodium phosphate buffer with 2 mM MgCl_2_ at pH 6.5. *A. oryzae* and *B. circulans* β-gal assays were carried out in 50 mM sodium acetate buffer at pH 4.5. The enzymatic activity used for the four enzymes was 15 U mL^–1^.

Transgalactosylation reactions were carried out in an orbital Thermomixer comfort (Eppendorf) at 50 °C and 750 rpm using trehalose (25% w/v), as an acceptor, and lactose (25% w/v), as a donor. Reactions were incubated during 24 h, taking aliquots at 0, 2, 4, 6, and 24 h. Assays were stopped by heating in boiling water for 5 min. In all cases, reaction blanks using only lactose were performed under the same conditions. Transgalactosylation reactions were monitored through gas chromatography coupled to a flame ionization detector (GC-FID), as described below. After GC-FID analysis, β-gal from *K. lactis* and *B. bifidum* did not show any formation of new trehalose derivatives. For this reason, analysis, isolation, and characterization were carried out using *A. oryzae* and *B. circulans* β-gal*.*

### 2.4 Analysis of the Carbohydrate Content by Gas Chromatography (GC-FID)

Samples were derivatized to their corresponding trimethyl silylated oximes (TMSOs) according to Brobst and Lott.^30^ Analysis was performed in an Agilent Technologies 7820A gas chromatograph system. Separation of the samples was carried out in a fused silica capillary column DB-5HT (5% phenyl methylpolysiloxane, 30 m × 0.25 mm × 0.1 μm) (Agilent J&W Scientific, Folsom, CA, USA). The oven temperature started at 150 °C and then increased to 380 °C at a rate of 3 °C min^–1^. Injector and detector temperatures were 280 and 385 °C, respectively. Samples were analyzed in split mode 1:20 using nitrogen as the carrier gas at a 1 mL min^–1^ flow rate.

Data quantification was calculated through the internal standard method (phenyl-β-d-glucopyranoside, 0.5 mg mL^–1^) and the corresponding response factors of standard solutions of carbohydrates (galactose, glucose, trehalose, lactose, raffinose, and nystose) at known concentrations (0.005–1 mg mL^–1^). All analyses were carried out in triplicate.

### 2.5 Purification and Isolation of Trehalose Derivatives

#### 2.5.1 Activated Charcoal Treatment

With the objective to remove monosaccharides and concentrate the oligosaccharide fraction, synthesized samples after 6 (*A. oryzae*) and 24 h (*B. circulans*) were purified using activated charcoal according to Hernández et al*.*^31^ First, 10 mL of the mixture of the reaction was mixed with 60 g of activated charcoal and 1 L of ethanol solution (5% v/v). This solution was incubated for 30 min at 25 °C under continuous agitation. Then, it was filtered through Whatman No.1 paper. The desorption of oligosaccharides was carried out by mixing the activated charcoal with ethanolic solution (50% v/v) during 30 min and filtering, and then, the ethanolic solution was evaporated for subsequent isolation.

#### 2.5.2 Isolation by Semi-Preparative Chromatography

Trehalose-derived oligosaccharides were isolated by hydrophilic interaction liquid chromatography equipped with a refractive index detector (HILIC-RID), following the method of Julio-González et al.^32^ Sample separation was carried out by a semi-preparative ZORBAX NH2 column (PrepHT cartridge 250 × 21.2 mm, 7 μm particle size) (Agilent), using acetonitrile/water (70:30, v/v) as a mobile phase, at a flow rate of 21 mL min^–1^ for 45 min at 25 °C. Two milliliters was repeatedly injected. The main synthesized compounds were collected, evaporated, and freeze-dried for characterization by NMR and GC–MS.

### 2.6 Structural Characterization of Trehalose Derivatives

#### 2.6.1 Gas Chromatography–Mass Spectrometry (GC–MS)

Trehalose-derived oligosaccharides were also studied by GC–MS. Analysis was performed by a fused silica capillary column DB-5HT (5% phenyl methylpolysiloxane; 30 m × 0.25 mm × 0.10 μm) (Agilent) in a 6890 gas chromatograph system coupled to a 5973 quadrupole mass detector (Agilent). Helium was used as the carrier gas at a flow of 0.8 mL min^–1^. The oven temperature program was 150 °C, increased to 300 °C at 3 °C min^–1^, and maintained for 10 min. Injections were done in split mode (1:20). Electron impact (EI) mode was used at 70 eV in the mass spectrometer, considering a range of 35–700 *m*/*z*. Interface and source temperatures were 280 and 230 °C, respectively.

Identification of known structures of GOS was done by comparison of standard solutions and their respective mass spectra.

#### 2.6.2 Nuclear Magnetic Resonance Spectroscopy (NMR Spectroscopy)

Structure elucidation of the purified fractions was accomplished by nuclear magnetic resonance spectroscopy (NMR spectroscopy). NMR spectra were recorded at 298 K, using D_2_O as a solvent, on an Agilent system 500 NMR spectrometer (^1^H 500 MHz, ^13^C 125 MHz) equipped with a 5 mm HCN cold probe. Chemical shifts of ^1^H (δ_H_) and ^13^C (δ_C_) in parts per million were determined relative to internal standards of sodium [2,2,3,3-^2^H_4_]-3-(trimethylsilyl)-propanoate in D_2_O (δ_H_ 0.00) and 1,4-dioxane (δ_C_ 67.40) in D_2_O, respectively. One-dimensional (1D) NMR experiments (^1^H and ^13^C{^1^H}) were performed using standard pulse sequences. Two-dimensional (2D) [^1^H, ^1^H] NMR experiments [gradient correlation spectroscopy (gCOSY), total correlation spectroscopy (TOCSY), and rotating-frame Overhauser effect spectroscopy (ROESY)] were carried out with the following parameters: a delay time of 1 s, a spectral width of 2800 Hz in both dimensions, 2048 complex points in t2, 4 transients (24 or 32 for ROESY) for each of 128 (200 for TOCSY and ROESY) time increments, and linear prediction to 512. The mixing time used for ROESY was 0.3 ms. The data were zero-filled to 2048 × 2048 real points. 2D [^1^H–^13^C] NMR experiments [gradient heteronuclear single-quantum coherence (gHSQC), hybrid experiment gHSQC-TOCSY, and selective 2D bsgHMBC^200^ used the same ^1^H spectral window, a ^13^C spectral window of 7541.5 Hz, 1 s relaxation delay, 1024 data points, and 128 or 200 time increments, with a linear prediction to 256. The data were zero-filled to 2048 × 2048 real points. Typical numbers of transients per increment were 4 and 16. A mixing time of 80 ms was used for gHSQC-TOCSY experiments.

## 3 Results and Discussion

### 3.1 Transgalactosylation of Trehalose by β-Galactosidase from *Bacillus circulans*

**Figure 1:**
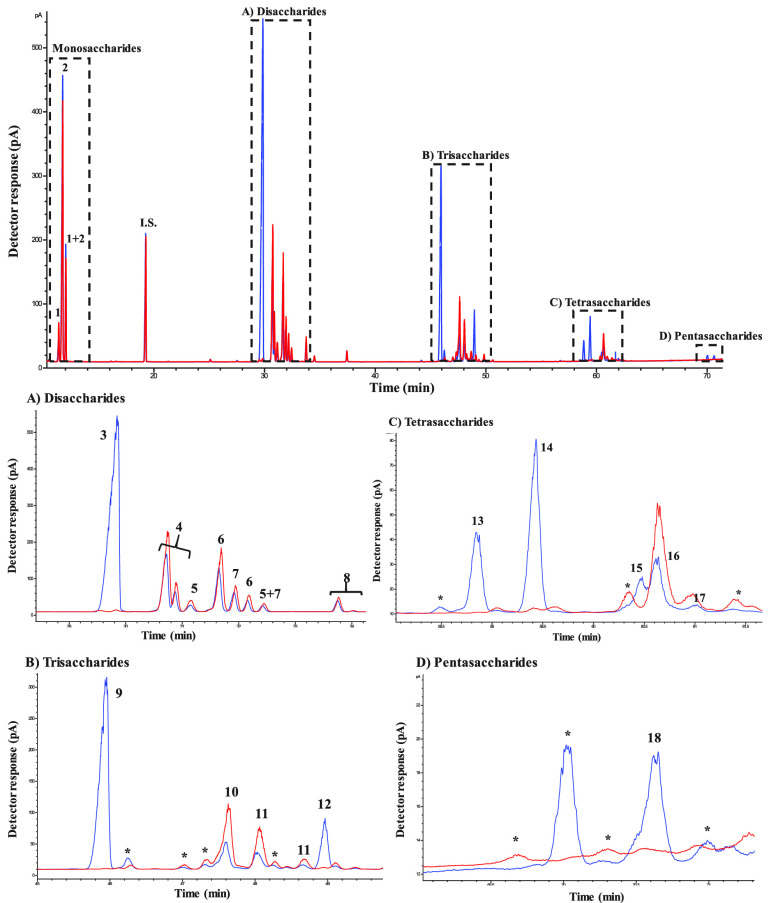
Chromatographic profiles obtained by GC-FID of TMSO derivatives of the transgalactosylation reaction after 24 h by β-galactosidase from *Bacillus circulans* using lactose/trehalose (blue) and lactose (red). Disaccharide (A), trisaccharide (B), tetrasaccharide (C), and pentasaccharide (D) fractions are shown for each reaction. Peaks: 1: galactose, 2: glucose, I.S.: internal standard, (A) 3: trehalose, 4: lactose, 5: β-d-galactopyranosyl-(1→4)-β-d-glucose, 6: β-d-galactopyranosyl-(1→3)-β-d-glucose, 7: β-d-galactopyranosyl-(1→2)-β-d-glucose, 8: β-d-galactopyranosyl-(1→6)-β-d-glucose, (B) 9: β-d-galactopyranosyl-(1→4)-d-trehalose, 10: β-d-galactopyranosyl-(1→4)-d-lactose, 11: β-d-galactopyranosyl-(1→6)-d-lactose, 12: β-d-galactopyranosyl-(1→6)-d-trehalose, (C) 13 and 15: β-d-galactopyranosyl-(1→4)-β-d-galactopyranosyl-(1→6)-d-trehalose or β-d-galactopyranosyl-(1→4)-α-d-glucopyranosyl-(1↔1)-[β-d-galactopyranosyl-(1→4)]-α-d-glucopyranoside, 14: β-d-galactopyranosyl-(1→4)-β-d-galactopyranosyl-(1→4)-d-trehalose, 16 and 17: β-d-galactopyranosyl-(1→4)-α-d-glucopyranosyl-(1↔1)-[β-d-galactopyranosyl-(1→6)]-α-d-glucopyranoside or β-d-galactopyranosyl-(1→6)-α-d-glucopyranosyl-(1↔1)-[β-d-galactopyranosyl-(1→6)]-α-d-glucopyranoside, and (D) 18: β-d-galactopyranosyl-(1→4)-β-d-galactopyranosyl-(1→4)-β-d-galactopyranosyl-(1→4)-d-trehalose. *Other saccharides with unknown structures.

The reaction conditions are different from those most commonly used for *Bacillus circulans* β-gal due to the major presence of the β-gal isoforms II and III in the Biolactase NTL Conc. Figure 1 shows the chromatographic GC-FID profile during the transgalactosylation assay after 24 h of reaction using lactose and trehalose and only lactose. In the first instance, a considerable amount of monosaccharides (peaks 1 and 2) was observed in the chromatogram due to the hydrolytic activity of the β-gal. Moreover, the transgalactosylation reaction was also observed (Figure 1A–D).

In the lactose assays, di- and trisaccharide β-GOS fractions (Figure 1A,B, red) were obtained and identified by GC–MS profiles (peaks 6–8, 10, and 11). These structures are known, as they were obtained by reactions with β-gal of different sources.^26,33^ On the other hand, new oligosaccharides were synthesized using trehalose as a carbohydrate acceptor (Figure 1, blue). These compounds correspond to galactosylated trehalose (Figure 1B, peaks 9 and 12; Figure 1C, peaks 13, 14, 15, 16, and 17). Interestingly, although there was a low quantity, the pentasaccharide fraction was also observed in the chromatogram (Figure 1D, peak 18).

**Figure 2:**
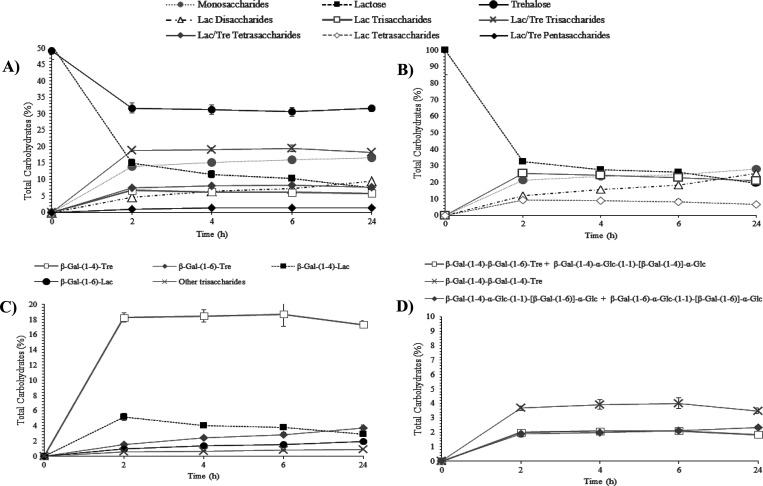
Evolution in the content of carbohydrates (%) during transgalactosylation reactions of lactose/trehalose (A) and lactose (B) solutions and evolution of tri- (C) and tetrasaccharides (D) (%) of the lactose/trehalose mixture. Reactions catalyzed by β-galactosidase from *Bacillus circulans* for 24 h at 50 °C, pH 4.5.

The kinetic behavior of the reaction mixtures, lactose/trehalose and lactose, is indicated in Figure 2A,B, respectively; moreover, quantitative data are shown in Table S1. After 24 h, 85.3 (Lac/Tre, Figure 2A) and 80.8% (Lac, Figure 2B) lactose was hydrolyzed, whereas trehalose decreased by 35.8% (Figure 2A). Regarding disaccharide synthesis, only β-GOS disaccharides derived from lactose were observed in both reactions, being produced in a higher amount using the lactose only mixture (23.3%, Figure 2B).

Trisaccharides were the main oligosaccharides generated during the lactose/trehalose reaction, especially those derived from trehalose (19.5% after 6 h), in contrast to those derived using only lactose (6.1%) (Figure 2A and Table S1). This result indicates that trehalose is a better acceptor than lactose. Just tetrasaccharides from trehalose were identified in the lactose/trehalose reaction (8.2%). A similar situation occurred with pentasaccharides, which were only formed in the trehalose/lactose mixture (Figure 2A; 1.4% after 24 h). The maximum formation of the trehalose derivatives occurred between 6 and 24 h of the incubation time (Figure 2A). These data support the efficiency and the suitability of the β-gal to produce trehalose derivatives. β-GOS tri- and tetrasaccharide productions were 21.0 and 6.5% after 24 h, respectively, using lactose as the sole donor and acceptor (Figure 2B), which is in line with previously reported β-GOS production.^27,34^

Tri-, tetra-, and pentasaccharide fractions were isolated by HILIC-RID and characterized by NMR. This characterization was accomplished by the combined use of 1D and 2D [^1^H, ^1^H] and [^1^H, ^13^C] NMR experiments (gCOSY, TOCSY, ROESY, multiplicity-edited gHSQC, bsgHMBC, and hybrid experiment gHSQC-TOCSY). ^1^H and ^13^C NMR chemical shifts were observed, and the structures of each compound are summarized and numbered in Tables 1–4. Full sets of spectra are available in the Supporting Information (Figures S1–S43).

**Table 1:**
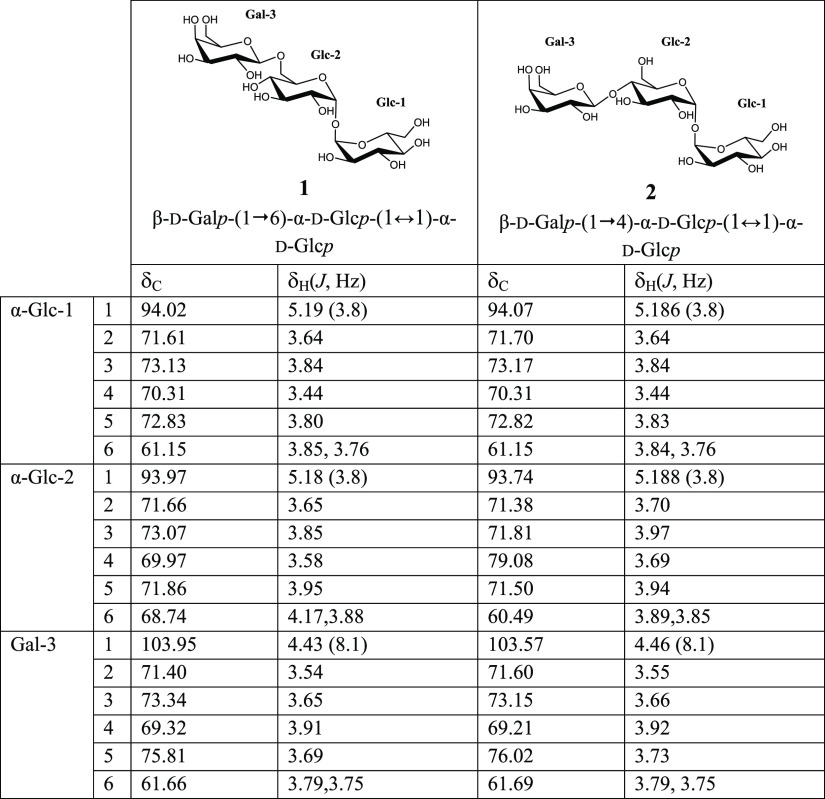
^1^H (500 MHz) and ^13^C (125 MHz) NMR Chemical Shifts (δ, ppm) and Coupling Constants (*J* in Hz, in Parentheses) Determined by 1D and 2D NMR Spectroscopy of Trisaccharides **1** and **2**

**Table 2:**
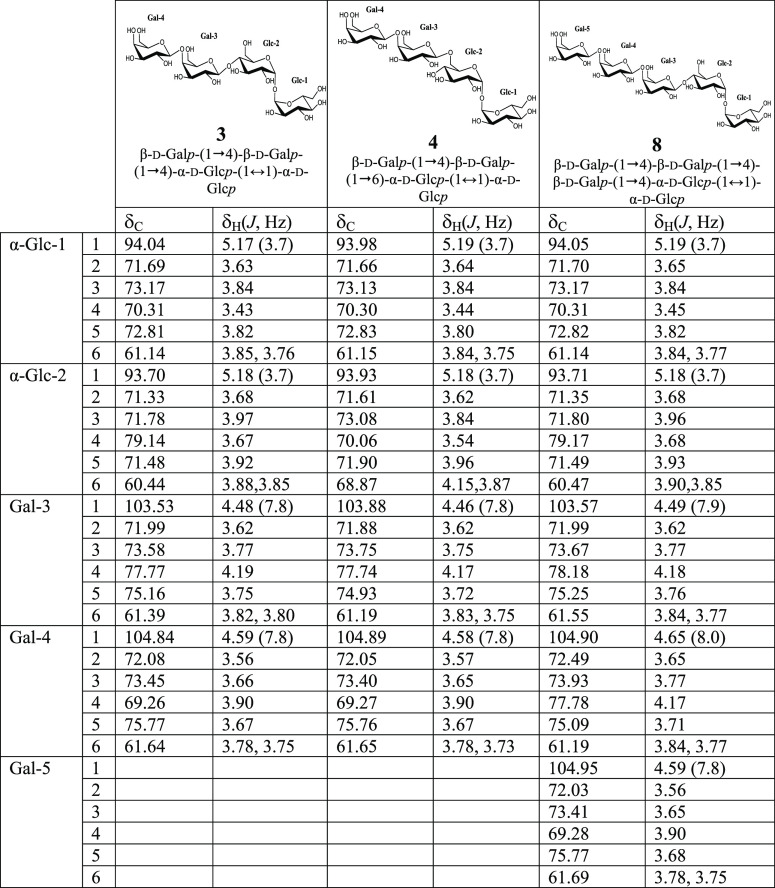
^1^H (500 MHz) and ^13^C (125 MHz) NMR Chemical Shifts (δ, ppm) and Coupling Constants (*J* in Hz, in Parentheses) Determined by 1D and 2D NMR Spectroscopy of Tetrasaccharides **3** and **4** and Pentasaccharide **8**, with Terminal Trehalose

**Table 3:**
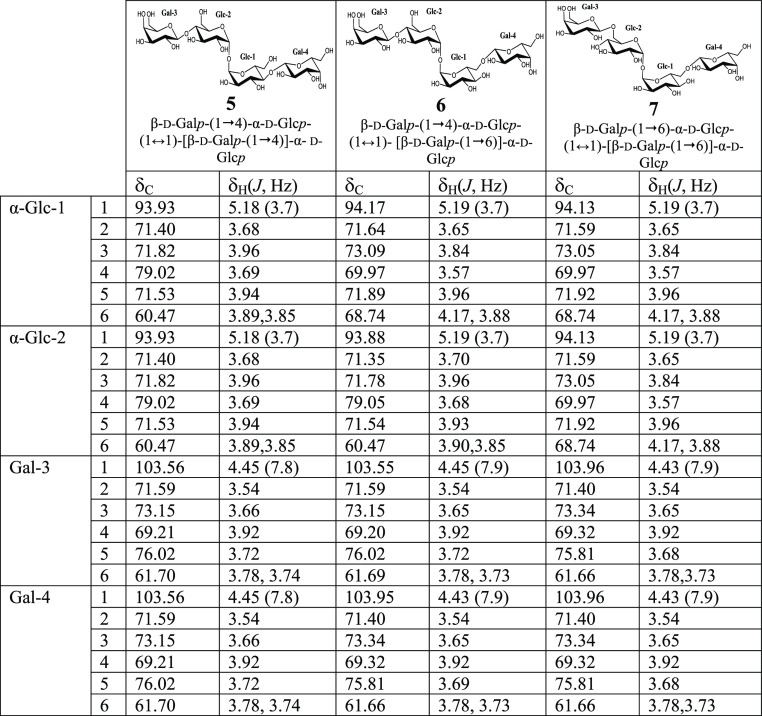
^1^H (500 MHz) and ^13^C (125 MHz) NMR Chemical Shifts (δ, ppm) and Coupling Constants (*J* in Hz, in Parentheses) Determined by 1D and 2D NMR Spectroscopy of Tetrasaccharides **5**, **6**, and **7**, with Central Trehalose

**Table 4:**
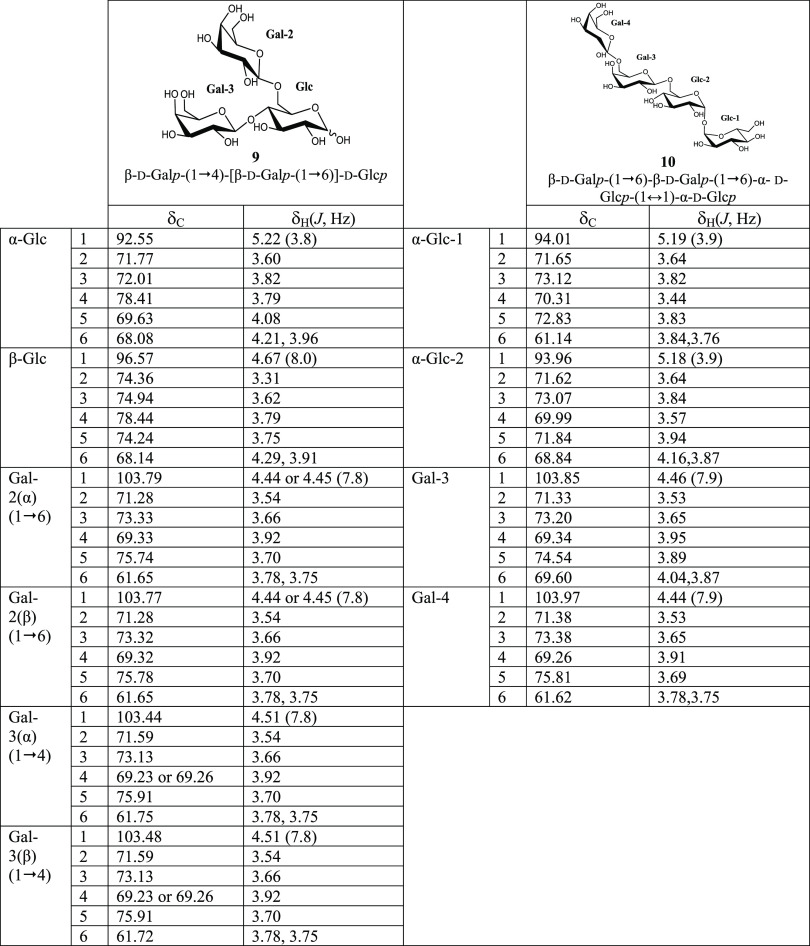
^1^H (500 MHz) and ^13^C (125 MHz) NMR Chemical Shifts (δ, ppm) and Coupling Constants (*J* in Hz, in Parentheses) Determined by 1D and 2D NMR Spectroscopy of Oligosaccharides **9** and **10**

Two compounds could be identified in the trisaccharide fraction (Figure 1B). Compound **1** (peak 12) was assigned as β-d-galactopyranosyl-(1→6)-α-d-glucopyranosyl-(1↔1)-α-d-glucopyranoside [β-d-galactopyranosyl-(1→6)-α,α-trehalose] (Table 1, compound **1**). The 1D ^1^H NMR spectrum of this compound (see Supporting Information, Figure S1) showed three doublets in the anomeric region (δ 5.19, ^3^*J*_H1,H2_ = 3.8 Hz, δ 5.18, ^3^*J*_H1,H2_ = 3.8 Hz, and δ 4.43, ^3^*J*_H1,H2_ = 8.1 Hz), corresponding to both α-glucoses and β-galactose, respectively. In addition, the 1D ^13^C NMR spectrum showed signals corresponding to 18 carbons, including three anomeric carbons (δ 94.02, δ 93.97, and δ 103.95). A multiplicity-edited gHSQC spectrum was used to link the carbon signals to the corresponding proton resonances. It showed three anomeric carbons, 12 CH, and three methylene carbons. In addition, COSY, TOCSY, and gHSQC-TOCSY experiments supported the presence of two α-glucose units and one β-galactose unit. The position of glycosidic linkages was analyzed from the bsgHMBC spectrum. It showed correlations between the two α-Glc anomeric positions, confirming the presence of a trehalose unit. In addition, relevant correlation bands between the β-Gal-3-C1 anomeric carbon and α-Glc-2-H6 and between the α-Glc-2-C6 methylene carbon and the β-Gal-3-H1 anomeric proton could be found. These results confirmed the proposed structure. This compound has been previously characterized through NMR by Ajisaka and Fujimoto^35^ and by Kim et al.^25^ Some differences in chemical shifts are found, but in the first case, acetonitrile was used as an internal reference in D_2_O, and in the second one, DMSO-*d*_6_ was used as a solvent, which could explain these little changes.

Regarding compound **2** (peak 9, Figure 1B), it was assigned as β-d-galactopyranosyl-(1→4)-α-d-glucopyranosyl-(1↔1)-α-d-glucopyranoside [β-d-galactopyranosyl-(1→4)-α,α-trehalose] (Table 1, compound **2**). 1D and 2D spectra were analyzed as in the previous case (see Supporting Information, Figures S7–S11), and they also supported the presence of two α-glucose units and one β-galactose unit. All ^1^H and ^13^C signals could be assigned (Table 1). The results confirmed the structure as β-d-galactopyranosyl-(1→4)-α-d-glucopyranosyl-(1↔1)-α-d-glucopyranoside, and they are in accordance with other data from Ishii et al.^36^

In the case of tetrasaccharide fractions, we have been able to characterize two types of tetrasaccharides: two of them with a terminal trehalose in their structure (Table 2, compounds **3** and **4**) and three more where trehalose is situated in the center (Table 3, compounds **5**, **6**, and **7**).

Compound **3** (peak 14, Figure 1C) was assigned as β-d-galactopyranosyl-(1→4)-β-d-galactopyranosyl-(1→4)-α-d-glucopyranosyl-(1↔1)-α-d-glucopyranoside [β-d-galactopyranosyl-(1→4)-β-d-galactopyranosyl-(1→4)-α,α-trehalose] (Table 2, compound **3**). The 1D ^1^H NMR spectrum (see Supporting Information, Figure S12 and Table 2) showed four doublets in the anomeric region (δ 5.17, ^3^*J*_H1,H2_ = 3.7 Hz, δ 5.18, ^3^*J*_H1,H2_ = 3.7 Hz, δ 4.48, ^3^*J*_H1,H2_ = 7.8 Hz, and δ 4.59, ^3^*J*_H1,H2_ = 7.8 Hz), corresponding to both α-glucoses from trehalose units and two β-galactoses. The 1D ^13^C NMR spectrum showed signals corresponding to 24 carbons, including four anomeric carbons (δ 94.04, δ 93.70, δ 103.53, and δ 104.84). 2D spectra also supported the presence of two α-glucose and two β-galactose units. The position of glycosidic linkages was analyzed from the bsgHMBC spectrum. It showed correlations between the two α-Glc anomeric positions, confirming the presence of a trehalose unit, and in addition, relevant correlations between the β-Gal-3-C1 anomeric carbon and α-Glc-2-H4 and between α-Glc-2-C4 and β-Gal-3-H1 anomeric protons were found. Moreover, key correlations between the β-Gal-4-C1 anomeric carbon and β-Gal-3-H4 and between β-Gal-3-C4 and β-Gal-4-H1 anomeric protons showed a (1→4) linkage between two galactose units, confirming the proposed structure.

Compound **4** was obtained together with compound **5** by HILIC-RID, as a 1:1.5 mixture, but despite this, both compounds could be assigned by NMR unequivocally, differentiating signals of each one in the spectra (see Supporting Information, Figures S18–S24). Following the same procedure as before, compound **4** (peaks 13 and 15, Figure 1C) was assigned as β-d-galactopyranosyl-(1→4)-β-d-galactopyranosyl-(1→6)-α-d-glucopyranosyl-(1↔1)-α-d-glucopyranoside [β-d-galactopyranosyl-(1→4)-β-d-galactopyranosyl-(1→6)-α,α-trehalose] (Table 2, compound **4**). The position of glycosidic linkages was established, as in the previous case, with corresponding key correlations analyzed from bsgHMBC spectra. They showed a (1→4) linkage between the two galactose units, but in this case, the linkage between Gal-3 and the trehalose unit was (1→6).

As mentioned before, tetrasaccharides **5**, **6**, and **7** present a different structure, where the trehalose unit is situated in the center of the molecule. In these cases, every galactose unit is linked to a different glucose unit of the trehalose. Compound **5** (peaks 13 and 15, Figure 1C), assigned as β-d-galactopyranosyl-(1→4)-α-d-glucopyranosyl-(1↔1)-[β-d-galactopyranosyl-(1→4)]-α-d-glucopyranoside, is a symmetric compound, with a (1→4) linkage between every β-galactose and α-glucose (Table 3, compound **5**). Due to its symmetry, chemical shifts are the same for both galactoses and for both glucoses in the molecule. It showed two doublets in the anomeric region (δ 5.18, ^3^*J*_H1,H2_ = 3.7 Hz and δ 4.45, ^3^*J*_H1,H2_ = 7.8 Hz), corresponding to both α-glucoses from the trehalose unit and two β-galactoses, respectively. The 1D ^13^C NMR spectrum showed signals corresponding to 12 carbons (due to the symmetry of the molecule), including two anomeric carbons (δ 93.93 and δ 103.56). 2D spectra, together with ^13^C chemical shifts and intensity of the signals (see Supporting Information, Figures S19 and S21), supported the presence of two α-glucose and two β-galactose units. The (1→4) linkage between every β-galactose and α-glucose was determined by the bsgHMBC spectrum showing correlations between the β-Gal-C1 anomeric carbons and α-Glc-H4 and between α-Glc-C4 and β-Gal-H1 anomeric protons. Correlations between the two α-Glc anomeric positions supported the presence of the trehalose unit.

Compounds **6**, **7**, and **8** were part of the same fraction obtained by HILIC-RID, as a 3.5:2.7:1 mixture; however, all compounds could be assigned by NMR unequivocally, differentiating signals of each one in the spectra (see Supporting Information, Figures S25–S32).

In compound **6** (peaks 16 and 17, Figure 1C), assigned as β-d-galactopyranosyl-(1→4)-α-d-glucopyranosyl-(1↔1)-[β-d-galactopyranosyl-(1→6)]-α-d-glucopyranoside, three doublets in the anomeric region are shown (δ 5.19, ^3^*J*_H1,H2_ = 3.7 Hz, δ 4.45, ^3^*J*_H1,H2_ = 7.9 Hz, and δ 4.43, ^3^*J*_H1,H2_ = 7.9 Hz). They corresponded to both α-glucoses from the trehalose unit and two β-galactoses (Table 3, compound **6**). The 1D ^13^C NMR spectrum showed signals corresponding to 24 carbons, including four anomeric carbons (δ 94.17, δ 93.88, δ 103.55, and δ 103.95). 2D spectra also supported the presence of two α-glucose and two β-galactose units, and according to correlations shown in the bsgHMBC spectra, it could be affirmed that one galactose was linked to position 4 of one of the glucose units of the central trehalose, and the other galactose was linked to position 6 of the other glucose unit.

Compound **7** (peaks 16 and 17, Figure 1C), assigned as β-d-galactopyranosyl-(1→6)-α-d-glucopyranosyl-(1↔1)-[β-d-galactopyranosyl-(1→6)]-α-d-glucopyranoside, is a symmetric compound, as compound **5**, and it was assigned following the same strategy (Table 3, compound **7**). This time, the bsgHMBC spectrum showed correlations between the β-Gal-C1 anomeric carbons and α-Glc-H6 and between α-Glc-C6 and β-Gal-H1 anomeric protons, besides correlations between the two α-Glc anomeric positions, supporting the proposed structure.

Compound **8** (peak 18, Figure 1D) was assigned as the pentasaccharide β-d-galactopyranosyl-(1→4)-β-d-galactopyranosyl-(1→4)-β-d-galactopyranosyl-(1→4)-α-d-glucopyranosyl-(1↔1)-α-d-glucopyranoside (β-d-galactopyranosyl-(1→4)-β-d-galactopyranosyl-(1→4)-β-d-galactopyranosyl-(1→4)-α,α-trehalose) (Table 2, compound **8**). In this case, the 1D ^1^H NMR spectrum (see Supporting Information, Figure S25 and Table 2) showed five doublets in the anomeric region (δ 5.19, ^3^*J*_H1,H2_ = 3.7 Hz, δ 5.18, ^3^*J*_H1,H2_ = 3.7 Hz, δ 4.49, ^3^*J*_H1,H2_ = 7.9 Hz, δ 4.65, ^3^*J*_H1,H2_ = 8.0 Hz, and δ 4.59, ^3^*J*_H1,H2_ = 7.8 Hz), corresponding to both α-glucoses from the trehalose unit and three β-galactoses. The 1D ^13^C NMR spectrum showed signals corresponding to 30 carbons, including five anomeric carbons (δ 94.05, δ 93.71, δ 103.57, δ 104.90, and δ 104.95). 2D spectra also supported the presence of two α-glucose and three β-galactose units. In this case, trehalose is situated at the end of the chain, and the position of glycosidic linkages was analyzed from bsgHMBC spectra, being always (1→4) between every galactose unit and the next one of the chains and between galactose-3 and trehalose. Relevant correlations to support this affirmation were β-Gal-5-C1 anomeric carbon and β-Gal-4-H4, β-Gal-4-C4 and β-Gal-5-H1 anomeric protons, β-Gal-4-C1 anomeric carbon and β-Gal-3-H4, β-Gal-3-C4 and β-Gal-4-H1 anomeric protons, β-Gal-3-C1 anomeric carbon and α-Glc-2-H4, α-Glc-2-C4 and β-Gal-3-H1 anomeric protons, and between the two α-Glc anomeric positions, confirming the presence of a trehalose unit.

These results sustained the obtainment of two new trisaccharides derived from trehalose. The structure was confirmed to be as β-d-galactopyranosyl-(1→6)-d-trehalose (Table 1, compound **1**) and β-d-galactopyranosyl-(1→4)-d-trehalose (Table 1, compound **2**). β-Gal of *E. coli* was used by Kim et al.^25^ to generate trehalose derivatives. That work revealed that the main products were β-gal-(1→4)-trehalose and β-gal-(1→6)-trehalose, which are in good agreement with the results of Figure 1. In addition, this type of linkage is very similar to the trisaccharides contained in commercial dietary β-GOS. Van Leeuwen et al.^37^ compared the structure of different commercially available GOS trisaccharides, indicating that bonds β-(1→4) and β-(1→6) were the most abundant, depending on the microbial enzyme used in the synthesis. Specifically, Figure 2C shows the individual evolution of the trisaccharides synthesized in the lactose/trehalose reaction. The synthesis yield of each oligosaccharide increased with reaction time, observing the highest increase after 2 h of incubation and then reaching a plateau with a progressive synthesis of trisaccharides. The main product obtained was β-Gal-(1→4)-Tre (∼18.7%) (Figure 2C). These results seem to indicate that β-gal from *Bacillus circulans* prioritize to join the galactose from lactose (used as a donor) in the trehalose with a linkage β-(1→4) and in a lesser extent β-(1→6). The same structures of trisaccharides derived from trehalose were also obtained by Ishii et al.^36^ using *B. circulans* β-gal. This indicates the effectiveness of transgalactosylation mechanisms of β-gal of *B. circulans*.^38^

New tetrasaccharides were found in this work [β-d-galactopyranosyl-(1→4)-β-d-galactopyranosyl-(1→4)-d-trehalose and β-d-galactopyranosyl-(1→4)-β-d-galactopyranosyl-(1→6)-d-trehalose] (Table 2). These results followed the same pattern as the trisaccharides synthesized, where the galactose monomers of lactose have been driven to the acceptor molecule galactosyl-trehalose and linked by β-(1→6) and β-(1→4) bonds. Figure 2D indicates that similar quantities of the different tetrasaccharides were obtained. Therefore, quantitative results (Table S1) show a greater production after 6 h of reaction (8.2%). Remarkably, an interesting mixture of structures where the trehalose unit is situated in the center of the molecule was also synthesized (Table 3, compounds **5**, **6**, and **7**). In this case, the trehalose has been galactosylated in both terminal endings resulting in three different tetrasaccharides distinguished by both types of bonds. This fact could indicate that the trisaccharides β-Gal-(1→4)-Tre and β-Gal-(1→6)-Tre also acted as acceptors. Furthermore, the slight differences between these chemical structures may be important in a possible production in a major scale.

The majority of the studies based on the synthesis of trehalose derivatives focused on the synthesis of disaccharides analogues, such as lactotrehalose or mannotrehalose,^39,40^ or trisaccharides, as the structures named above;^25,36^ however, no tetra- or pentasaccharides were investigated. Chaube et al.^41^ were the first to synthesize tetrasaccharides trehalose-derivatives from *Mycobacterium smegmatis* obtaining galactosyl and glucosyl structures linked by α-(1→6) and β-(1→6) to trehalose moieties, respectively. Nevertheless, the process of synthesis was arduous and complex, using organic solvents. On the other hand, Wessel and Niggemann^42^ synthesized different types of trehalose oligosaccharides with glucose linked via β-(1→4) and DP up to 5. To the best of our knowledge, our data are the first evidence on the synthesis of tetra- and pentasaccharide trehalose galactosylated using commercially available enzymes.

### 3.2 Transgalactosylation by β-Galactosidase from *Aspergillus oryzae*

**Figure 3:**
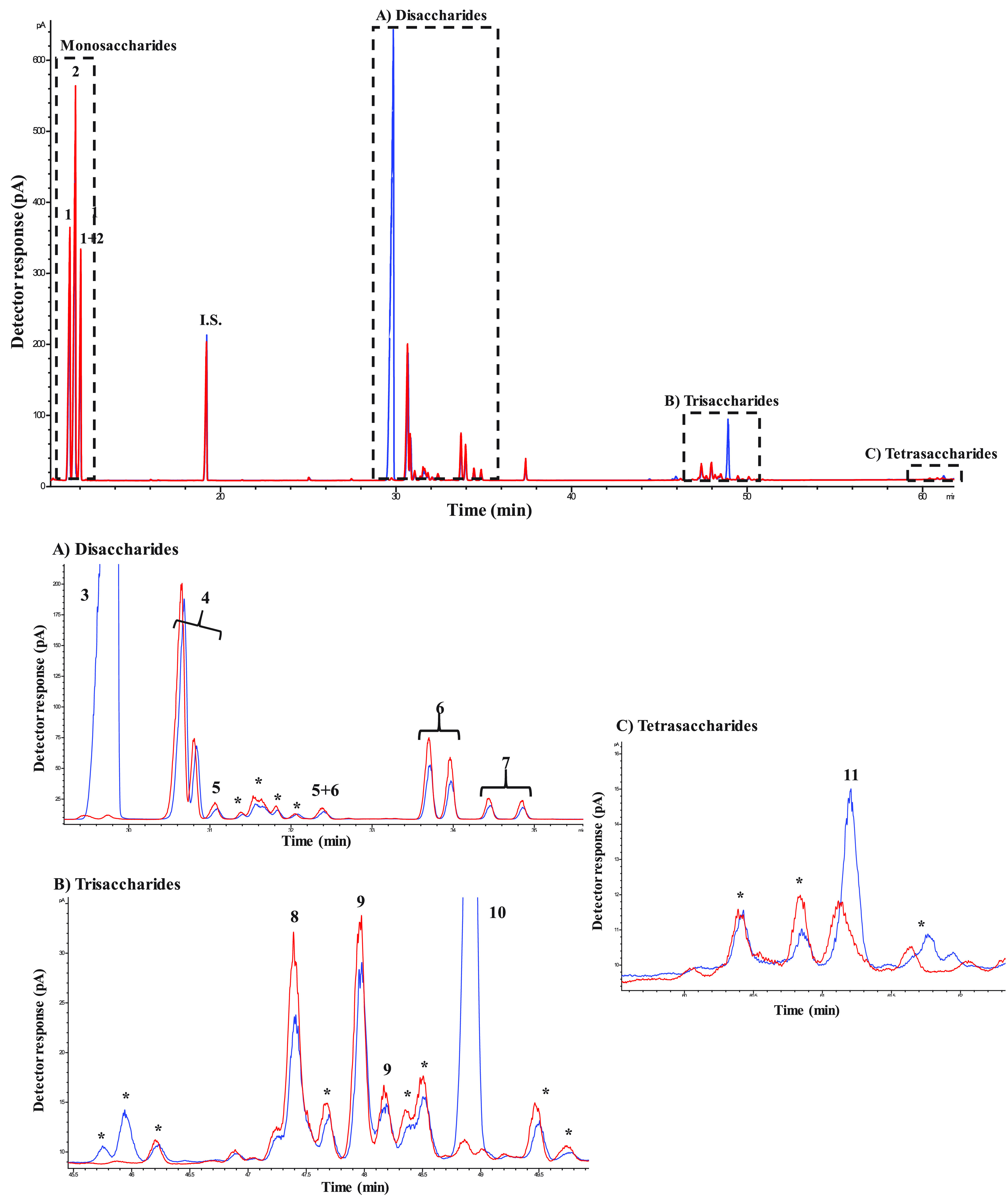
Chromatographic profiles obtained by GC-FID of TMSO derivatives of the transgalactosylation reaction after 6 h by β-galactosidase from *Aspergillus oryzae* using lactose/trehalose (blue) and lactose (red). Disaccharide (A), trisaccharide (B), and tetrasaccharide (C) fractions are shown for each reaction. Peaks: 1: galactose, 2: glucose, I.S.: internal standard, (A) 3: trehalose, 4: lactose, 5: β-d-galactopyranosyl-(1→4)-β-d-galactose, 6: β-d-galactopyranosyl-(1→6)-β-d-glucose, 7: β-d-galactopyranosyl-(1→6)-β-d-galactose, (B) 8: β-d-galactopyranosyl-(1→4)-[β-d-galactopyranosyl-(1→6)]-d-glucose, 9: β-d-galactopyranosyl-(1→6)-lactose, 10: β-galactopyranosyl-(1→6)-trehalose, and (C) 11: β-galactopyranosyl-(1→6)-β-galactopyranosyl-(1→6)-trehalose. *Other saccharides with unknown structures.

Figure 3 shows the GC-FID chromatogram during the transgalactosylation assay after 6 h of reaction using the lactose/trehalose mixture and only lactose by β-gal from *A. oryzae*. The GC-FID profile of *A. oryzae* (Figure 3) shows a less complex mixture than *B. circulans*, as a result of the less formation of disaccharides (Figure 3A), trisaccharides (Figure 3B), and tetrasaccharides (Figure 3C), including β-GOS and trehalose derivatives. In the lactose assay (Figure 3, red), β-GOS di- (peaks 5–7; Figure 3A) and trisaccharides (peaks 8–10, Figure 3B) were detected, in a higher level than the lactose/trehalose mixture. Tetrasaccharides were found in both reactions, in a substantially low level (multiple peaks labeled as 11 and *; Figure 3C). Remarkably, a series of unknown compounds were also detected in the lactose/trehalose mixture (Figure 3, blue): saccharides labeled as 10 and 11 (Figure 3) were only synthesized when trehalose was present on the reaction.

**Figure 4:**
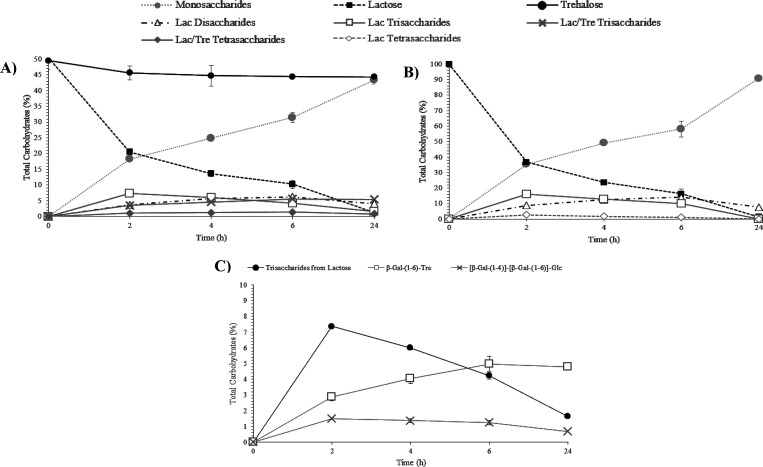
Evolution in the content of carbohydrates (%) during transgalactosylation reactions of lactose/trehalose (A) and lactose (B) solutions and (C) evolution in the content of trisaccharides (%) of the lactose/trehalose mixture. Reactions catalyzed by β-galactosidase from *Aspergillus oryzae* for 24 h at 50 °C, pH 4.

The evolution in the content of carbohydrates for each reaction is observed in Figure 4A (lactose/trehalose) and Figure 4B (lactose). Table S2 shows the quantitative data of the assays. A progressive and complete hydrolysis of lactose after 24 h was observed in both reactions, while the corresponding monosaccharides increased (peaks 1 and 2, Figure 3A) with the incubation time. In the lactose/trehalose mixture (Figure 4A), the maximum formation of the di- and trisaccharides occurred between 2 and 6 h of reaction. As observed in the reaction with *B. circulans*, only disaccharides from lactose were synthesized, reaching a maximum of 14.1% after 6 h in the lactose mixture versus 6.3% obtained when lactose/trehalose was used. The presence of trehalose appeared to decrease the β-GOS formation in favor to giving rise to the new oligosaccharides. The less formation of disaccharides in Figure 4A was balanced for the synthesis of the trehalose trisaccharides (5.6% after 6 h). Trehalose levels were diminished in a lower degree than the *B. circulans* reaction (Figure 2A); therefore, the synthesis of trehalose-derived oligosaccharides was lower. It has been reported that the transgalactosylation properties of β-gal from *A. oryzae* are less effective than those of *E. coli* and *B. circulans* in terms of trehalose analogues.^35,36^ Remarkably, despite the low quantity of tetrasaccharides (1.4%) observed in Figure 4A, new oligosaccharides were synthesized. The new oligosaccharides were isolated by HILIC-RID and characterized by NMR.

NMR characterization was accomplished as before by the combined use of 1D and 2D [^1^H, ^1^H] and [^1^H, ^13^C] NMR experiments (gCOSY, TOCSY, ROESY, multiplicity-edited gHSQC, bsgHMBC, and hybrid experiment gHSQC-TOCSY). ^1^H and ^13^C NMR chemical shifts observed are summarized in Tables 1 and 4. Full sets of spectra are available in the Supporting Information (Figures S1–S6 and S33–S43). Peak 10 (Figure 3B) was identified as compound **1** [β-d-galactopyranosyl-(1→6)-α,α-trehalose]. All NMR spectra were identical to those for the trisaccharide already described in the previous section.

Interestingly, in the case of trisaccharide **9** (peak 8, Figure 3B), the α,α-trehalose moiety was not found. The 1D ^1^H NMR spectrum (see Supporting Information, Figure S33 and Table 4) showed five doublets in the anomeric region. They belong to two sets of signals, corresponding to an equilibrium of the α:β anomers of the terminal glucose (δ 5.22, ^3^*J*_H1,H2_ = 3.8 Hz, δ 4.44 or 4.45, ^3^*J*_H1,H2_ = 7.8 Hz, and δ 4.51, ^3^*J*_H1,H2_ = 8.1 Hz for the α anomer and δ 4.67, ^3^*J*_H1,H2_ = 8.0 Hz, δ 4.44 or 4.45, ^3^*J*_H1,H2_ = 7.8 Hz, and δ 4.51, ^3^*J*_H1,H2_ = 8.1 Hz for the β anomer). The 1D ^13^C NMR spectrum showed signals corresponding to 31 carbons (five of them including two carbons in the same signal). The gHSQC spectrum was used to link the carbon signals to the corresponding proton resonances. It showed six anomeric carbons (δ 92.55, δ 96.57, δ 103.79, δ 103.77, δ 103.44, and δ 103.48), 20 CH (four pairs occur under the same signal), and five methylene carbons (two of them are under the same signal). In addition, COSY, TOCSY, and gHSQC-TOCSY experiments supported the presence of two β-galactose units and one glucose unit (α and β forms) for each trisaccharide of the pair. The position of glycosidic linkages was analyzed from bsgHMBC spectra. In this case, it showed correlations between the anomeric carbons of the two β-Gal units with protons in positions 4 and 6 of the same glucose unit for each anomeric form. Therefore, relevant correlation bands between the β-Gal-3-C1 anomeric carbon and α/β-Glc-H4 and between α/β-Glc-C4 carbon and the β-Gal-3-H1 anomeric proton could be found. Also, it showed correlations between β-Gal-2-C1 anomeric carbon and α/β-Glc-H6 methylene protons and between α/β-Glc-C6 methylene carbon and the β-Gal-2-H1 anomeric proton. These results confirmed the structure as β-d-galactopyranosyl-(1→4)-[β-d-galactopyranosyl-(1→6)]-d-glucopyranose (Table 4, compound **9**).

Carrying out a similar analysis, compound **10** (peak 11, Figure 3C) was assigned as β-d-galactopyranosyl-(1→6)-β-d-galactopyranosyl-(1→6)-α-d-glucopyranosyl-(1↔1)-α-d-glucopyranoside [β-d-galactopyranosyl-(1→6)-β-d-galactopyranosyl-(1→4)-α,α-trehalose] (see Supporting Information, Figures S39–S43 and Table 4, compound **10**). Key correlations in 2D spectra confirmed the presence of a trehalose unit and also the position of glycosidic linkages.

It should be noted that compound **10** was obtained together with compound **11**, in a 2:1 mixture. In this case, not all signals for compound **11** could be assigned, but signals observed in all spectra were consistent with the assignation of this compound as previously described,^43^ the trisaccharide β-d-galactopyranosyl-(1→6)-β-d-galactopyranosyl-(1→4)-d-glucopyranose (see Supporting Information, Figures S39–S43).

The synthesis of the lactose/trehalose mixture promoted considerably the formation of two trisaccharides, reaching maximum yields at 6 h of reaction (Figure 4C). β-d-Galactopyranosyl-(1→4)-[β-d-galactopyranosyl-(1→6)]-d-glucopyranose (Table 4, compound **9**) was the main product obtained in the transgalactosylation assay; this β-GOS trisaccharide has been also found by Yin et al.^44^ using lactose as a donor and acceptor. The other trisaccharide was the same as that obtained by *B. circulans*, β-Gal*p*-(1→6)-Tre (Table 4, compound **1**), but in a lower quantity (Figure 4C). These data are in line with Urrutia et al.^45^ who observed a high preference of β-gal of *A. oryzae* for the formation of β-(1→6) bonds, as well as for *B. circulans*. Moreover, the low level of production using the *A. oryzae* enzyme is in good agreement with Ajisaka and Fujimoto,^35^ who carried out a total synthesis yield of 7%. On the other hand, a new tetrasaccharide was obtained in the reaction, whose structure is shown in Table 4 (Compound **10**). As well as in *B. circulans*, these are the first data reported of galactosylated trehalose tetrasaccharides synthesized by commercial enzymes. In addition, Ferreira-Lazarte et al.^46^ studied the digestibility of β-GOS with different types of bonds using brush border membrane vesicles from pig, which contains the disaccharidases responsible for the digestion of dietary sugars. Their findings revealed that the β-(1→6) linkage showed the highest resistance to digestion (12% after 3 h) followed by β-(1→4) (26%) and β-(1→3) (40%). This supports the hypothesis that the new trehalose tri- and tetrasaccharides could be less prone to hydrolysis.

The *B. circulans* enzyme seemed to have a greater production of potential new trehalose derivatives, including tri- and tetrasaccharides (19.5 and 8.2%, respectively). The main synthesized trisaccharides were β-Gal*p*-(1→4)-Tre and β-Gal*p*-(1→6)-Tre, obtained by both enzymes. On the other hand, tetrasaccharides were produced in a higher quantity while using the *B. circulans* enzyme and showed the most diverse structures. The *A. oryzae* enzyme only synthesized β-Gal*p*-(1→6)-β-Gal*p*-(1→6)-Tre in a lower amount. In addition, a tetrasaccharide was also found: β-Gal*p*-(1→6)-β-Gal*p*-(1→6)-Tre. β-(1→6) and β-(1→4) Galactosyl linkages are minimally digested in the small intestine; therefore, the virulence mediated by negative microorganisms, such as *C. difficile*, in the gut could be reduced. In addition, these new structures could have a key role in the proper beneficial effects of trehalose, such as autophagy or glycemic control. Data obtained in this wok could be useful to the production of trehalose derivatives in a major scale, concerning the selectivity of each β-gal from different sources. In this context, increasing the knowledge in terms of the digestibility of this new compound is necessary to understand the real behavior of this carbohydrate in the digestive system.

## Supporting Information Available

Quantitative data of carbohydrate evolution during the transgalactosylation assays with lactose/trehalose and lactose solutions by β-galactosidase from *Bacillus circulans* (Table S1) and *Aspergillus oryzae* (Table S2) and full NMR spectra of each trehalose-based oligosaccharide (1 to 11) (Figures S1 to S43) (PDF)

## Abbreviations

β-Gal: β-galactosidase
bsgHMBC: band-selective gradient heteronuclear multiple bond correlation
DP: degree of polymerization
Gal: galactose
GC-FID: gas chromatography-flame ionization detector
GC–MS: gas chromatography–mass spectrometry
gHSQC: gradient heteronuclear single-quantum coherence
Glc: glucose
GOS: galactooligosaccharides
HILIC-RID: hydrophilic interaction liquid chromatography-refractive index detector
NMR: nuclear magnetic resonance
Tre: trehalose
