# Supplementary material for: Enzymatic Synthesis and Structural Characterization of Novel Trehalose-Based Oligosaccharides
Source: J Agric Food Chem. 2021 Oct 12;69(42):12541–53. doi: 10.1021/acs.jafc.1c03768 (PMC8554766; doi:10.1021/acs.jafc.1c03768)
Supplement: Supplementary file 1 — jf1c03768_si_001.pdf [file jf1c03768_si_001.pdf]

Supporting Information

Enzymatic synthesis and structural characterization of novel trehalose-based oligosaccharides

Pablo Gallego-Lobillo[1], Elisa G. Doyagüez[2], María Luisa Jimeno[2], Mar Villamiel[1,]*, Oswaldo Hernandez-Hernandez[1]

[1] Institute of Food Science Research (CIAL), Spanish Council of Scientific Research, (CSIC)−Autonumous University of Madrid (UAM), c/Nicolás Cabrera, 9, Campus de la Universidad Autónoma de Madrid, E-28049, Madrid, Spain

[2] Centro de Química Orgánica "Lora Tamayo" (CSIC), c/Juan de la Cierva, 3, E-28006, Madrid, Spain

*Author to whom correspondence should be addressed: M. Villamiel

Institute of Food Science Research (CIAL) (CSIC-UAM), C/Nicolás Cabrera 9, Campus de la Universidad Autónoma de Madrid, E-28049 Madrid, Spain.

Tel: +34 910017951; E-mail: m.villamiel@csic.es

Table of Contents

Table S1. Carbohydrate Evolution during the Transgalactosylation Assay with Lactose/Trehalose and Lactose Solutions, by β-Galactosidase from *Bacillus circulans* at 50 °C, pH 4.5, Determined by GC-FID Analysis (% total carbohydrates)

Table S2. Carbohydrate Evolution during the Transgalactosylation Assay with Lactose/Trehalose and Lactose Solutions, by β-Galactosidase from *Aspergillus oryzae* at 50 °C, pH 4.5, Determined by GC-FID Analysis (% total carbohydrates)

NMR spectra:

Figure S1. ^{1}H NMR (500 MHz, D_2O) of trisaccharide **1** derived from trehalose.
Figure S2. ^{13}C NMR (125 MHz, D_2O) of trisaccharide **1** derived from trehalose.
Figure S3. Complete assignment of ^{13}C NMR spectrum of trisaccharide **1** derived from trehalose.
Figure S4. gCOSY and TOCSY (500 MHz, D_2O) of trisaccharide **1** derived from trehalose.
Figure S5. Multiplicity-edited gHSQC and gHMBC semiselective (500 MHz, D_2O) of trisaccharide **1** derived from trehalose.
Figure S6. ROESY (500 MHz, D_2O) of trisaccharide **1** derived from trehalose.
Figure S7. ^{1}H NMR (500 MHz, D_2O) of trisaccharide **2** derived from trehalose.
Figure S8. ^{13}C NMR (125 MHz, D_2O) of trisaccharide **2** derived from trehalose.
Figure S9. Complete assignment of ^{13}C NMR spectrum of trisaccharide **2** derived from trehalose.
Figure S10. gCOSY and TOCSY (500 MHz, D_2O) of trisaccharide **2** derived from trehalose.
Figure S11. Multiplicity-edited gHSQC and gHMBC semiselective (500 MHz, D_2O) of trisaccharide **2** derived from trehalose.
Figure S12. ^{1}H NMR (500 MHz, D_2O) of trisaccharide **3** derived from trehalose.
Figure S13. ^{13}C NMR (125 MHz, D_2O) of trisaccharide **3** derived from trehalose.
Figure S14. Complete assignment of ^{13}C NMR spectrum of trisaccharide **3** derived from trehalose.
Figure S15. gCOSY and TOCSY (500 MHz, D_2O) of trisaccharide **1** derived from trehalose.
Figure S16. Multiplicity-edited gHSQC and gHMBC semiselective (500 MHz, D_2O) of trisaccharide **3** derived from trehalose.
Figure S17. ROESY (500 MHz, D_2O) of trisaccharide **3** derived from trehalose.
Figure S18. ^{1}H NMR (500 MHz, D_2O) for the mixture of tetrasaccharides **4** and **5** derived from trehalose.
Figure S19. ^{13}C NMR (125 MHz, D_2O) for the mixture of tetrasaccharides **4** and **5** derived from trehalose.
Figure S20. Complete assignment of ^{13}C NMR spectrum of tetrasaccharide **4** derived from trehalose.
Figure S21. Complete assignment of ^{13}C NMR spectrum of tetrasaccharide **5** derived from trehalose.
Figure S22. gCOSY and TOCSY (500 MHz, D_2O) for the mixture of tetrasaccharides **4** and **5** derived from trehalose.
Figure S23. Multiplicity-edited gHSQC and gHMBC semiselective (500 MHz, D_2O) for the mixture of tetrasaccharides **4** and **5** derived from trehalose.

Figure S24. ROESY (500 MHz, D_2O) for the mixture of tetrasaccharides **4** and **5** derived from trehalose.
Figure S25. ^1H NMR (500 MHz, D_2O) for the mixture of tetrasaccharides **6** and **7**, and pentasaccharide **8** derived from trehalose.
Figure S26. ^{13}C NMR (125 MHz, D_2O) for the mixture of tetrasaccharides **6** and **7**, and pentasaccharide **8** derived from trehalose.
Figure S27. Complete assignment of anomeric region of ^{13}C spectrum NMR of tetrasaccharides **6** and **7**, and pentasaccharide **8** derived from trehalose.
Figure S28. Complete assignment of 80-72 ppm region of ^{13}C spectrum NMR of tetrasaccharides **6** and **7**, and pentasaccharide **8** derived from trehalose.
Figure S29. Complete assignment of 72-60 ppm region of ^{13}C spectrum NMR of tetrasaccharides **6** and **7**, and pentasaccharide **8** derived from trehalose.
Figure S30. gCOSY and TOCSY (500 MHz, D_2O) for the mixture of tetrasaccharides **6** and **7**, and pentasaccharide **8** derived from trehalose.
Figure S31. Multiplicity-edited gHSQC and gHMBC semiselective (500 MHz, D_2O) for the mixture of tetrasaccharides **6** and **7**, and pentasaccharide **8** derived from trehalose.
Figure S32. ROESY (500 MHz, D_2O) for the mixture of tetrasaccharides **6** and **7**, and pentasaccharide **8** derived from trehalose.
Figure S33. ^1H NMR (500 MHz, D_2O) of trisaccharide **9** derived from lactose.
Figure S34. ^{13}C NMR (125 MHz, D_2O) of trisaccharide **9** derived from lactose.
Figure S35. Complete assignment of ^{13}C NMR spectrum of trisaccharide **9** derived from lactose.
Figure S36. gCOSY and TOCSY (500 MHz, D_2O) of **9** derived from lactose.
Figure S37. Multiplicity-edited gHSQC and gHMBC semiselective (500 MHz, D_2O) of trisaccharide **9** derived from lactose.
Figure S38. ROESY (500 MHz, D_2O) of trisaccharide **9** derived from lactose.
Figure S39. ^1H NMR (500 MHz, D_2O)) for the mixture of tetrasaccharide **10** derived from trehalose and trisaccharide **11** derived from lactose.
Figure S40. ^{13}C NMR (125 MHz, D_2O) for the mixture of tetrasaccharide **10** derived from trehalose and trisaccharide **11** derived from lactose.
Figure S41. Complete assignment of ^{13}C NMR spectrum of tetrasaccharide **10** derived from trehalose.
Figure S42. gCOSY and TOCSY (500 MHz, D_2O) for the mixture of tetrasaccharide **10** derived from trehalose. and trisaccharide **11** derived from lactose.
Figure S43. Multiplicity-edited gHSQC and gHMBC semiselective (500 MHz, D_2O) for the mixture of tetrasaccharide **10** derived from trehalose. and trisaccharide **11** derived from lactose.

Table S1. Carbohydrate Evolution during the Transgalactosylation Assay with Lactose/Trehalose and Lactose Solutions, by β-Galactosidase from *Bacillus circulans* at 50 °C, pH 4.5, Determined by GC-FID Analysis (% total carbohydrates)

		25% Lactose/25% Trehalose					25% Lactose				
**Carbohydrate**	T_r	**0 h**	**2 h**	**4 h**	**6 h**	**24 h**	**0 h**	**2 h**	**4 h**	**6 h**	**24 h**
Monosaccharides											
Galactose	11.4	0.0 ± 0.0	0.6 ± 0.1	0.7 ± 0.1	0.8 ± 0.0	1.1 ± 0.1	0.0 ± 0.0	1.8 ± 0.1	2.1 ± 0.2	2.4 ± 0.0	3.5 ± 0.3
Glucose	11.7	0.0 ± 0.0	13.4 ± 0.4	14.6 ± 0.4	15.2 ± 1.3	15.5 ± 0.6	0.0 ± 0.0	19.7 ± 0.2	21.3 ± 0.3	22.4 ± 1.3	24.7 ± 0.3
Disaccharides											
Trehalose	29.9	49.2 ± 2.7	31.8 ± 1.5	31.3 ± 1.4	30.6 ± 1.3	31.6 ± 1.0	-	-	-	-	-
Lactose	30.8	50.8 ± 2.5	15.0 ± 1.3	11.5 ± 1.2	10.2 ± 0.8	7.5 ± 0.3	100.0 ± 15.6	32.6 ± 0.0	27.6 ± 0.4	26.1 ± 1.2	19.2 ± 1.8
β-Gal-(1→4)-β-Glc	31.1	0.0 ± 0.0	0.3 ± 0.0	0.4 ± 0.0	0.5 ± 0.0	0.7 ± 0.3	0.0 ± 0.0	1.1 ± 0.0	1.4 ± 0.1	1.6 ± 0.1	2.4 ± 0.2
β-Gal-(1→3)-β-Glc	31.7	0.0 ± 0.0	2.8 ± 0.1	4.0 ± 0.1	4.4 ± 0.1	5.3 ± 0.1	0.0 ± 0.0	7.6 ± 0.2	10.1 ± 0.1	11.5 ± 0.5	14.7 ± 0.2
β-Gal-(1→2)-β-Glc	31.9	0.0 ± 0.0	1.1 ± 0.0	1.6 ± 0.0	1.8 ± 0.0	2.4 ± 0.1	0.0 ± 0.0	2.3 ± 0.2	3.3 ± 0.1	3.8 ± 0.3	5.8 ± 0.3
β-Gal-(1→6)-β-Glc	33.8	0.0 ± 0.0	0.4 ± 0.0	0.6 ± 0.0	0.7 ± 0.0	1.0 ± 0.0	0.0 ± 0.0	0.9 ± 0.1	1.1 ± 0.1	1.4 ± 0.1	2.5 ± 0.1
Trisaccharides											
β-Gal-(1→4)-Tre*	46.0	0.0 ± 0.0	18.3 ± 0.6	18.4 ± 0.8	18.7 ± 1.6	17.3 ± 0.5	-	-	-	-	-
β-Gal-(1→4)-Lac	47.6	0.0 ± 0.0	5.2 ± 0.5	4.1 ± 0.3	3.8 ± 0.3	3.0 ± 0.3	0.0 ± 0.0	21.1 ± 0.3	18.4 ± 0.7	16.4 ± 0.6	12.8 ± 1.5
β-Gal-(1→6)-Lac	48.0	0.0 ± 0.0	1.0 ± 0.0	1.4 ± 0.1	1.5 ± 0.1	1.9 ± 0.1	0.0 ± 0.0	4.3 ± 0.3	6.0 ± 0.4	6.3 ± 0.2	8.2 ± 0.6
β-Gal-(1→6)-Tre*	49.0	0.0 ± 0.0	1.5 ± 0.1	2.4 ± 0.1	2.9 ± 0.3	3.8 ± 0.3	-	-	-	-	-
Other trisaccharides	-	0.0 ± 0.0	0.6 ± 0.1	0.7 ± 0.0	0.8 ± 0.1	0.9 ± 0.1	-	-	-	-	-
Tetrasaccharides											
β-Gal-(1→4)-β-Gal-(1→6)-Tre* + β-Gal-(1→4)-α-Glc-(1↔1)-[β-Gal-(1→4)]-α-Glc*	58.9	0.0 ± 0.0	2.0 ± 0.2	2.1 ± 0.2	2.1 ± 0.2	1.9 ± 0.2	-	-	-	-	-
β-Gal-(1→4)-β-Gal-(1→4)-Tre*	59.5	0.0 ± 0.0	3.7 ± 0.2	4.0 ± 0.3	4.0 ± 0.4	3.5 ± 0.2	-	-	-	-	-
β-Gal-(1→4)-α-Glc-(1↔1)-[β-Gal-(1→6)]-α-Glc* + β-Gal-(1→6)-α-Glc-(1↔1)-[β-Gal-(1→6)]-α-Glc*	60.6	0.0 ± 0.0	1.9 ± 0.2	2.0 ± 0.2	2.1 ± 0.1	2.3 ± 0.1	-	-	-	-	-
Other tetrasaccharides	-	-	-	-	-	-	0.0 ± 0.0	9.4 ± 0.3	9.0 ± 0.2	8.2 ± 0.7	6.5 ± 1.0
Pentasaccharides											
β-Gal-(1→4)-*β*-Gal-(1→4)-*β*-Gal-(1→4)-Tre*	70.0	0.0 ± 0.0	0.9 ± 0.0	1.3 ± 0.1	1.3 ± 0.1	1.4 ± 0.1	-	-	-	-	-

Data are expressed as the mean ± SD (*n* = 4). Tr: Retention time.
* Trehalose derivatives synthesized.

Table S2. Carbohydrate Evolution during the Transgalactosylation Assay with Lactose/Trehalose and Lactose Solutions, by β-Galactosidase from *Aspergillus oryzae* at 50 °C, pH 4.5, Determined by GC-FID Analysis (% total carbohydrates)

Carbohydrate	T_r	25% Lactose/25% Trehalose 0 h	2 h	4 h	6 h	24 h	25% Lactose 0 h	2 h	4 h	6 h	24 h
Monosaccharides											
Galactose	11.4	0.0 ± 0.0	6.0 ± 0.3	9.1 ± 0.7	12.3 ± 1.2	20.4 ± 0.5	0.0 ± 0.0	11.7 ± 0.3	18.5 ± 1.0	23.7 ± 4.1	43.5 ± 0.2
Glucose	11.7	0.0 ± 0.0	12.2 ± 0.6	15.7 ± 1.1	19.2 ± 1.9	23.0 ± 2.0	0.0 ± 0.0	23.7 ± 0.3	30.5 ± 1.3	34.6 ± 6.1	47.2 ± 0.1
Disaccharides											
Trehalose	29.9	49.5 ± 2.8	45.7 ± 2.2	44.8 ± 3.3	44.5 ± 0.5	44.3 ± 0.7	-	-	-	-	-
Lactose	30.8	50.5 ± 1.4	20.5 ± 0.9	13.7 ± 1.0	10.3 ± 1.3	1.3 ± 0.2	100.0 ± 2.1	36.8 ± 0.1	23.8 ± 0.4	16.4 ± 3.1	1.6 ± 0.2
β-Gal-(1→4)-Gal	31.1	0.0 ± 0.0	0.2 ± 0.0	0.4 ± 0.1	0.4 ± 0.0	0.3 ± 0.0	0.0 ± 0.0	0.6 ± 0.1	0.8 ± 0.0	0.9 ± 0.2	0.6 ± 0.0
β-Gal-(1→6)-Glc	33.7	0.0 ± 0.0	2.0 ± 0.1	3.1 ± 0.3	3.5 ± 0.4	2.2 ± 0.2	0.0 ± 0.0	4.8 ± 0.3	7.1 ± 0.3	8.1 ± 1.6	4.3 ± 0.4
β-Gal-(1→6)-Gal	34.4	0.0 ± 0.0	0.5 ± 0.0	0.7 ± 0.0	0.9 ± 0.0	0.6 ± 0.0	0.0 ± 0.0	1.2 ± 0.1	1.7 ± 0.1	1.9 ± 0.3	1.0 ± 0.1
Other disaccharides	-	0.0 ± 0.0	1.0 ± 0.0	1.4 ± 0.1	1.5 ± 0.2	1.0 ± 0.1	0.0 ± 0.0	2.3 ± 0.1	2.9 ± 0.0	3.2 ± 0.6	1.8 ± 0.1
Trisaccharides											
[*β*-Gal-(1→4)]-[*β*-Gal-(1→6)]-Glc	47.4	0.0 ± 0.0	1.5 ± 0.1	1.4 ± 0.1	1.2 ± 0.1	0.7 ± 0.0	0.0 ± 0.0	3.4 ± 0.1	3.5 ± 0.2	3.3 ± 0.6	0.0 ± 0.0
β-Gal-(1→6)-Lac	48.0	0.0 ± 0.0	4.1 ± 0.3	2.4 ± 0.2	1.3 ± 0.2	0.4 ± 0.0	0.0 ± 0.0	4.5 ± 0.3	3.1 ± 0.2	2.2 ± 0.3	0.0 ± 0.0
β-Gal-(1→6)-Tre*	49.0	0.0 ± 0.0	2.9 ± 0.2	4.0 ± 0.3	5.0 ± 0.5	4.8 ± 0.1	-	-	-	-	-
Other trisaccharides	-	0.0 ± 0.0	2.6 ± 0.3	2.9 ± 0.2	2.4 ± 0.3	1.3 ± 0.	0.0 ± 0.0	8.4 ± 0.2	6.2 ± 0.2	4.5 ± 0.4	0.0 ± 0.0
Tetrasaccharides											
β-Gal-(1→6)-*β*-Gal-(1→6)-Tre*	61.2	0.0 ± 0.0	1.1 ± 0.3	1.2 ± 0.3	1.4 ± 0.1	0.8 ± 0.0	-	-	-	-	-
Other tetrasaccharides	-	-	-	-	-	-	0.0 ± 0.0	2.8 ± 0.2	1.8 ± 0.2	1.2 ± 0.3	0.0 ± 0.0

Data are expressed as the mean ± SD (*n* = 4). Tr: Retention time.
* Trehalose derivatives synthesized.

NMR spectra:

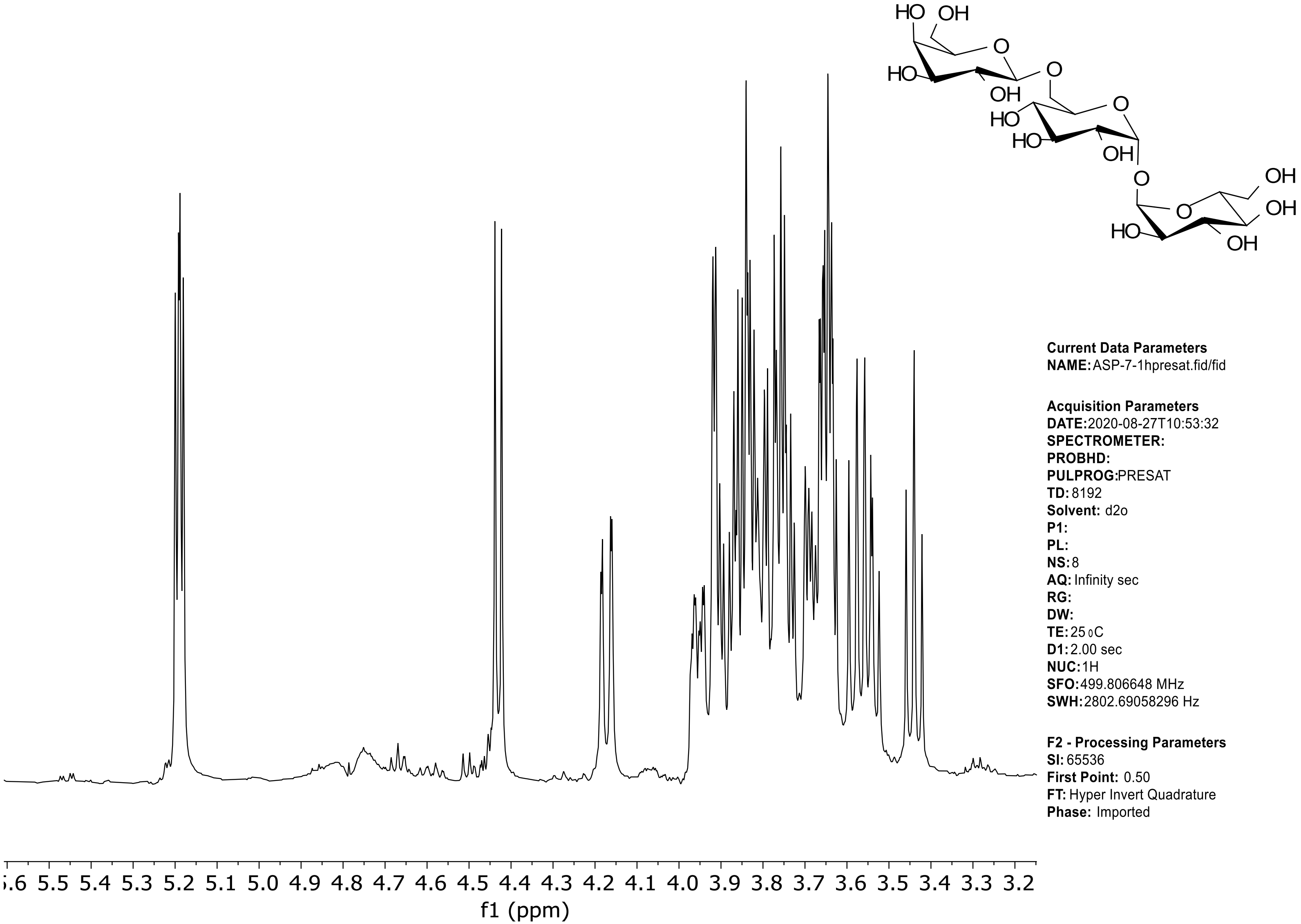

Figure S1. ^{1}H NMR (500 MHz, D_2O) of trisaccharide **1** derived from trehalose.

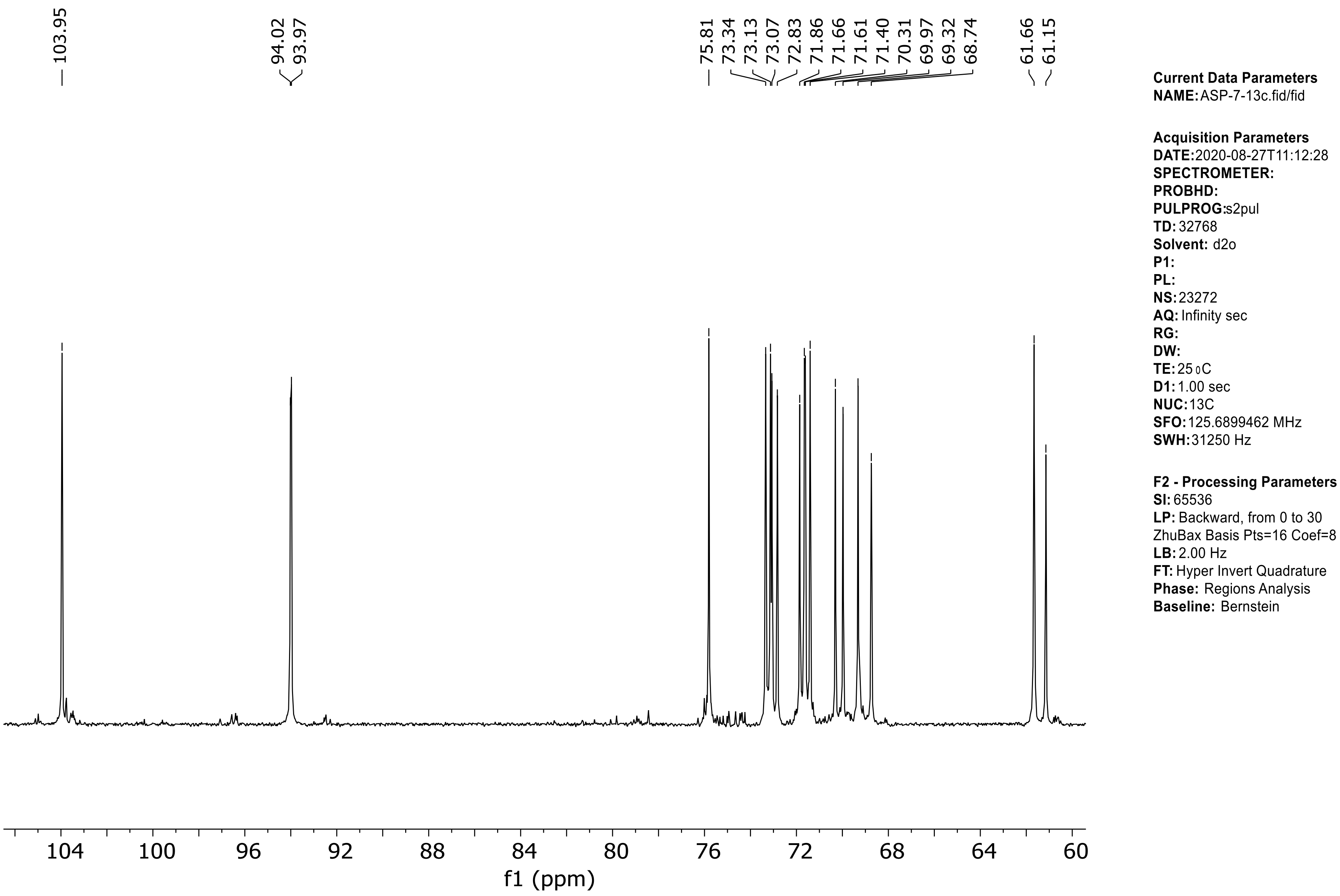

Figure S2. ^{13}C NMR (125 MHz, D_2O) of trisaccharide **1** derived from trehalose.

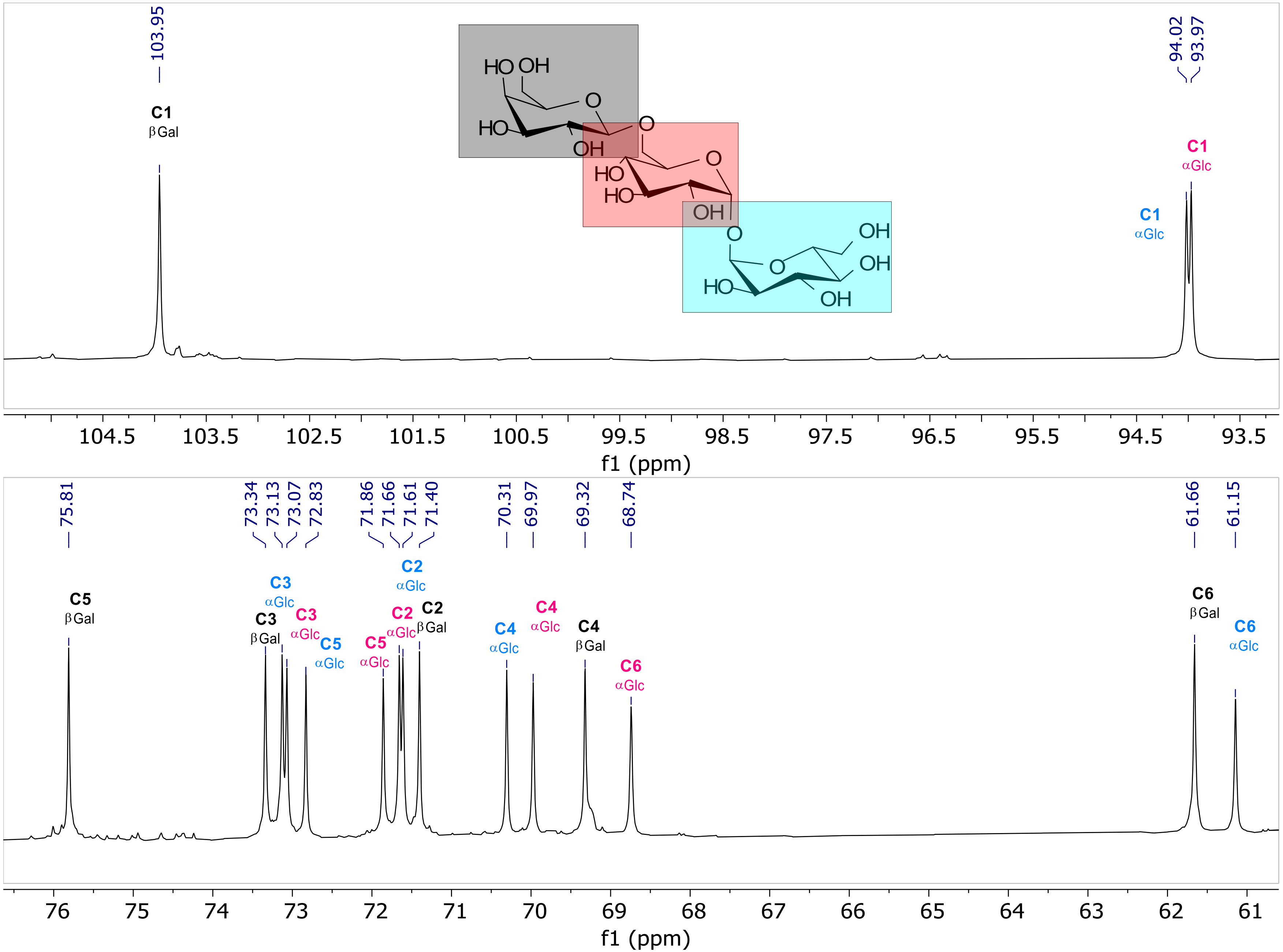

Figure S3. Complete assignment of ^{13}C NMR spectrum of trisaccharide **1** derived from trehalose.

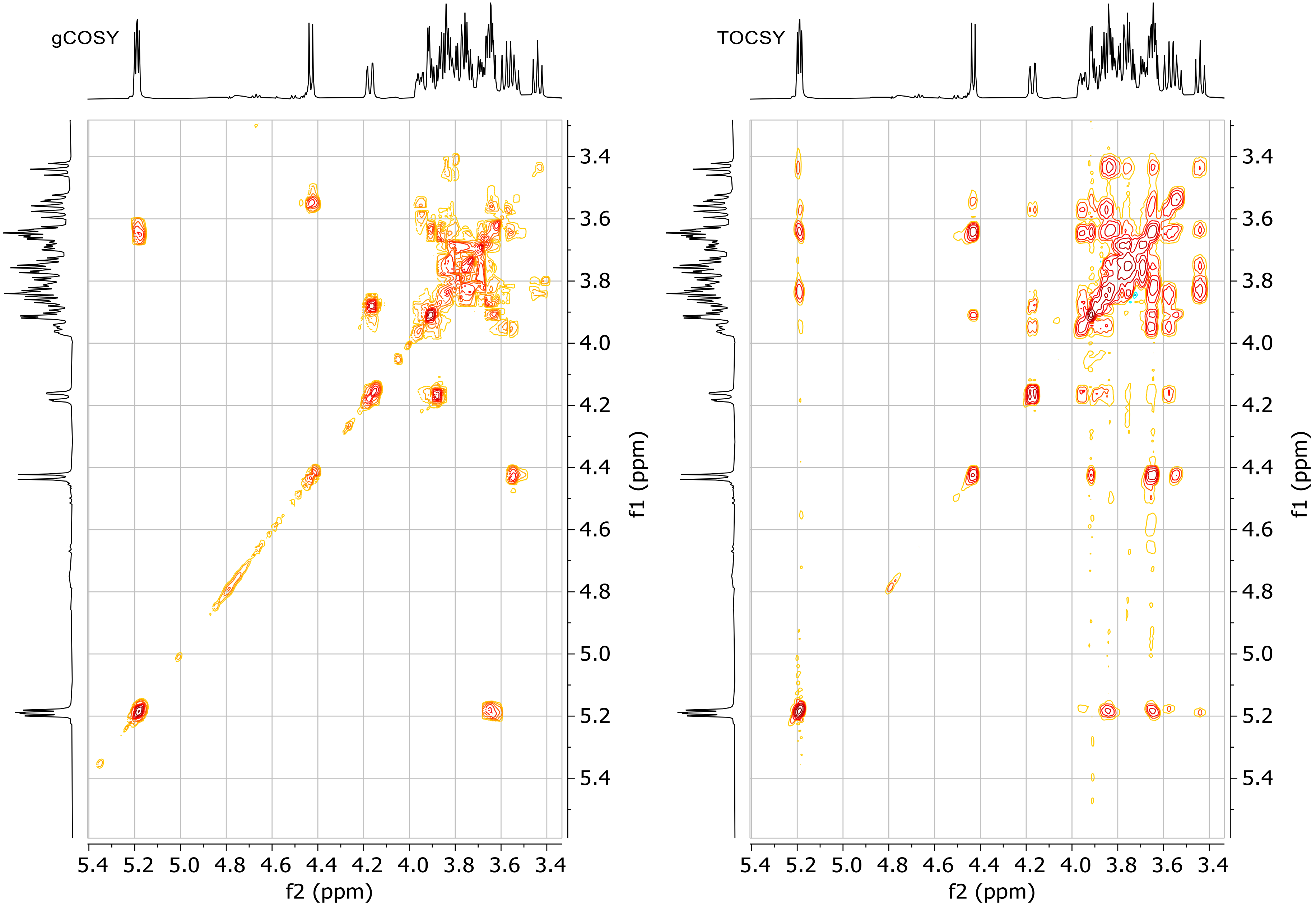

Figure S4. gCOSY and TOCSY (500 MHz, D_2O) of trisaccharide **1** derived from trehalose.

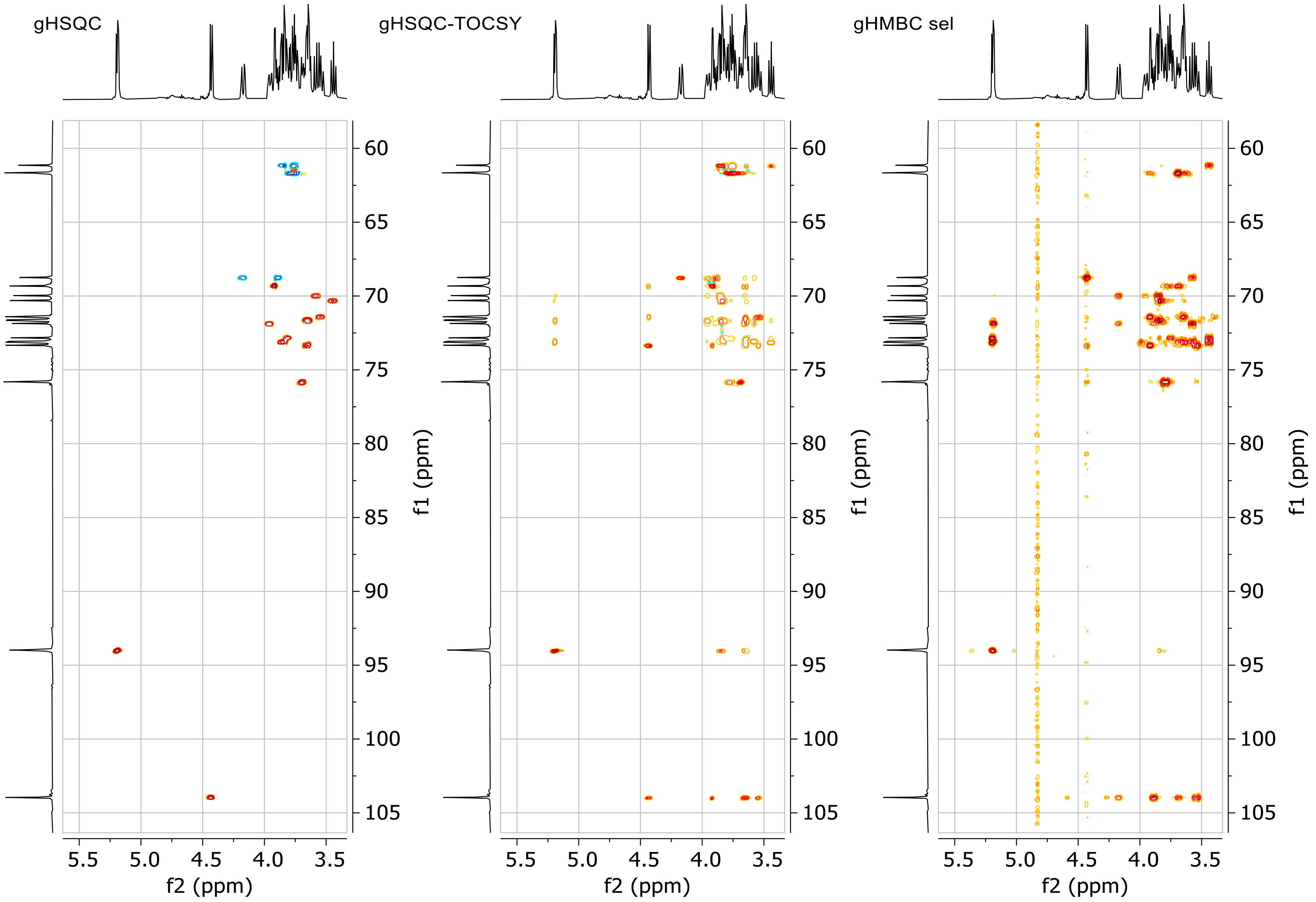

Figure S5. Multiplicity-edited gHSQC (methylene: blue cross peaks; methine: red cross peaks), gHSQC-TOCSY and gHMBC semiselective (500 MHz, D_2O) of trisaccharide **1** derived from trehalose.

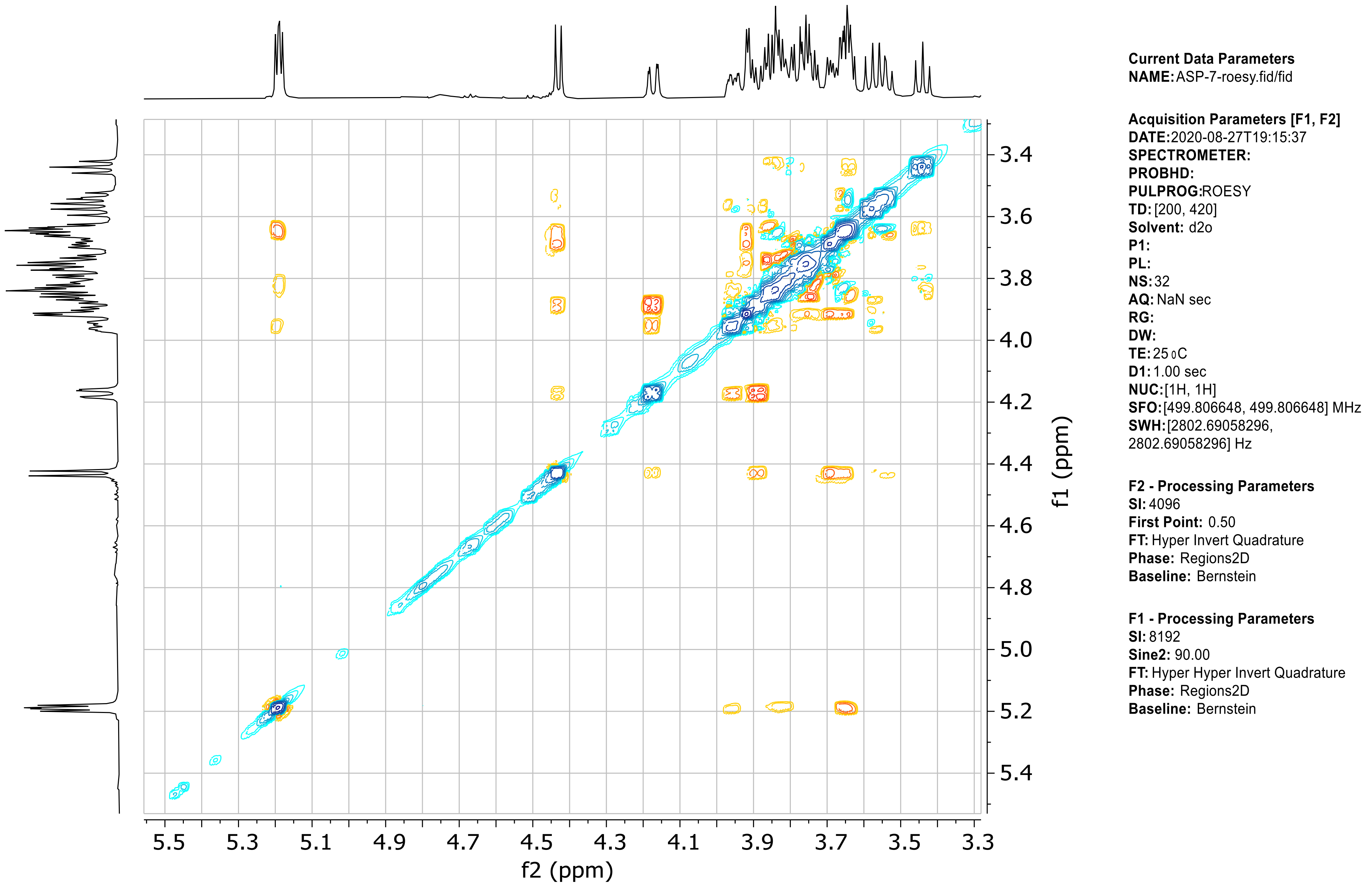

Figure S6. ROESY (500 MHz, D_2O) of trisaccharide **1** derived from trehalose.

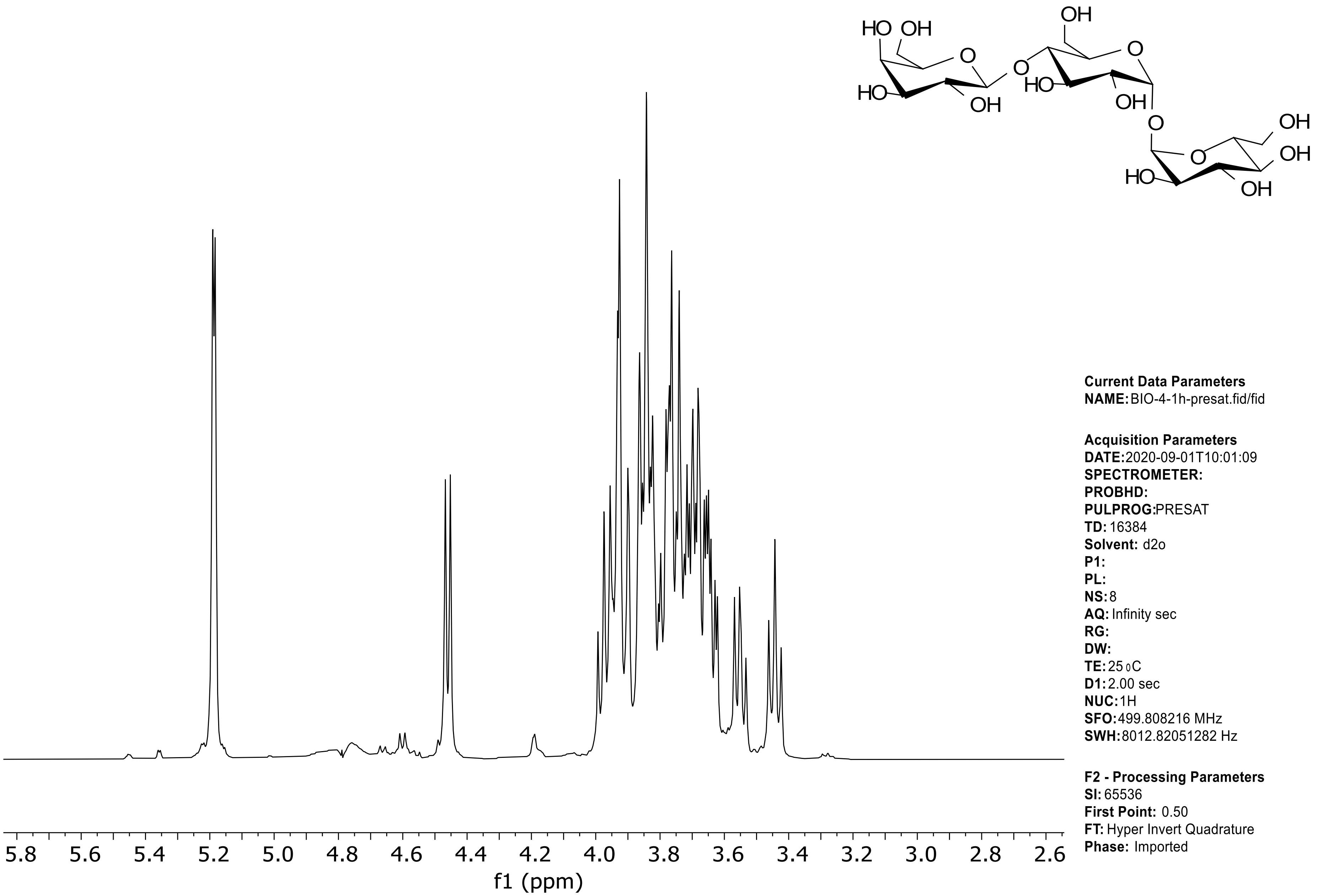

Figure S7. ^{1}H NMR (500 MHz, D_2O) of trisaccharide **2** derived from trehalose.

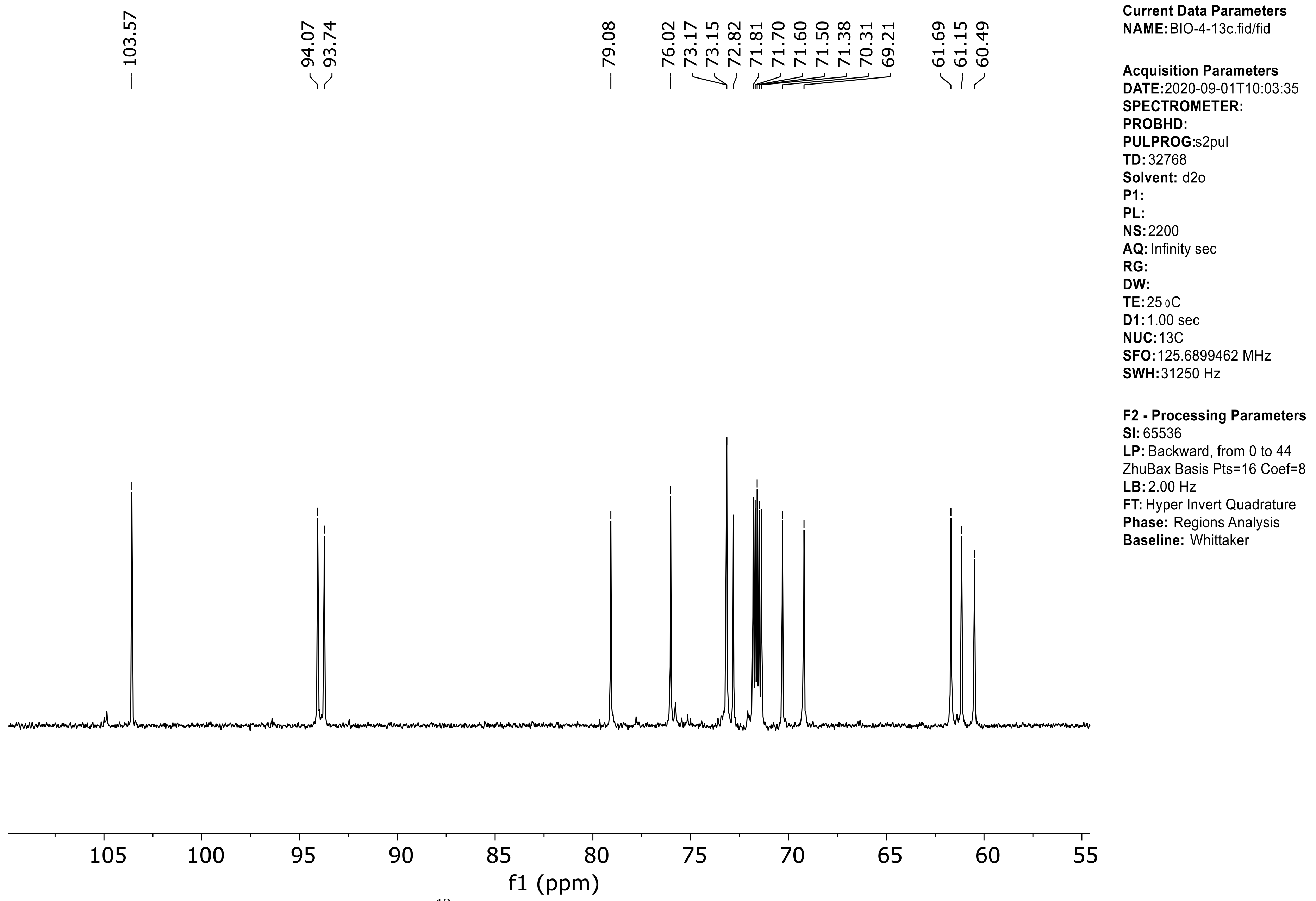

Figure S8. ^{13}C NMR (125 MHz, D_2O) of trisaccharide **2** derived from trehalose.

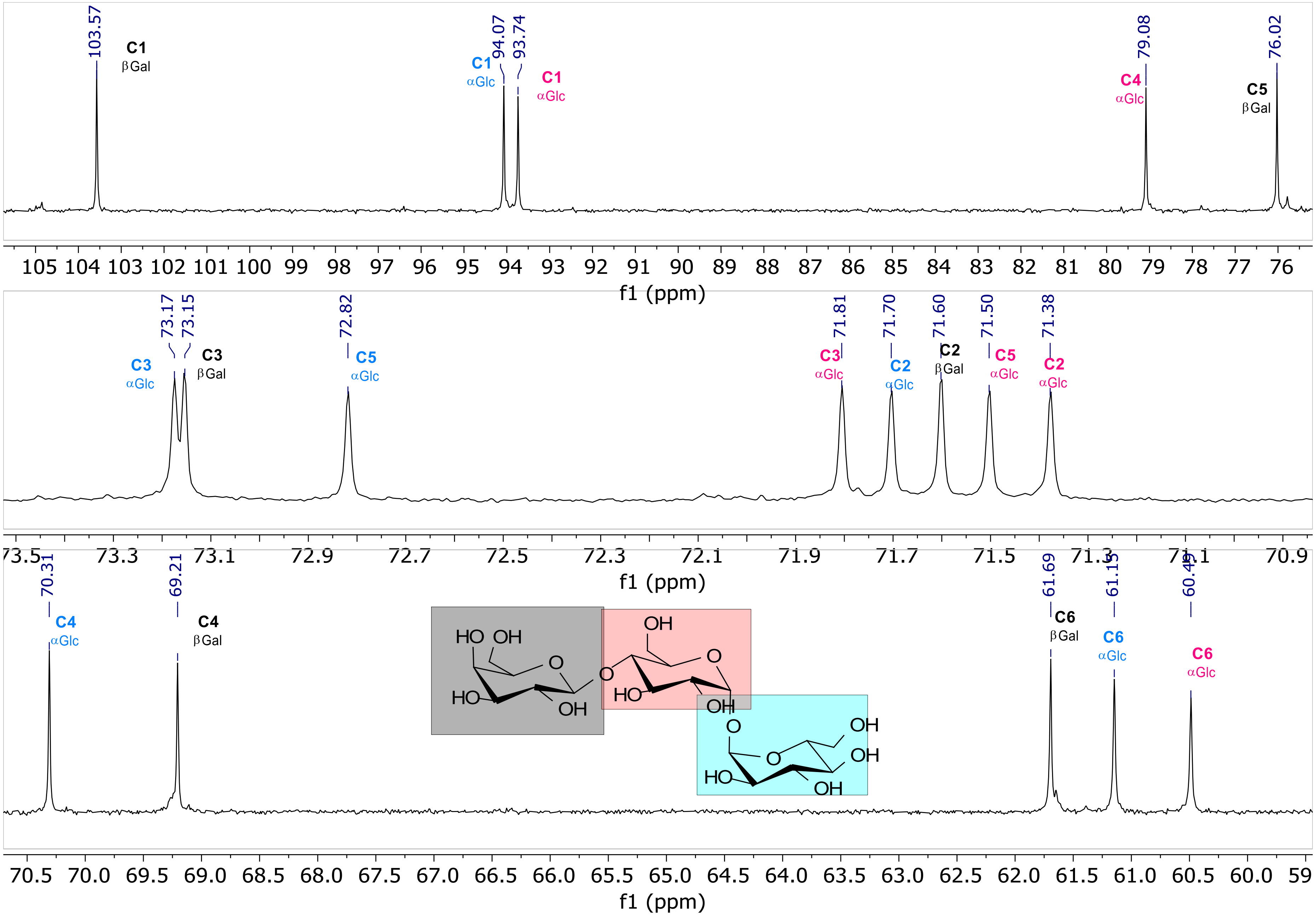

Figure S9. Complete assignment of ^{13}C NMR spectrum of trisaccharide **2** derived from trehalose.

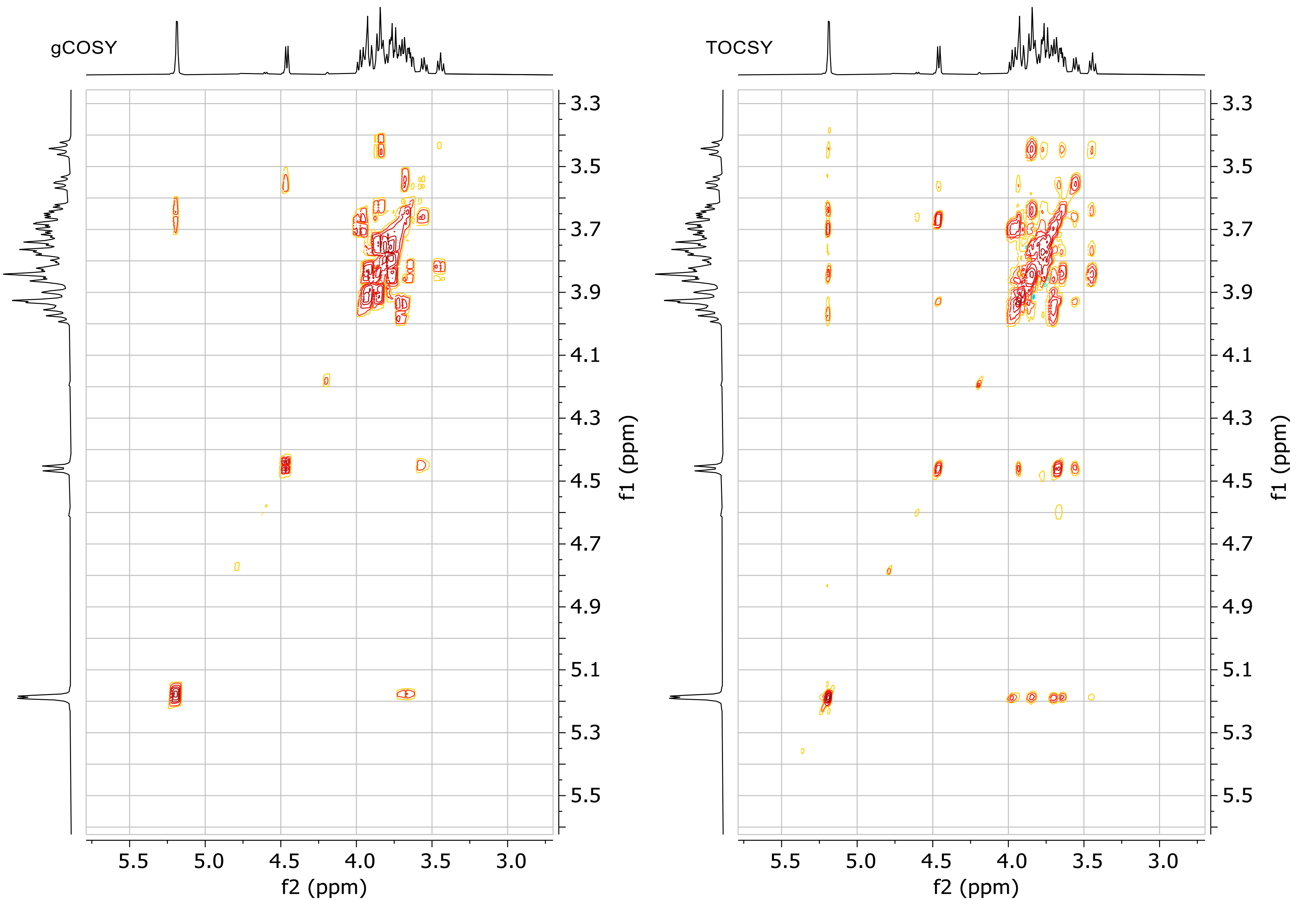

Figure S10. gCOSY and TOCSY (500 MHz, D_2O) of trisaccharide **2** derived from trehalose.

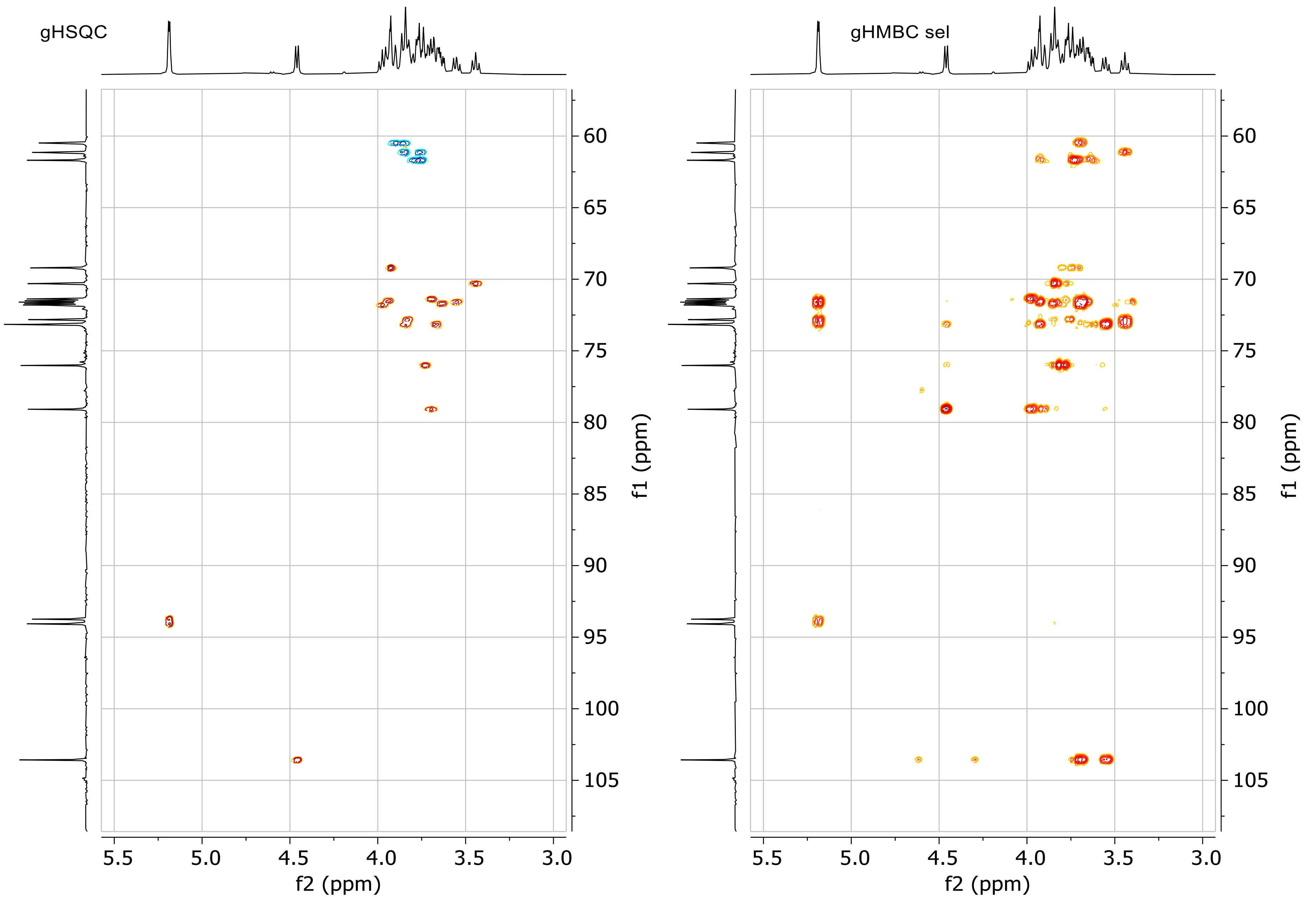

Figure S11. Multiplicity-edited gHSQC (methylene: blue cross peaks; methine: red cross peaks), and gHMBC semiselective (500 MHz, D_2O) of trisaccharide **2** derived from trehalose.

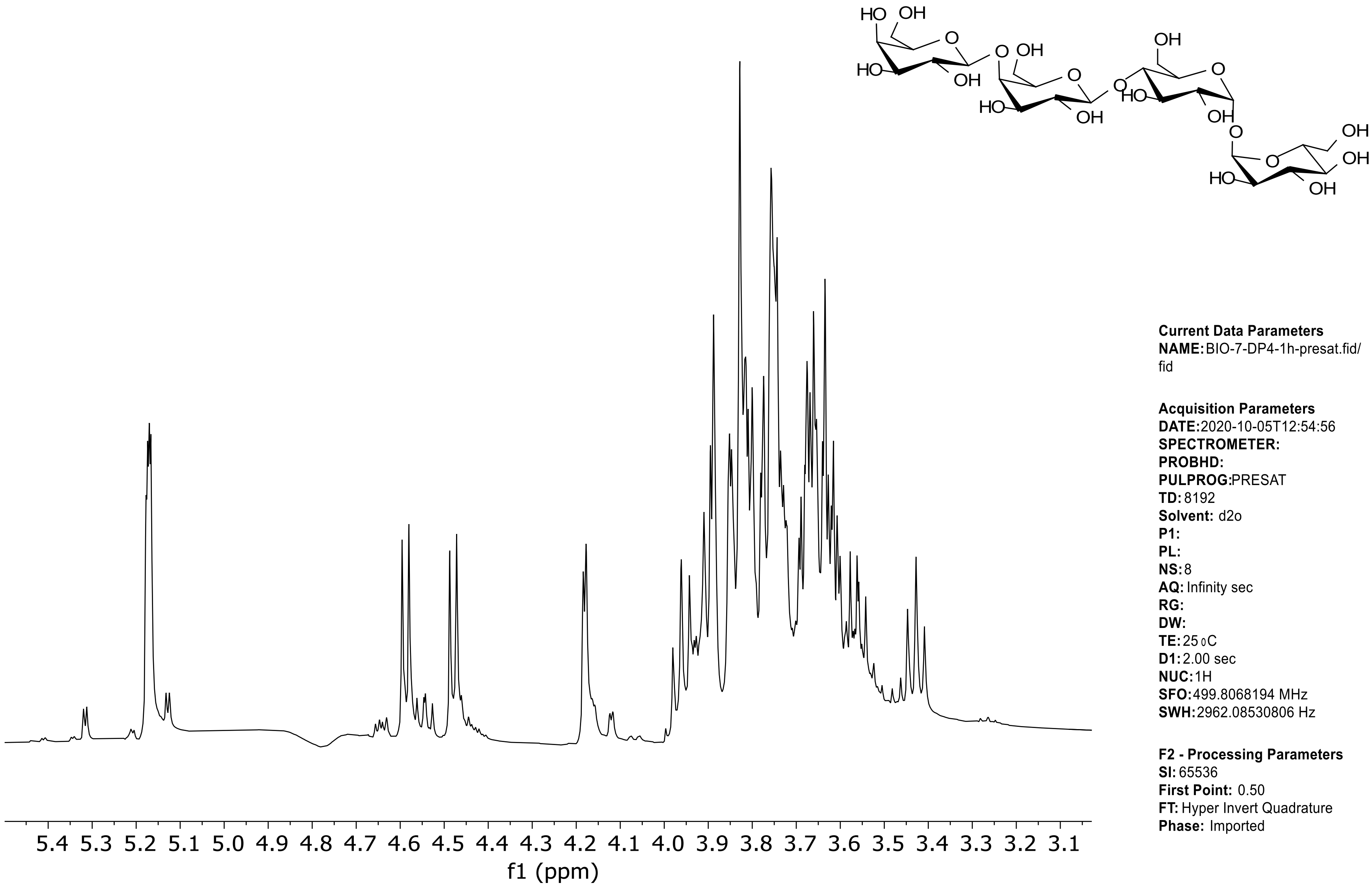

Figure S12. ^{1}H NMR (500 MHz, D_2O) of tetrasaccharide **3** derived from trehalose.

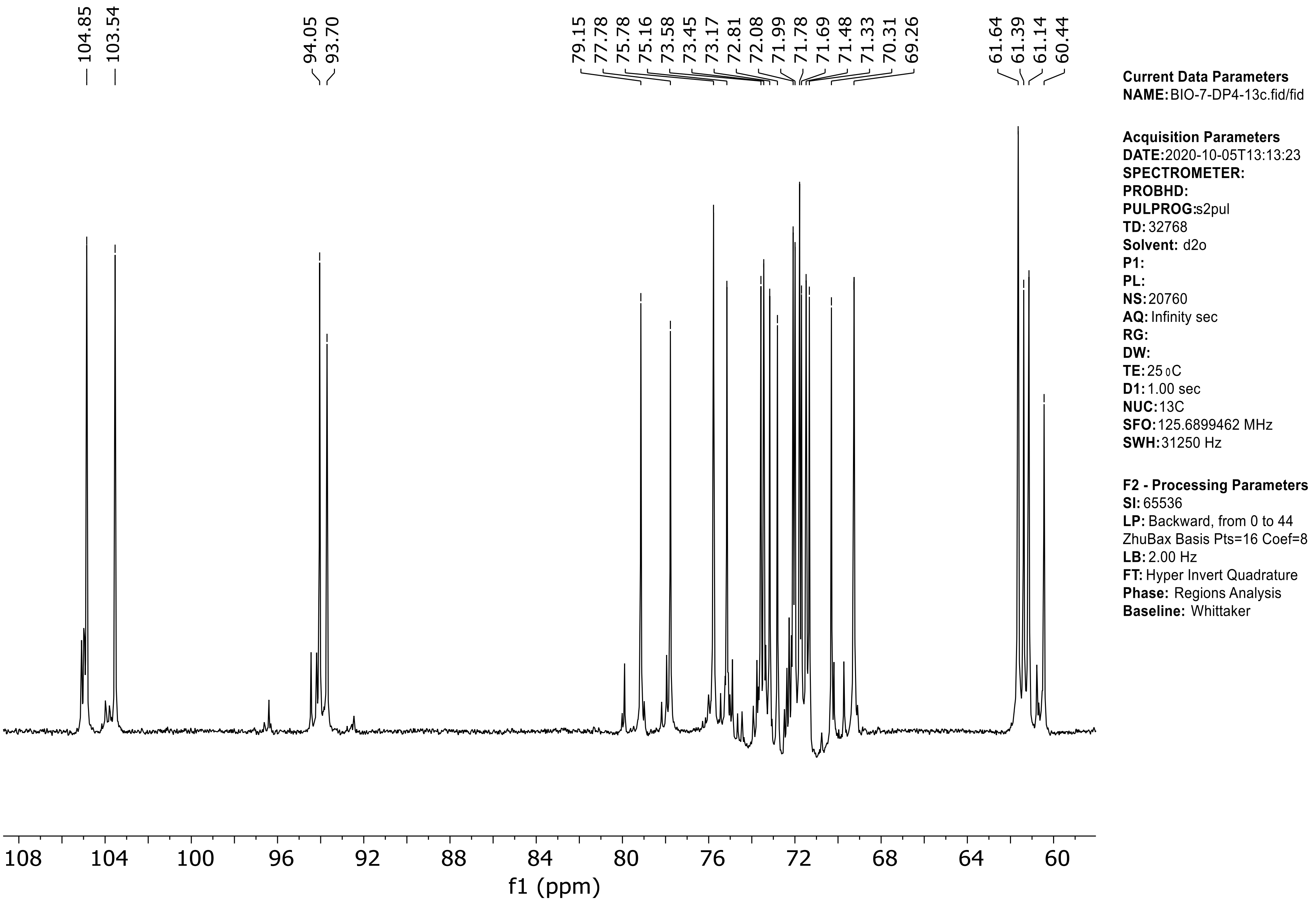

Figure S13. ^{13}C NMR (125 MHz, D_2O) of tetrasaccharide **3** derived from trehalose.

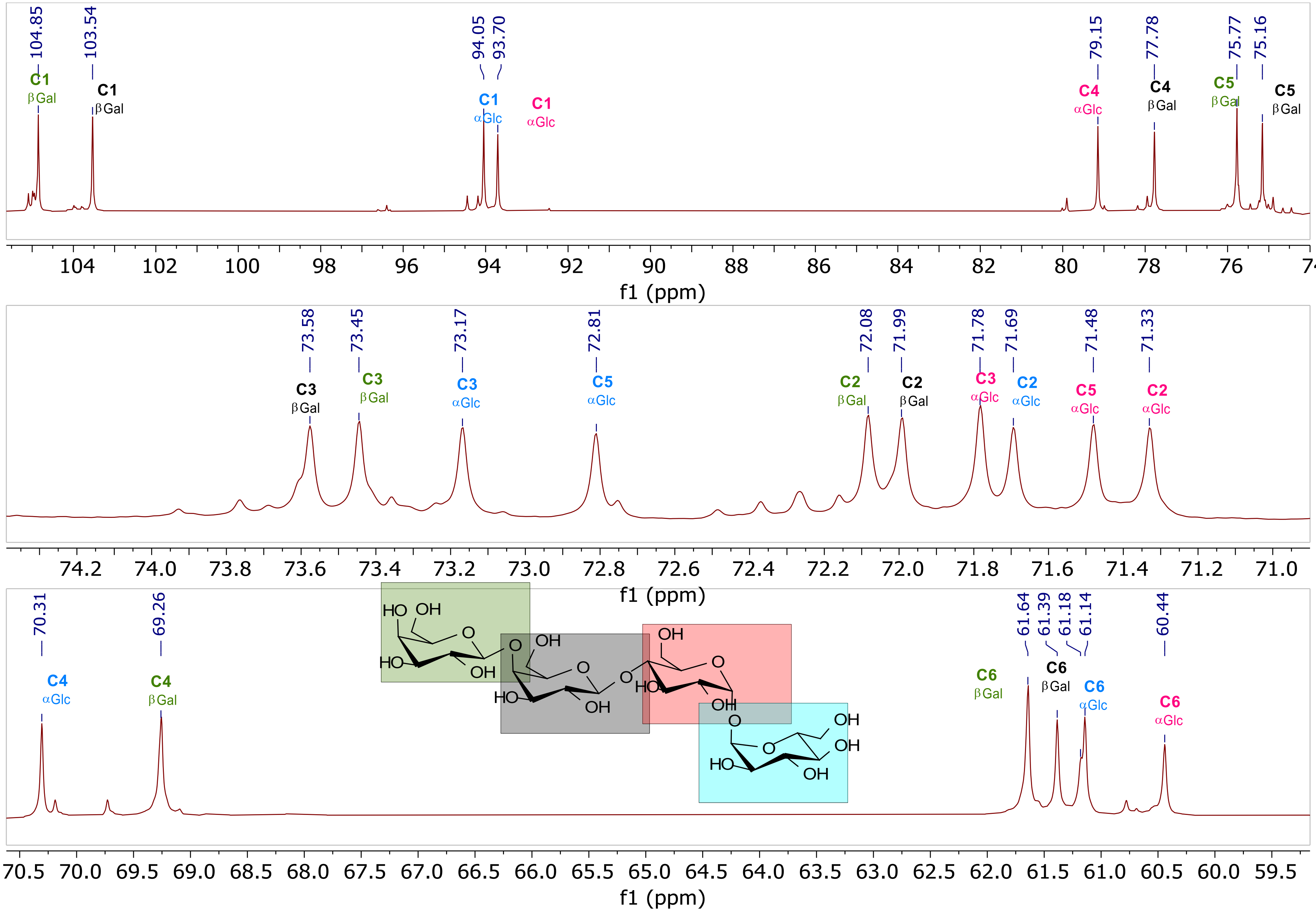

Figure S14. Complete assignment of ^{13}C NMR spectrum of tetrasaccharide **3** derived from trehalose.

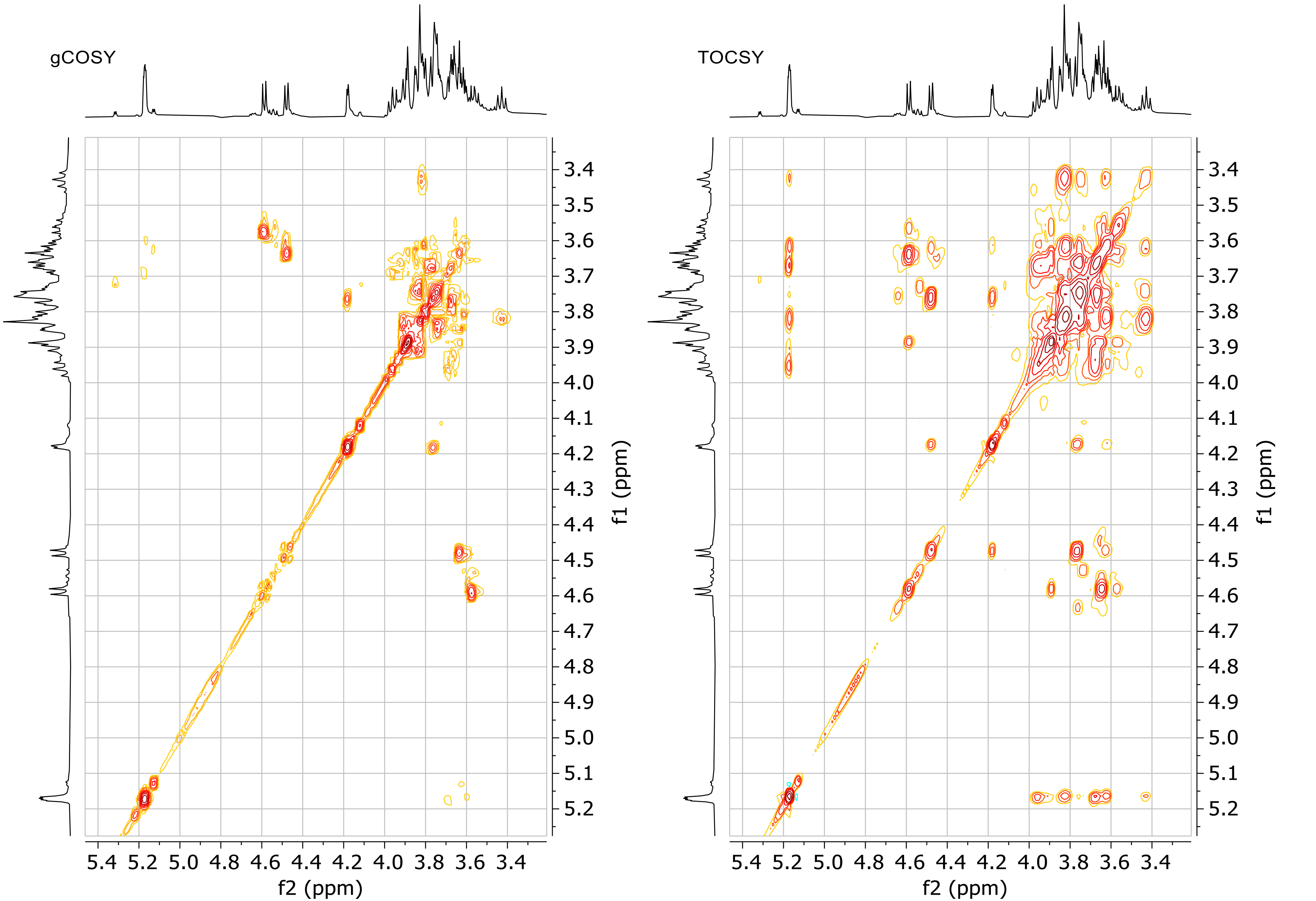

Figure S15. gCOSY and TOCSY (500 MHz, D_2O) of tetrasaccharide **3** derived from trehalose.

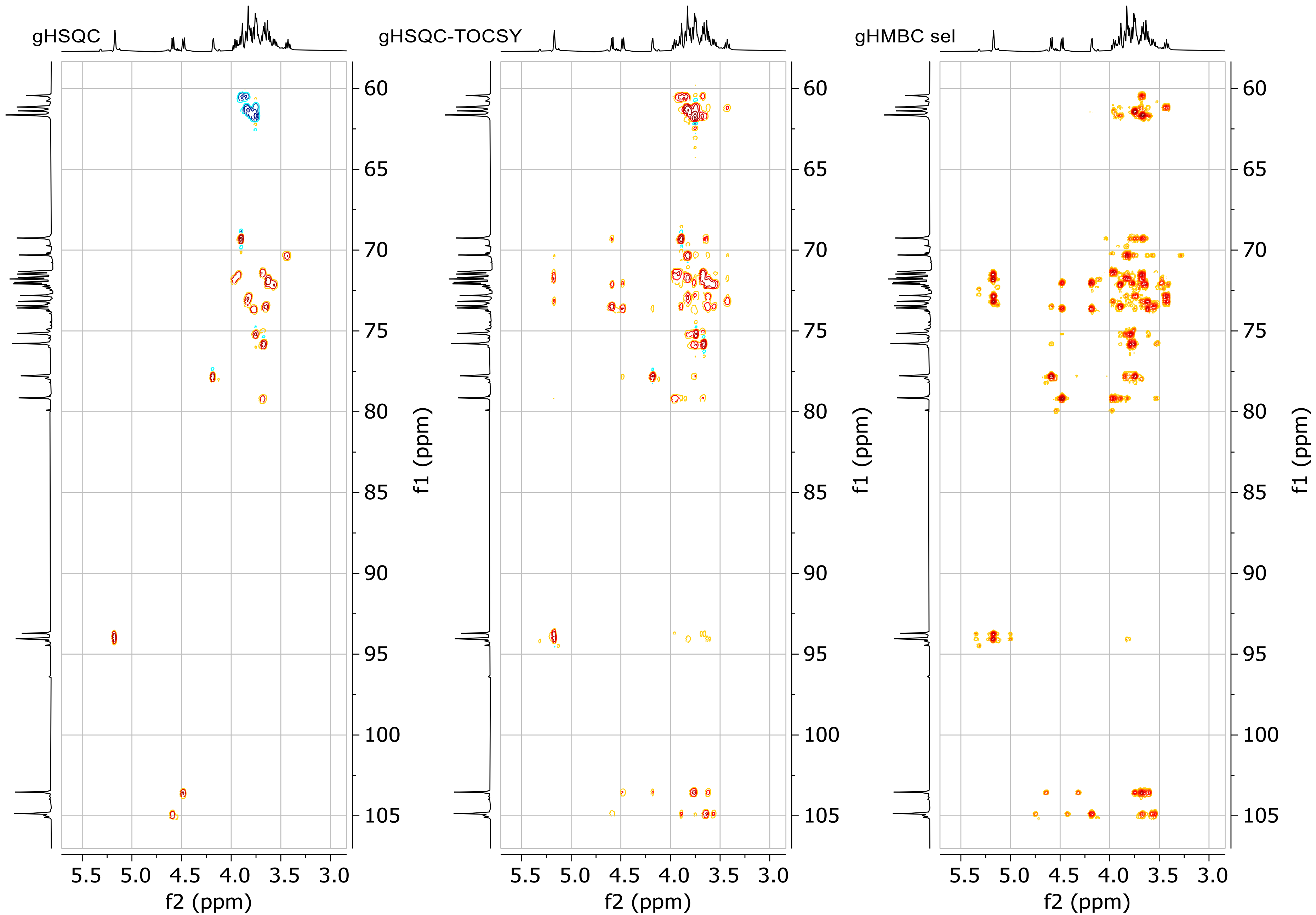

Figure S16. Multiplicity-edited gHSQC (methylene: blue cross peaks; methine: red cross peaks), gHSQC-TOCSY and gHMBC semiselective (500 MHz, D_2O) of tetrasaccharide **3** derived from trehalose.

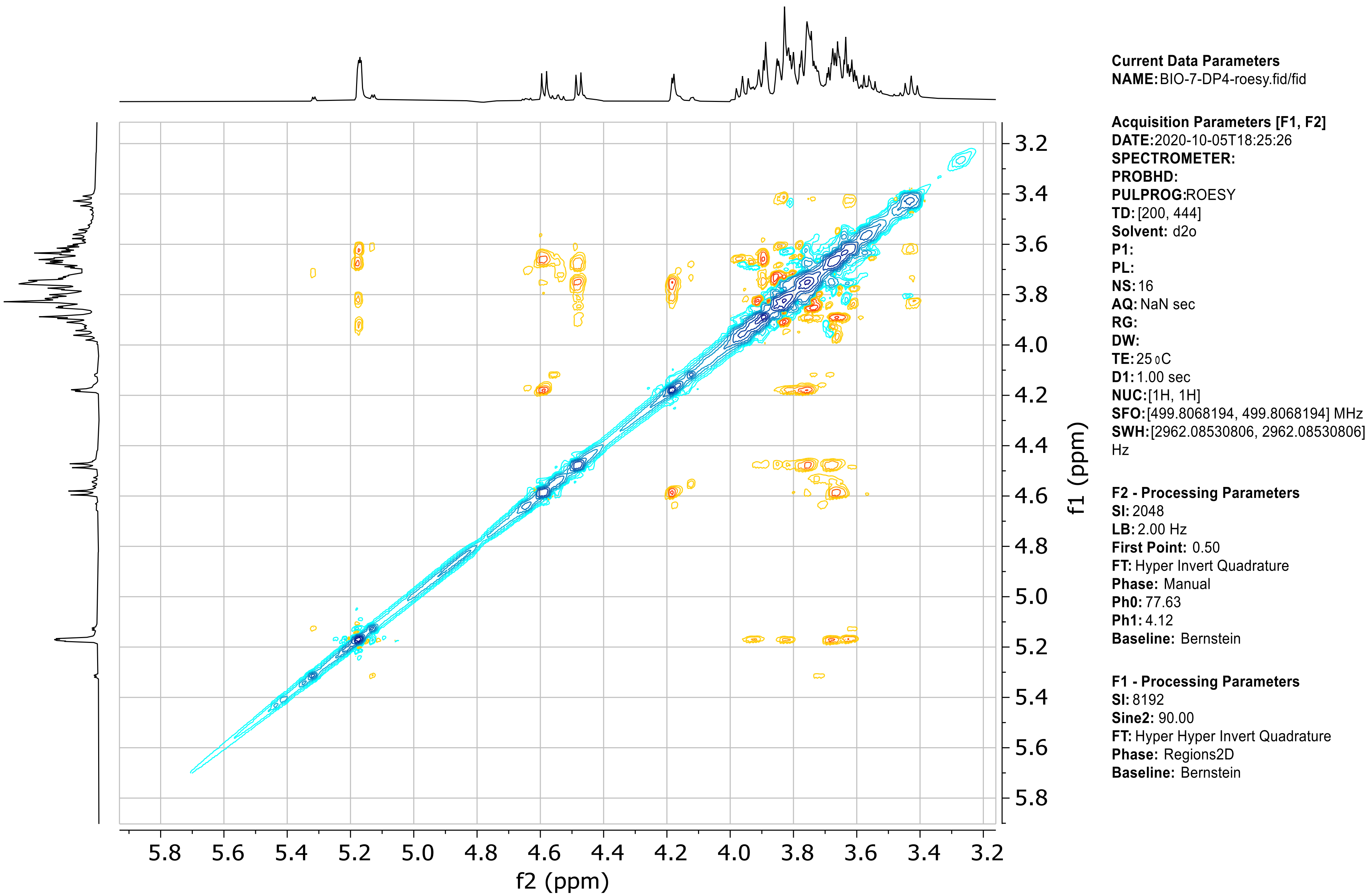

Figure S17. ROESY (500 MHz, D_2O) of tetrasaccharide **3** derived from trehalose.

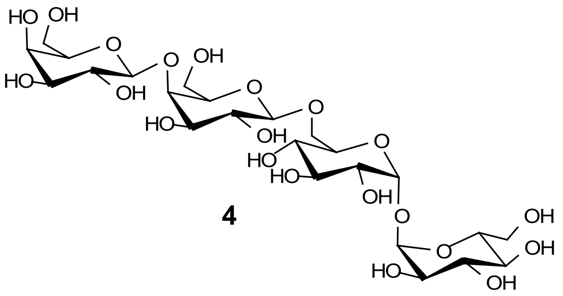

4

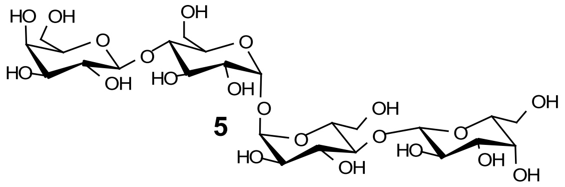

5

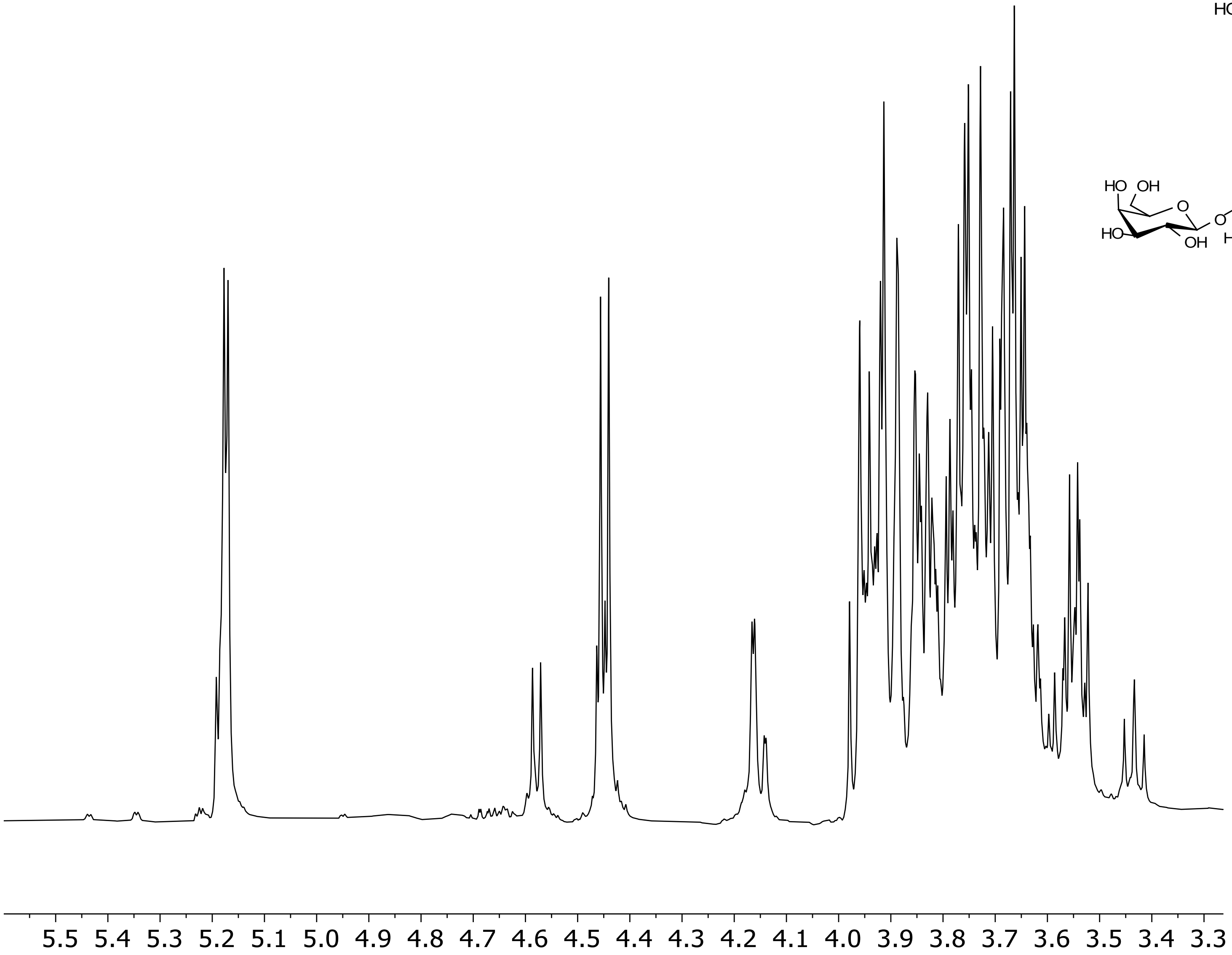

Current Data Parameters
NAME: BIO-8-DP4-1h-presat.fid/fid

Acquisition Parameters
DATE: 2020-10-19T13:07:15
SPECTROMETER:
PROBHD:
PULPROG: PRESAT
TD: 8192
Solvent: d2o
P1:
PL:
NS: 8
AQ: Infinity sec
RG:
DW:
TE: 25 0C
D1: 2.00 sec
NUC: 1H
SFO: 499.8067877 MHz
SWH: 2920.56074766 Hz

F2 - Processing Parameters
SI: 65536
First Point: 0.50
FT: Hyper Invert Quadrature
Phase: Imported

Figure S18. ^{1}H NMR (500 MHz, D_2O) for the mixture of tetrasaccharides **4** and **5** derived from trehalose.

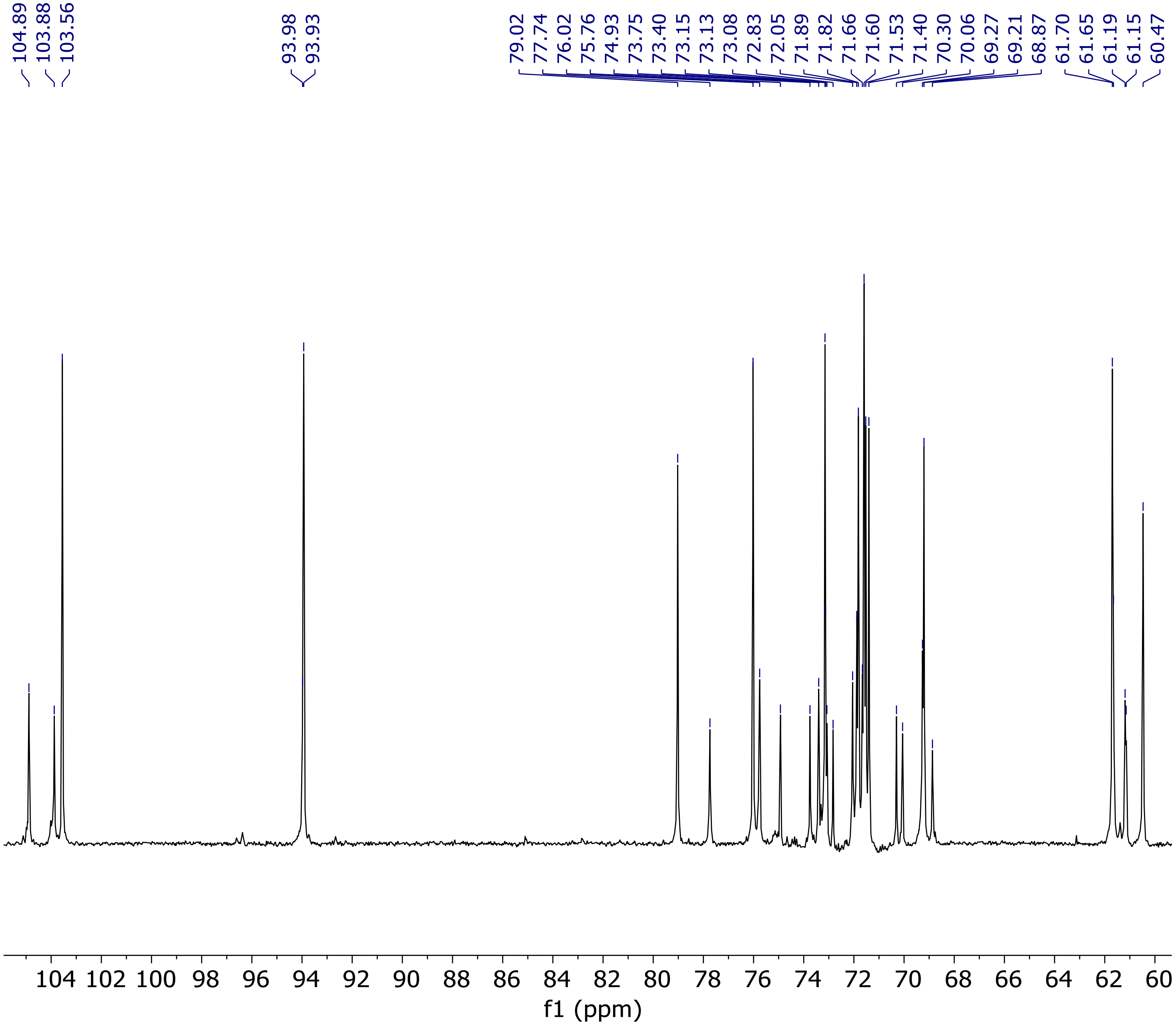

Current Data Parameters
NAME: BIO-8-DP4-13c.fid/fid

Acquisition Parameters
DATE: 2020-10-19T13:18:00
SPECTROMETER:
PROBHD:
PULPROG: s2pul
TD: 32768
Solvent: d2o
P1:
PL:
NS: 21500
AQ: Infinity sec
RG:
DW:
TE: 25 0C
D1: 1.00 sec
NUC: 13C
SFO: 125.6899462 MHz
SWH: 31250 Hz

F2 - Processing Parameters
SI: 65536
LP: Backward, from 0 to 44
ZhuBax Basis Pts=16 Coef=8
LB: 2.00 Hz
FT: Hyper Invert Quadrature
Phase: Regions Analysis
Baseline: Whittaker

Figure S19. ^{13}C NMR (125 MHz, D_2O) for the mixture of tetrasaccharides **4** and **5** derived from trehalose.

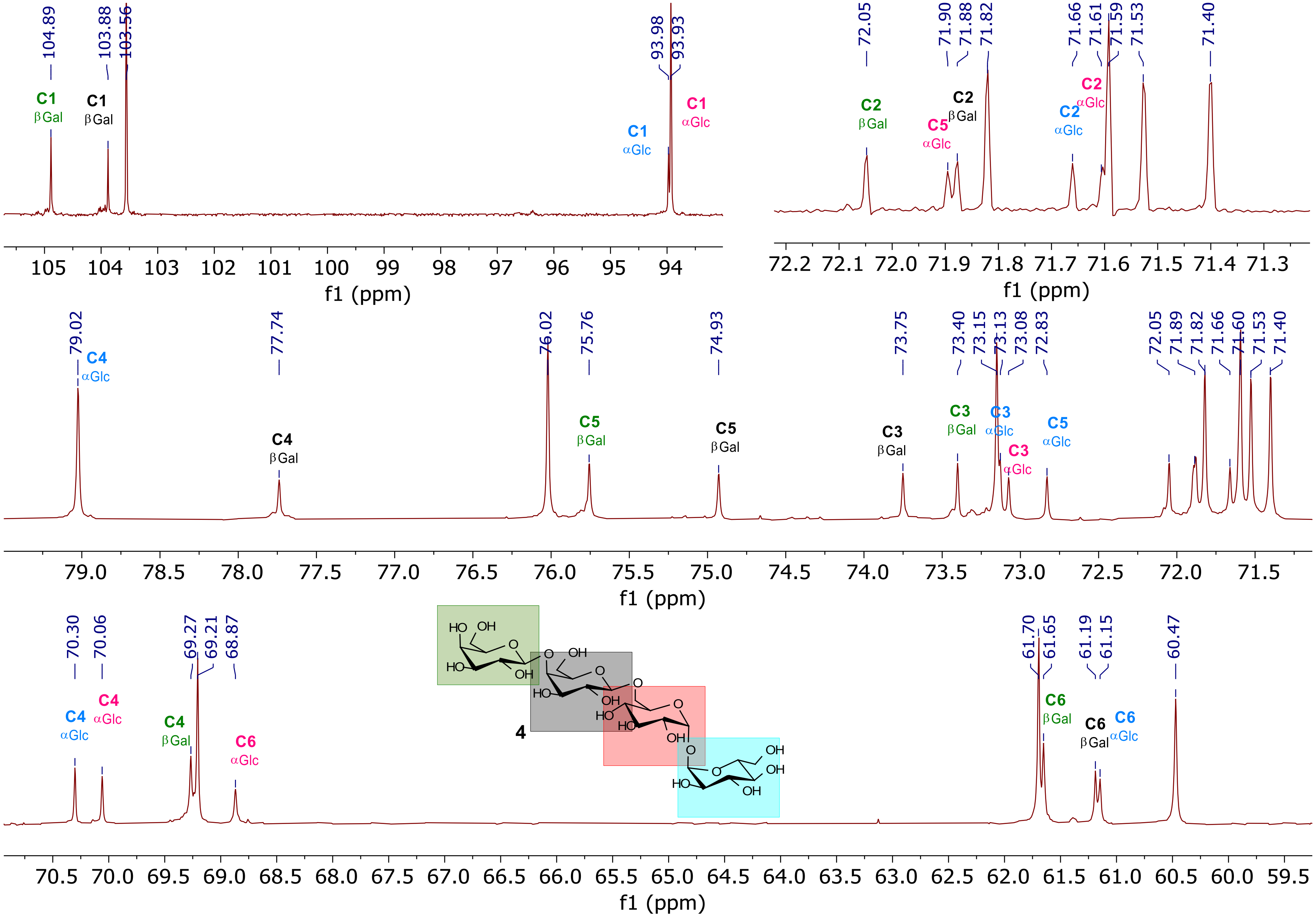

Figure S20. Complete assignment of ^{13}C NMR spectrum of tetrasaccharide **4** derived from trehalose.

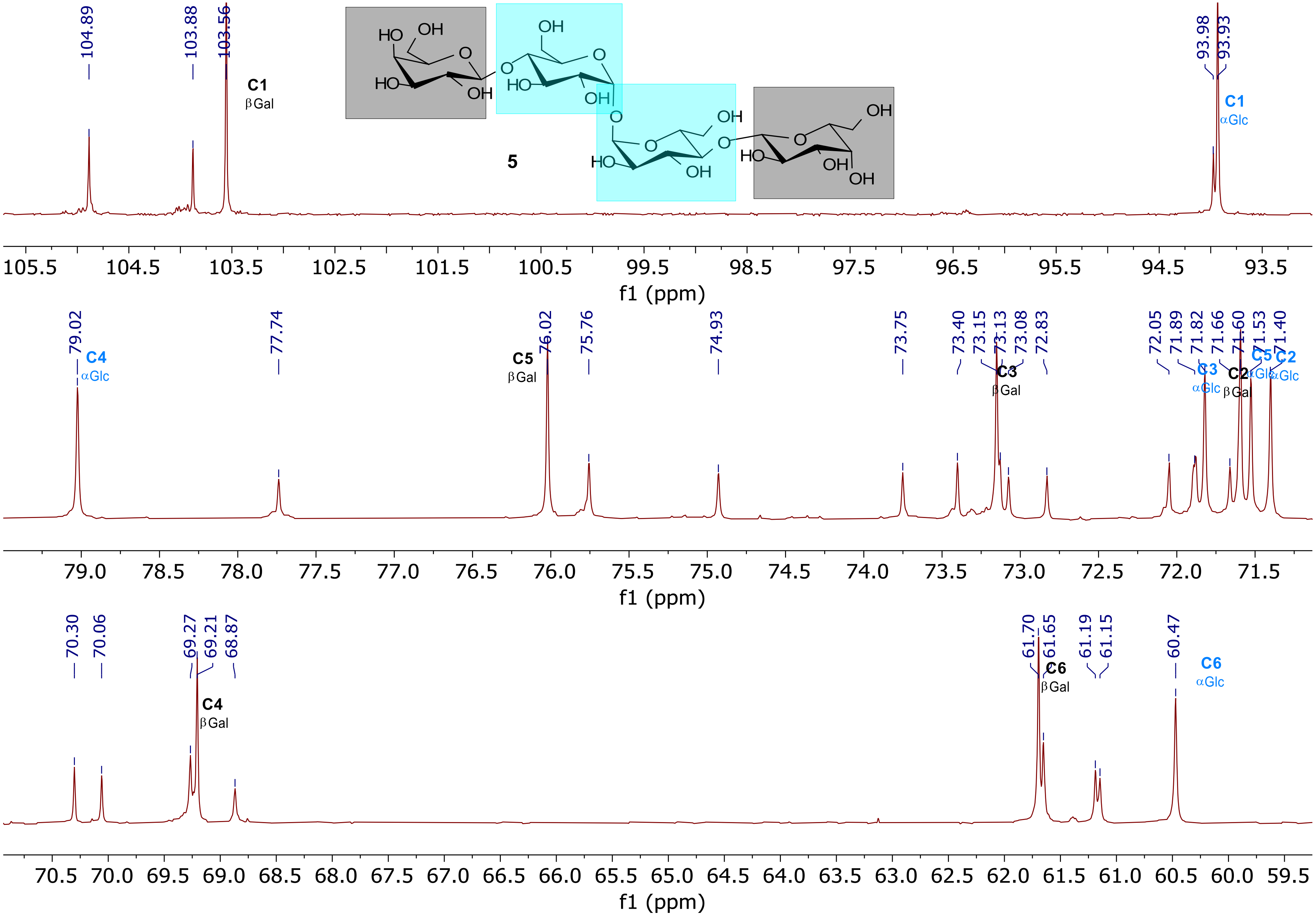

Figure S21. Complete assignment of ^{13}C NMR spectrum of tetrasaccharide **5** derived from trehalose.

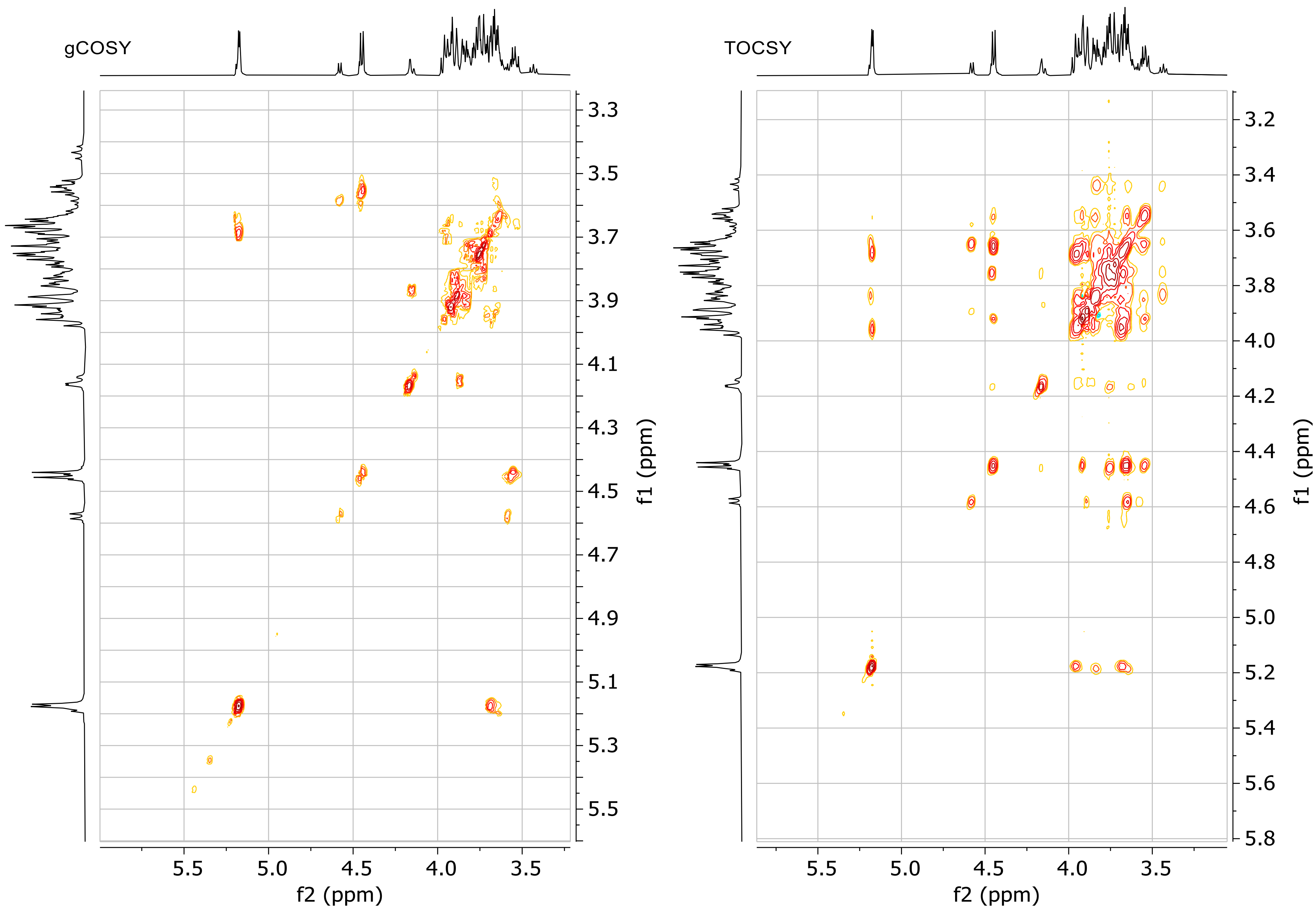

Figure S22. gCOSY and TOCSY (500 MHz, D_2O) for the mixture of tetrasaccharides **4** and **5** derived from trehalose.

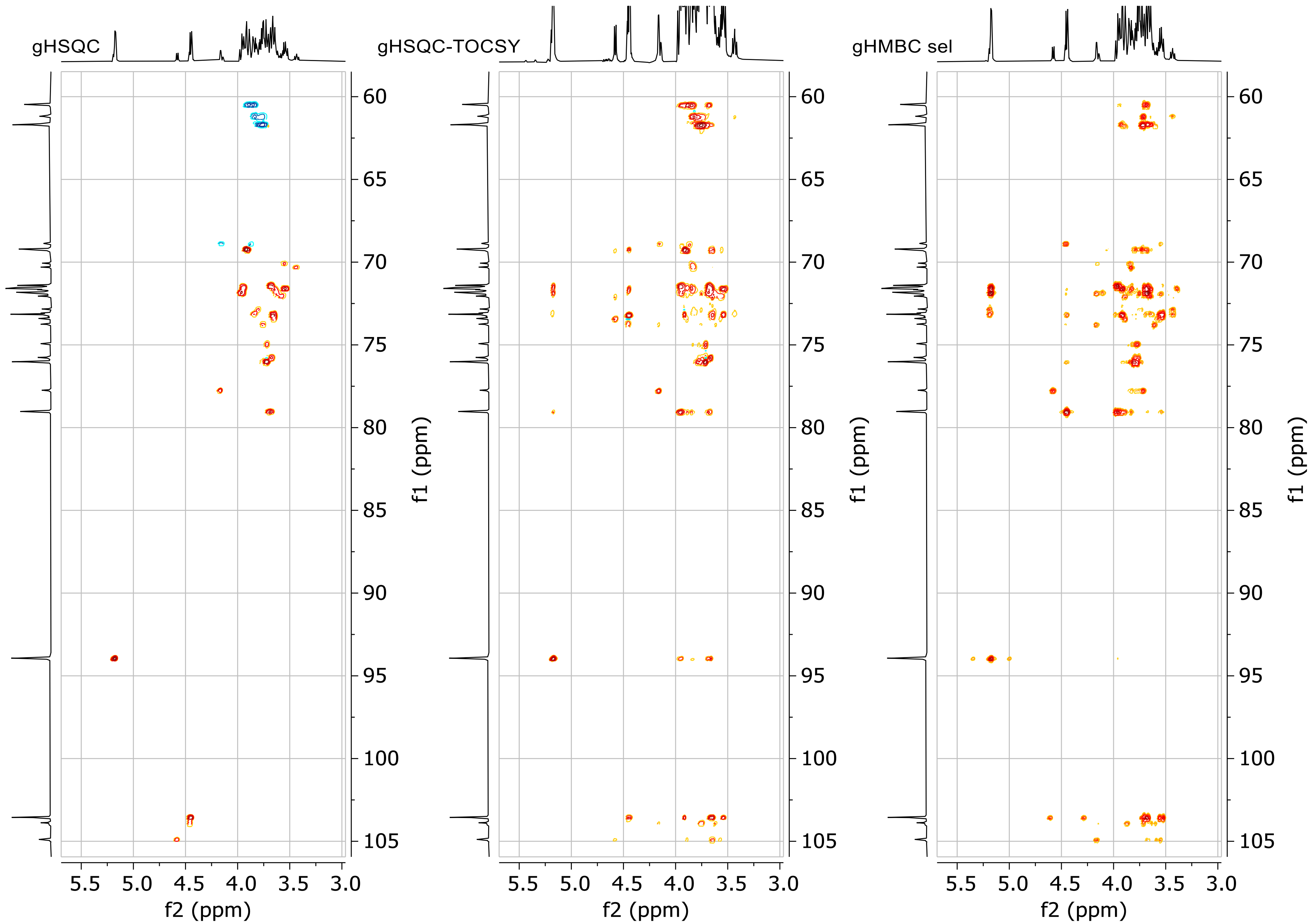

Figure S23. Multiplicity-edited gHSQC (methylene: blue cross peaks; methine: red cross peaks), gHSQC-TOCSY and gHMBC semiselective (500 MHz, D_2O) for the mixture of tetrasaccharides **4** and **5** derived from trehalose.

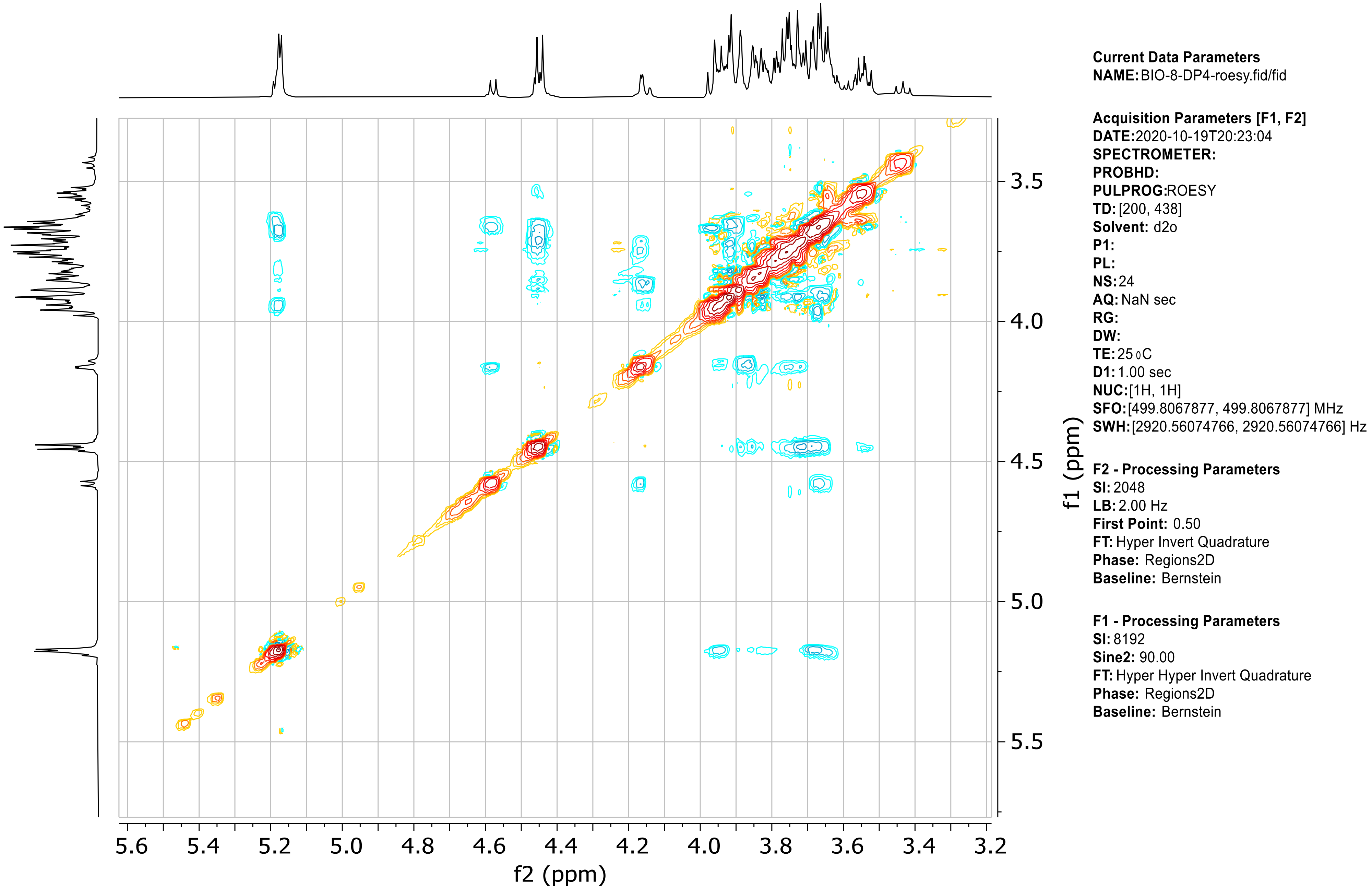

Figure S24. ROESY (500 MHz, D_2O) for the mixture of tetrasaccharides **4** and **5** derived from trehalose.

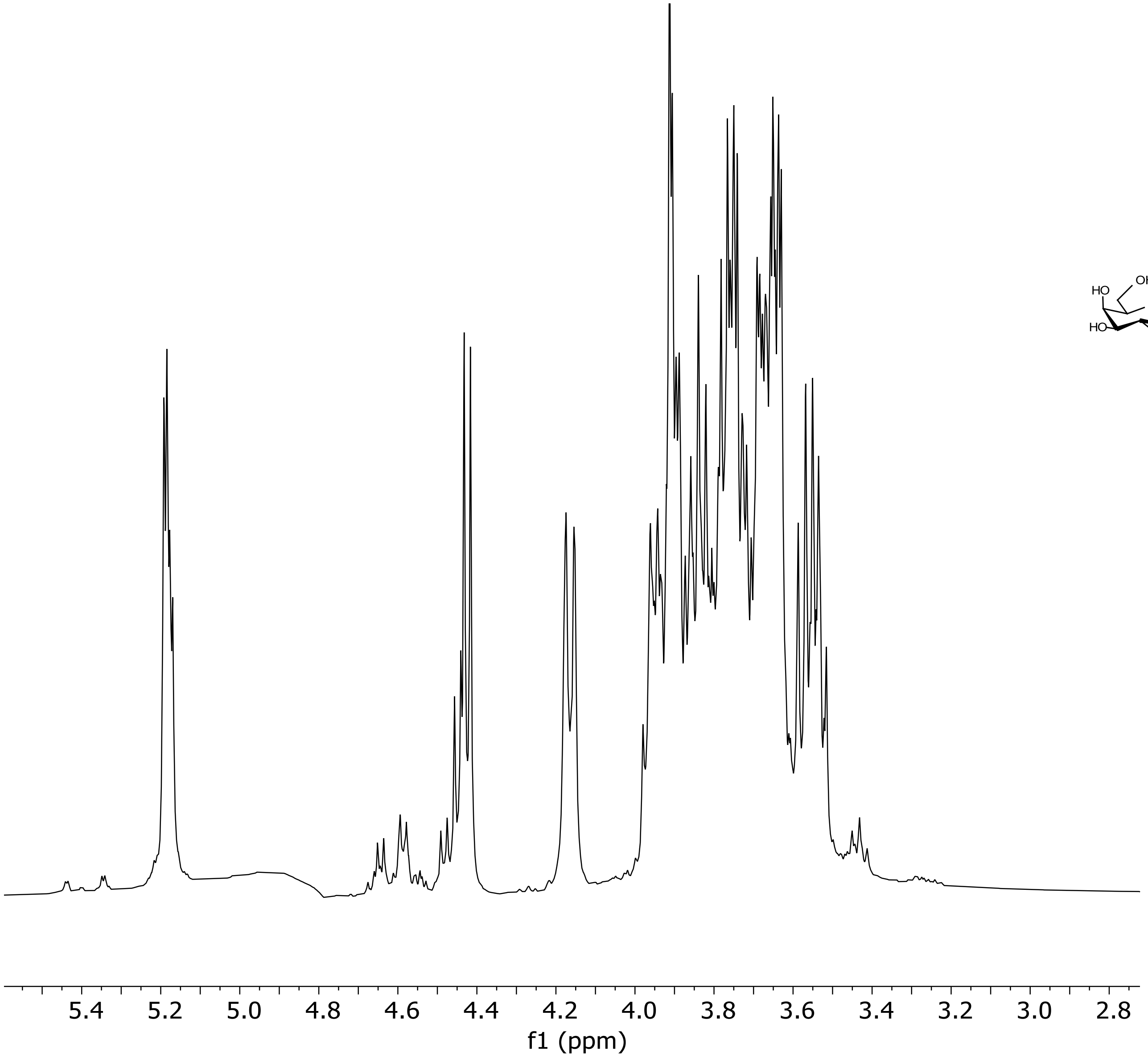

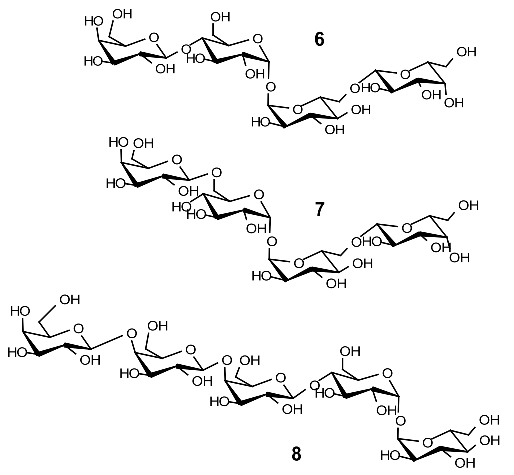

Current Data Parameters
NAME: BIO-9-DP5-1h-presat.fid/fid

Acquisition Parameters
DATE: 2020-10-20T13:44:04
SPECTROMETER:
PROBHD:
PULPROG: PRESAT
TD: 8192
Solvent: d2o
P1:
PL:
NS: 8
AQ: Infinity sec
RG:
DW:
TE: 25 0C
D1: 2.00 sec
NUC: 1H
SFO: 499.8066588 MHz
SWH: 2880.1843318 Hz

F2 - Processing Parameters
SI: 65536
First Point: 0.50
FT: Hyper Invert Quadrature
Phase: Imported

Figure S25. ^{1}H NMR (500 MHz, D_2O) for the mixture of tetrasaccharides **6** and **7**, and pentasaccharide **8** derived from trehalose.

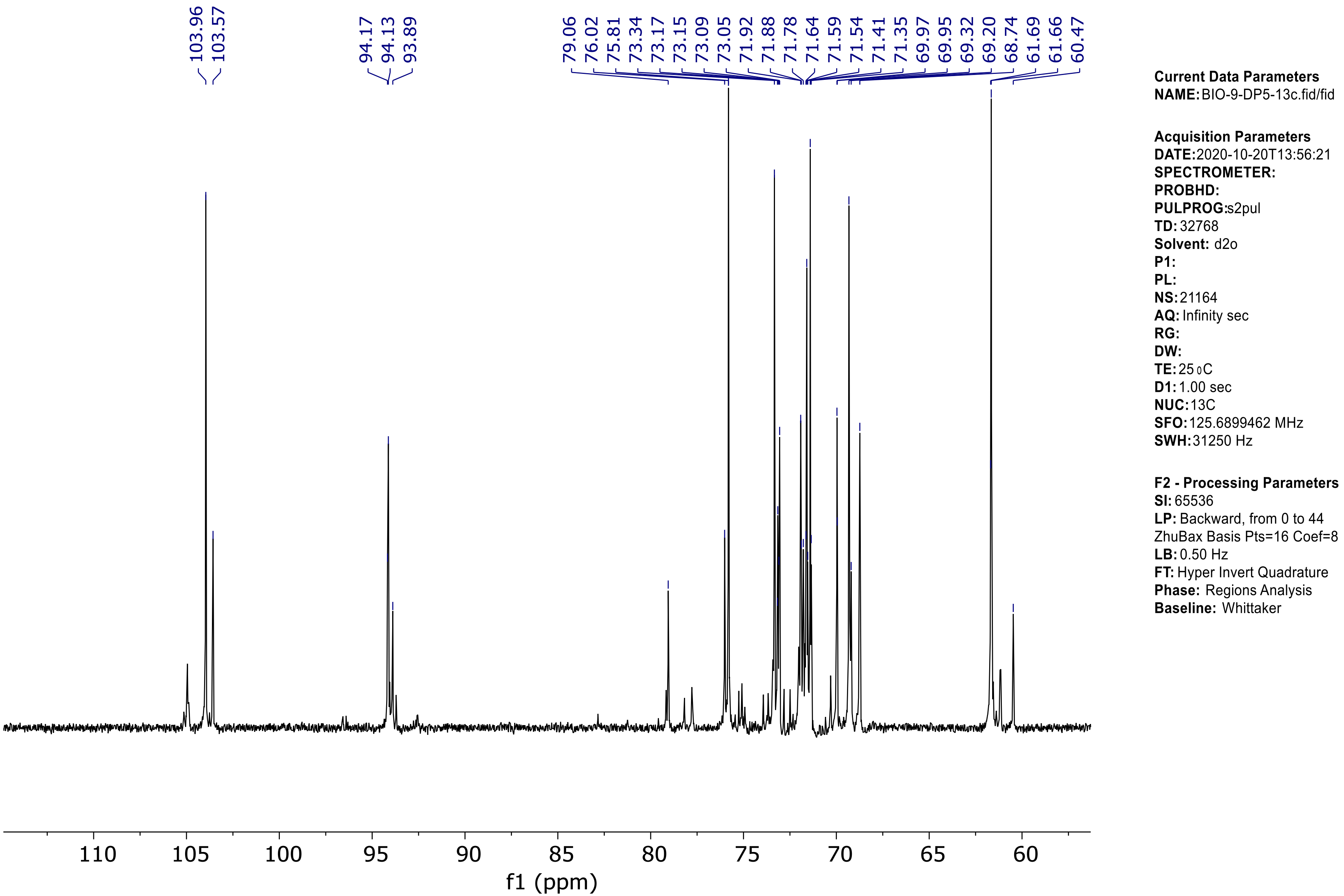

Figure S26. ^{13}C NMR (125 MHz, D_2O) for the mixture of tetrasaccharides **6** and **7**, and pentasaccharide **8** derived from trehalose.

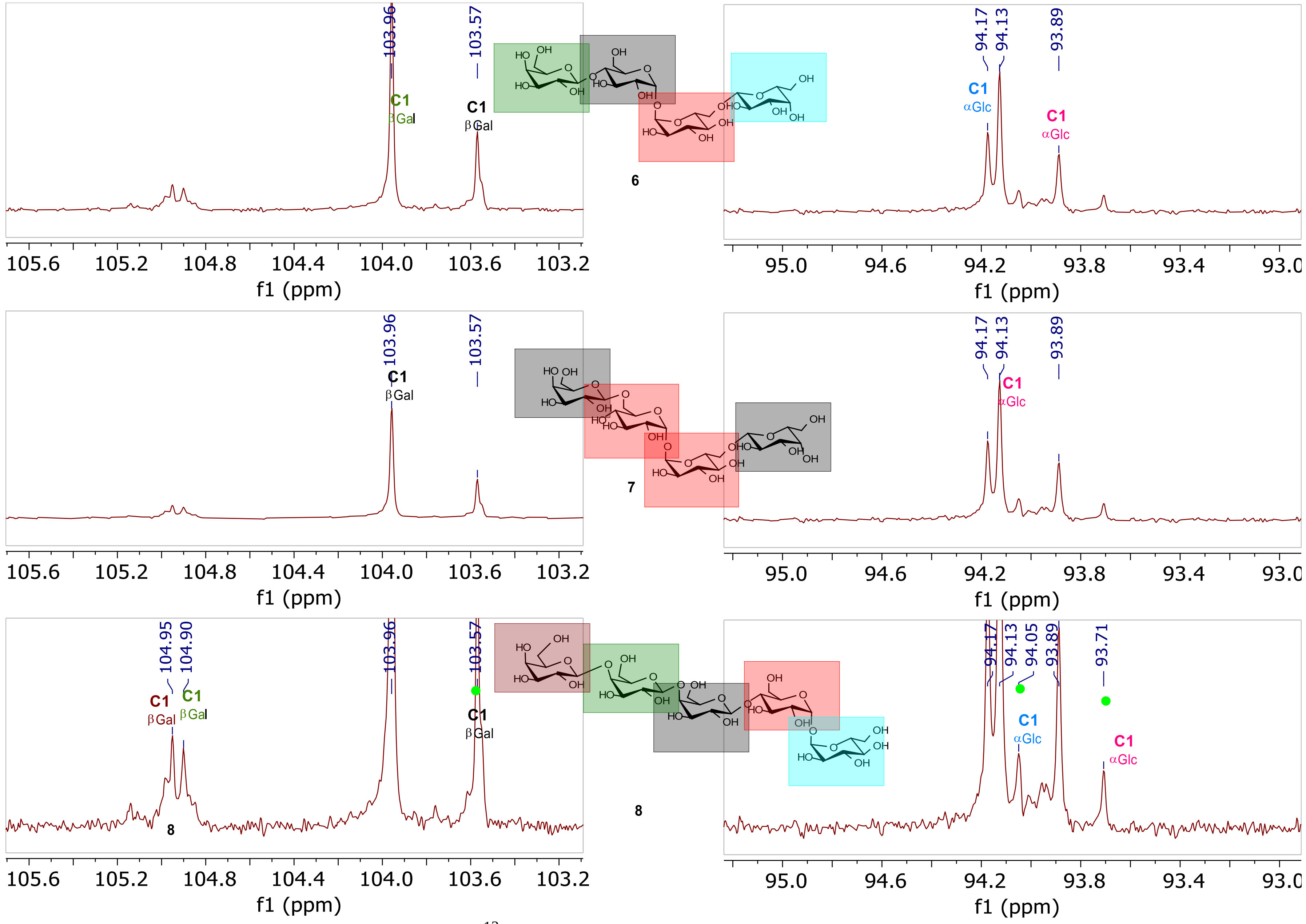

Figure S27. Complete assignment of anomeric region of ^{13}C spectrum NMR of tetrasaccharides **6** and **7**, and pentasaccharide **8** derived from trehalose.

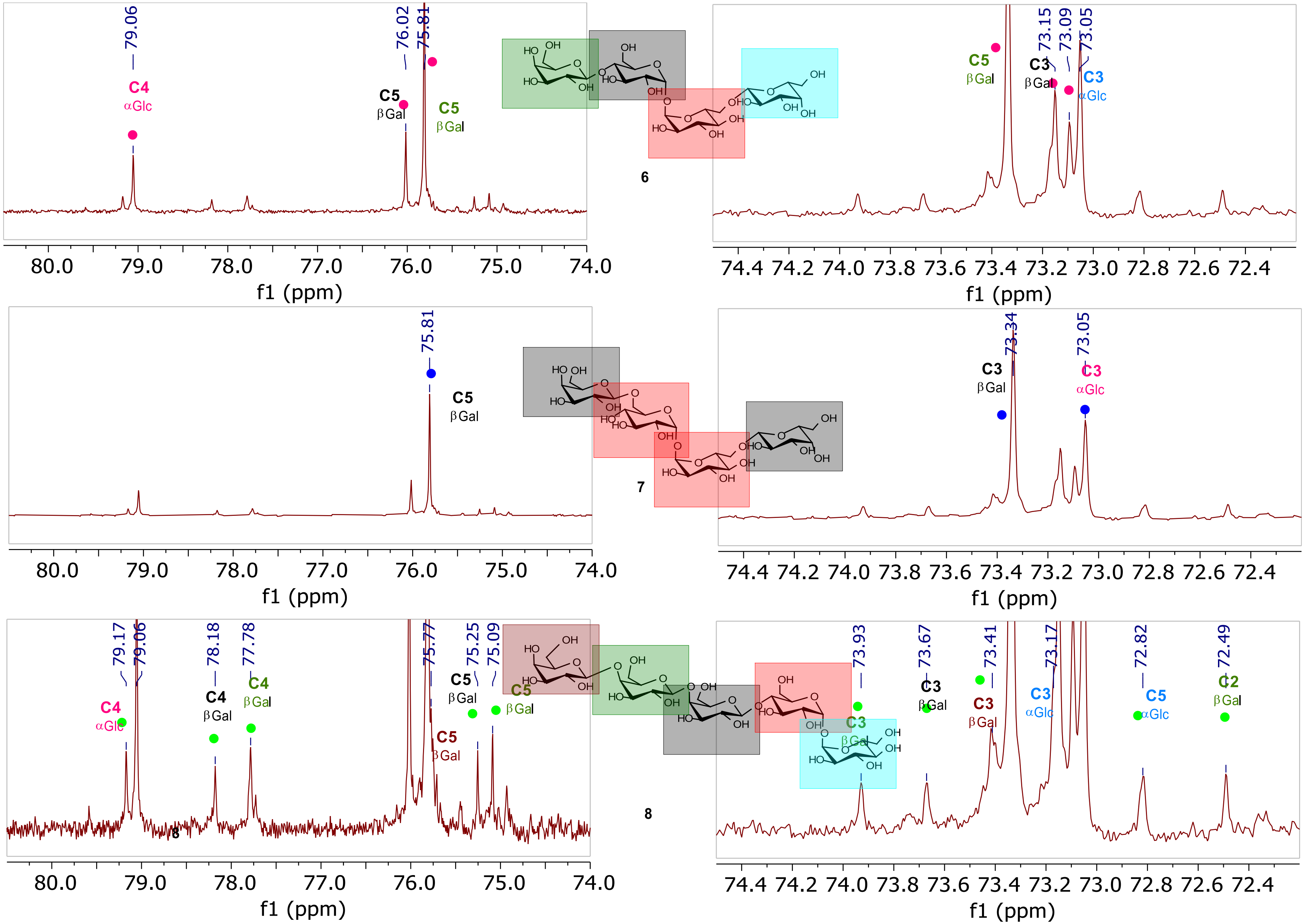

Figure S28. Complete assignment of 80-72 ppm region of ^{13}C spectrum NMR of tetrasaccharides **6** and **7**, and pentasaccharide **8** derived from trehalose.

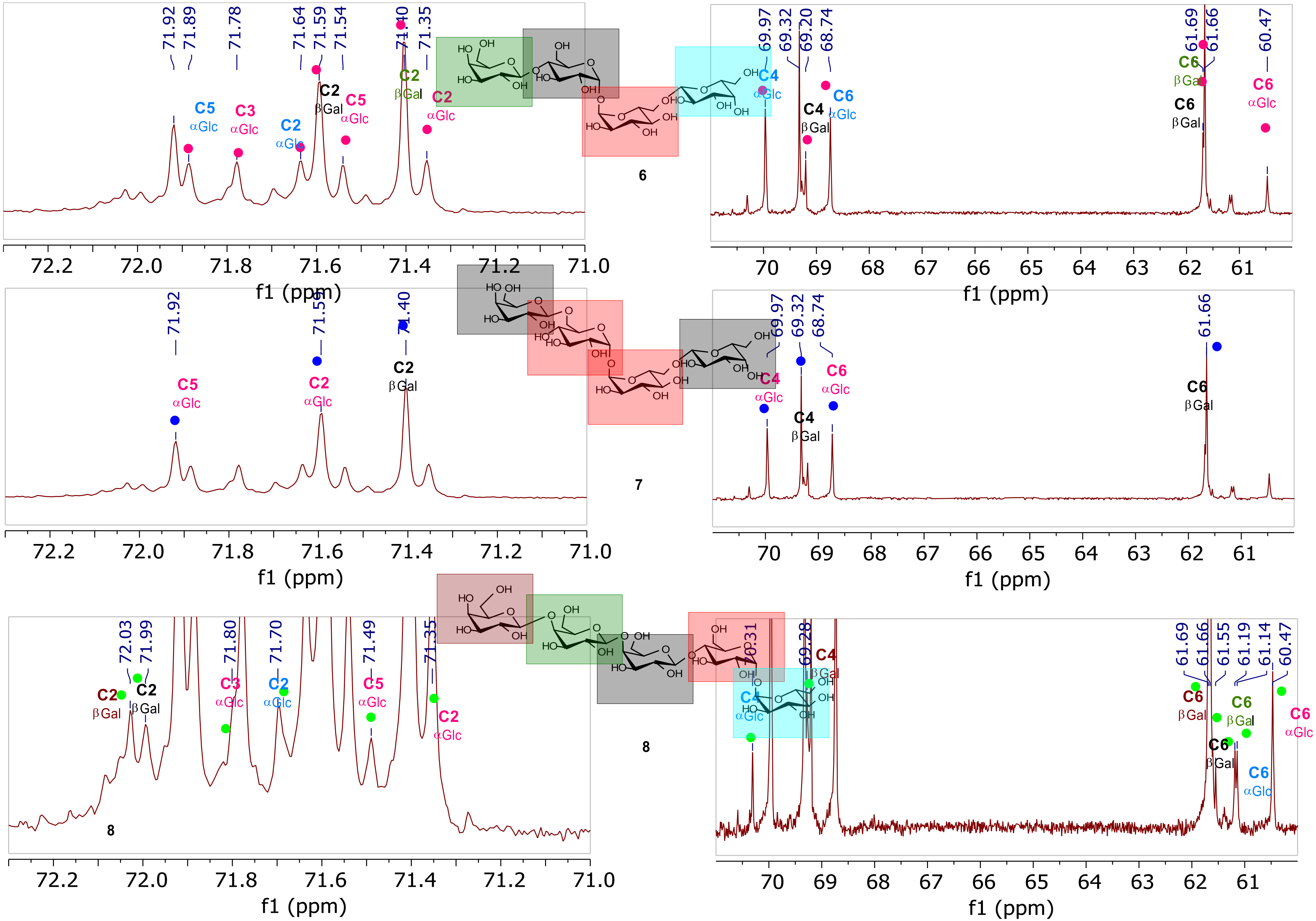

Figure S29. Complete assignment of 72-60 ppm region of ^{13}C spectrum NMR of tetrasaccharides **6** and **7**, and pentasaccharide **8** derived from trehalose.

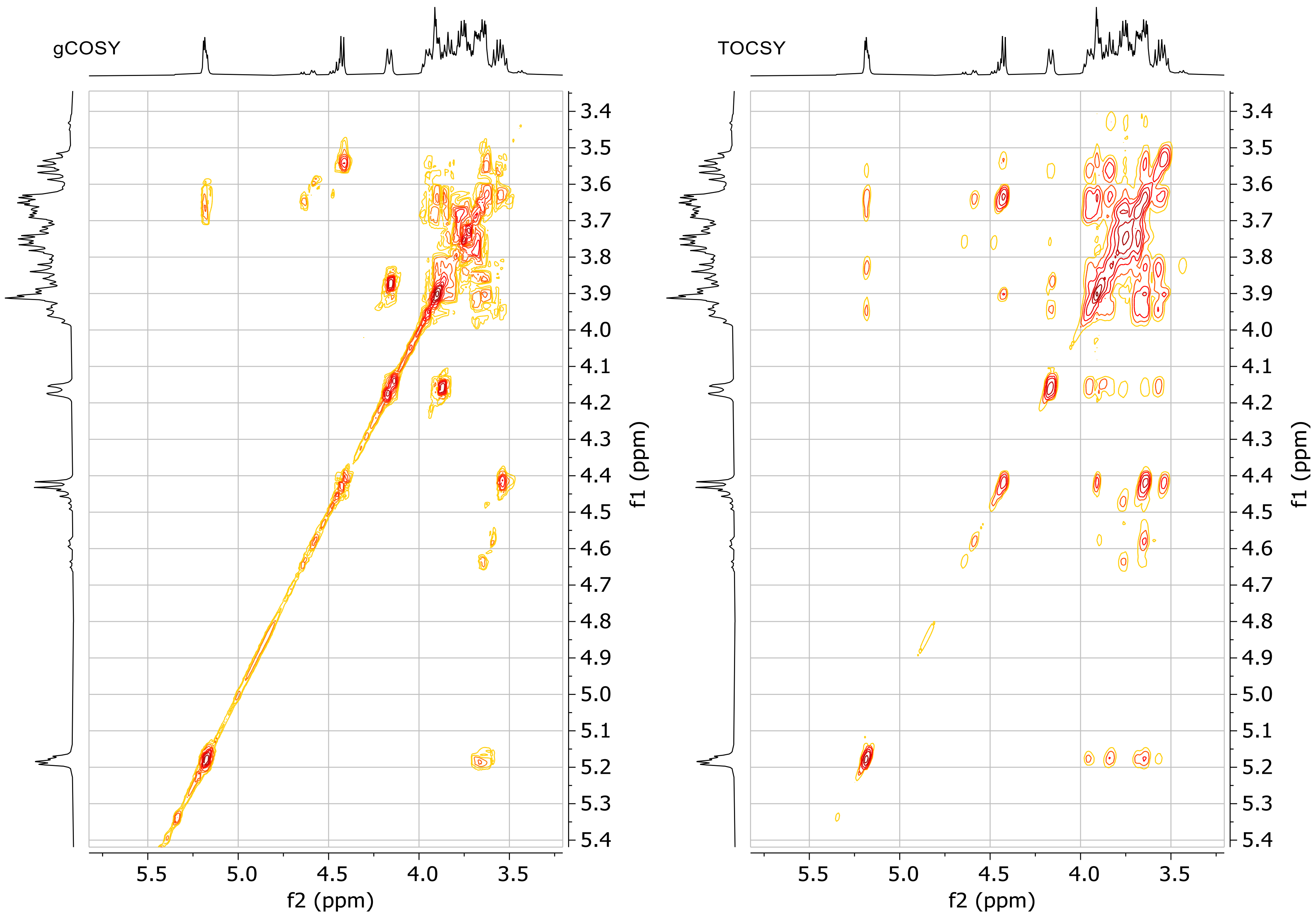

Figure S30. gCOSY and TOCSY (500 MHz, D_2O) for the mixture of tetrasaccharides **6** and **7**, and pentasaccharide **8** derived from trehalose.

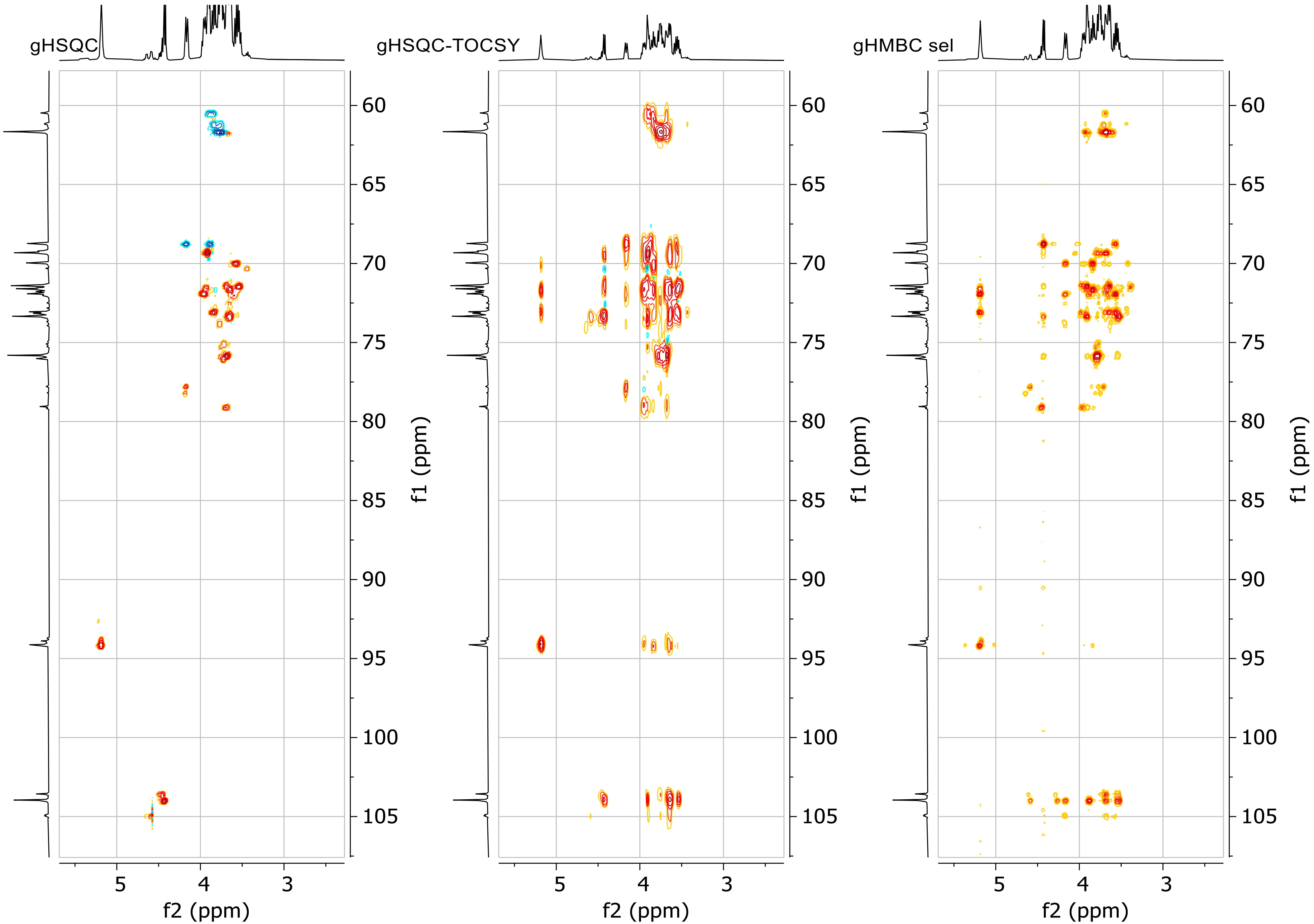

Figure S31. Multiplicity-edited gHSQC (methylene: blue cross peaks; methine: red cross peaks), gHSQC-TOCSY and gHMBC semiselective (500 MHz, D_2O) for the mixture of tetrasaccharides **6** and **7**, and pentasaccharide **8** derived from trehalose.

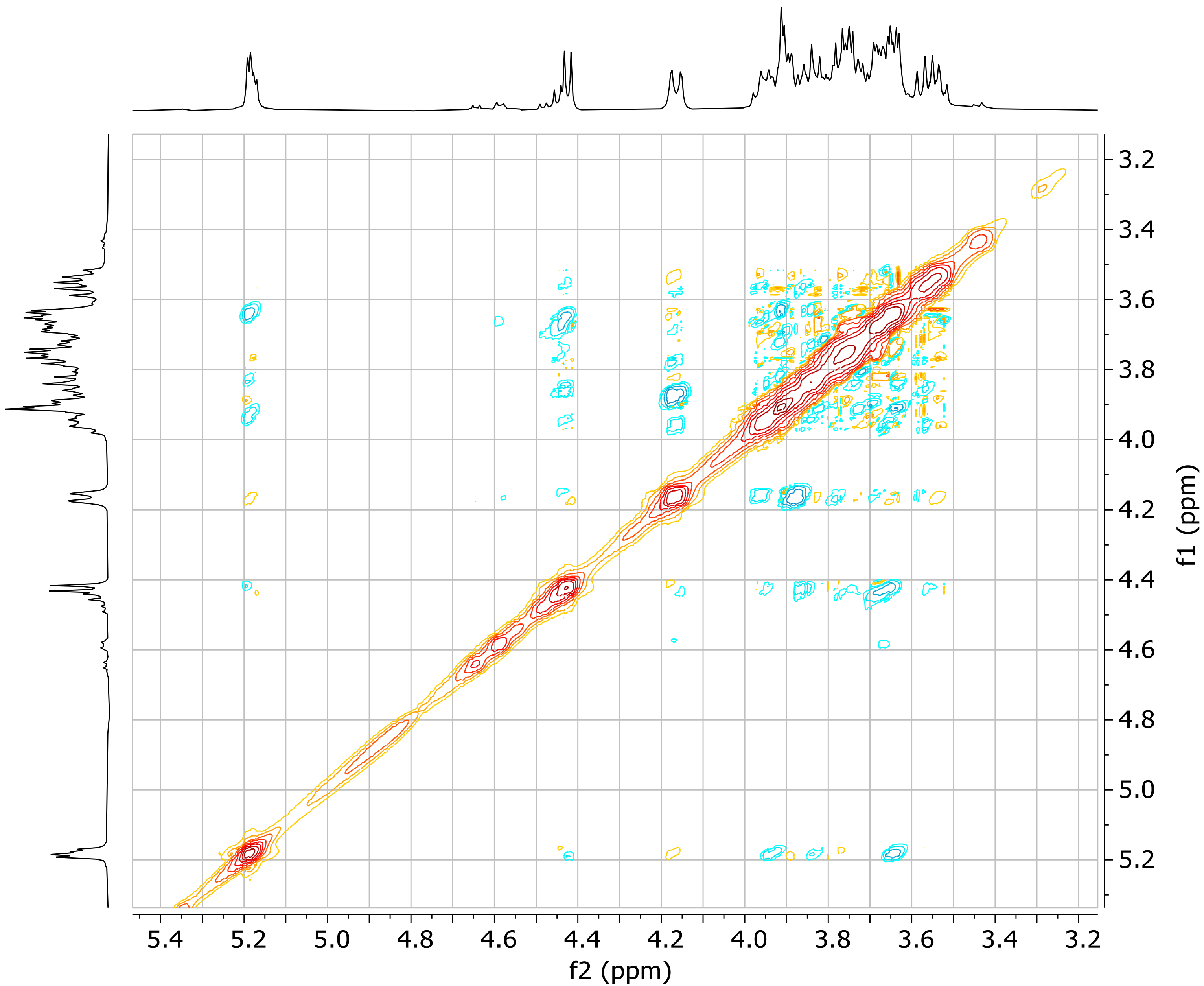

Current Data Parameters
NAME: BIO-9-DP5-roesy.fid/fid

Acquisition Parameters [F1, F2]
DATE: 2020-10-20T21:00:57
SPECTROMETER:
PROBHD:
PULPROG: ROESY
TD: [200, 432]
Solvent: d2o
P1:
PL:
NS: 24
AQ: NaN sec
RG:
DW:
TE: 25 0C
D1: 1.00 sec
NUC: [1H, 1H]
SFO: [499.8066588, 499.8066588] MHz
SWH: [2880.1843318, 2880.1843318] Hz

F2 - Processing Parameters
SI: 2048
LB: 2.00 Hz
First Point: 0.50
FT: Hyper Invert Quadrature
Phase: Regions2D
Baseline: Bernstein

F1 - Processing Parameters
SI: 8192
Sine2: 90.00
FT: Hyper Hyper Invert Quadrature
Phase: Regions2D
Baseline: Bernstein

Figure S32. ROESY (500 MHz, D_2O) for the mixture of of tetrasaccharides **6** and **7**, and pentasaccharide **8** derived from trehalose.

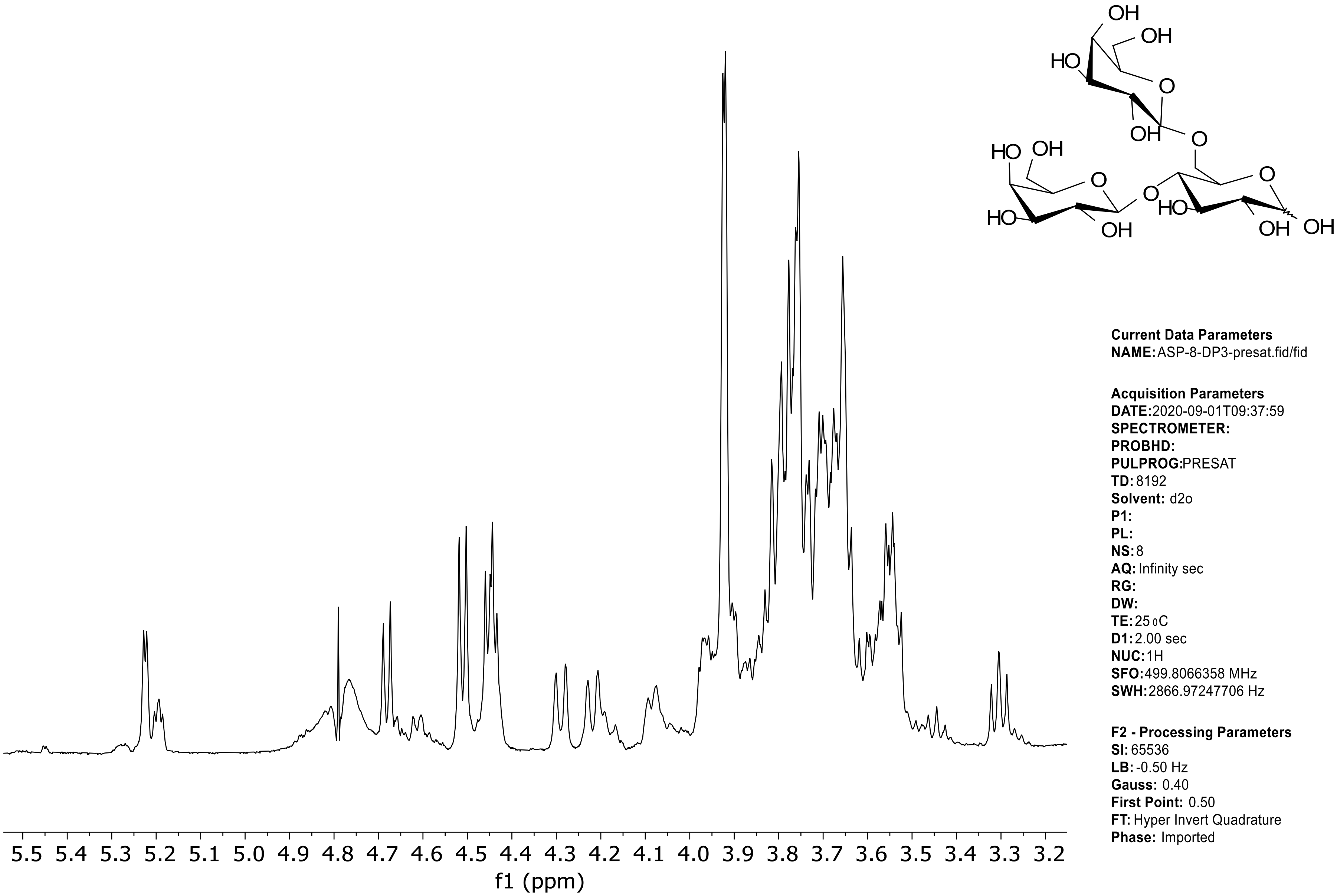

Figure S33. ^{1}H NMR (500 MHz, D_2O) of trisaccharide **9** derived from lactose.

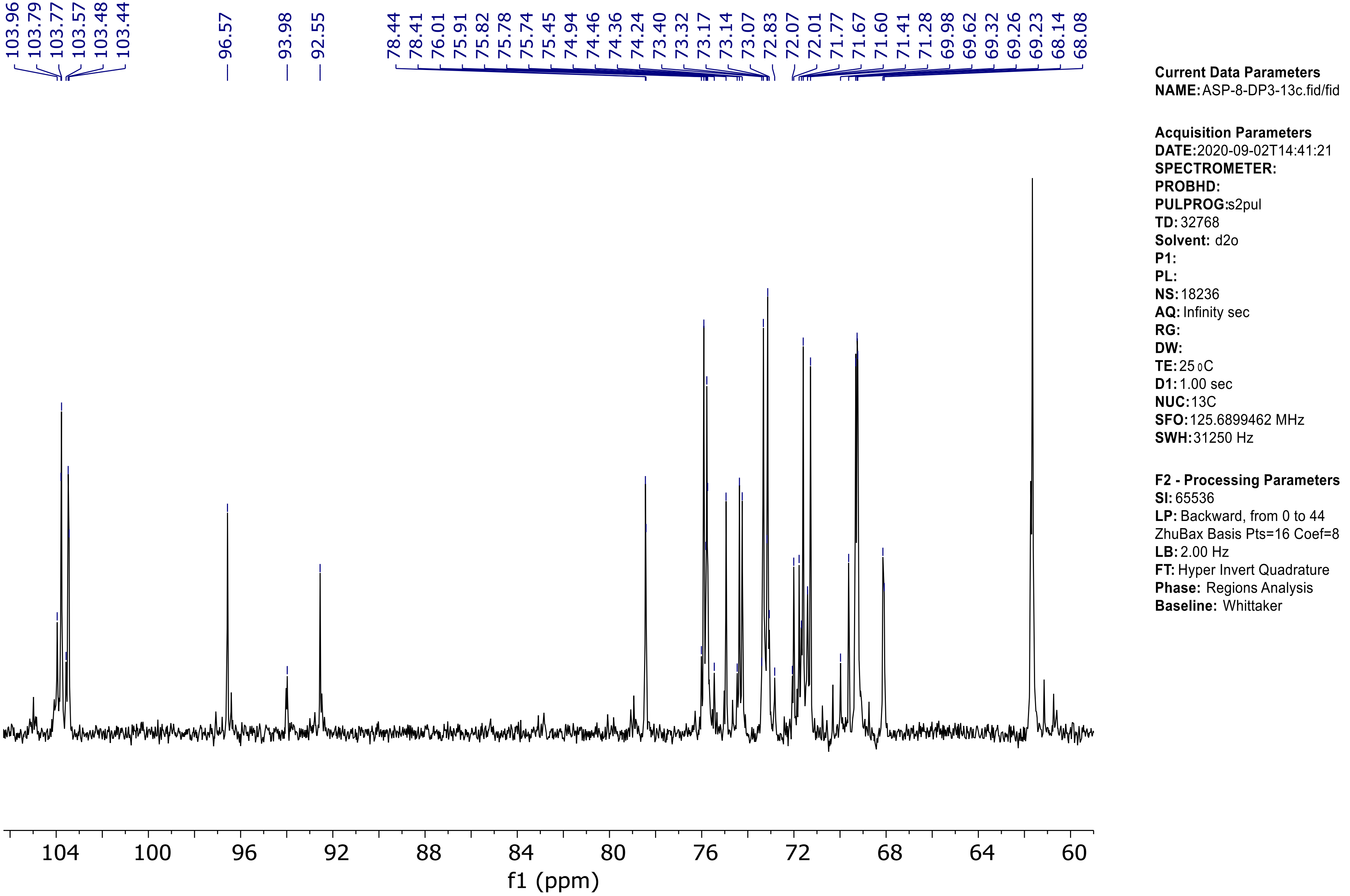

Figure S34. ^{13}C NMR (125 MHz, D_2O) of trisaccharide **9** derived from lactose.

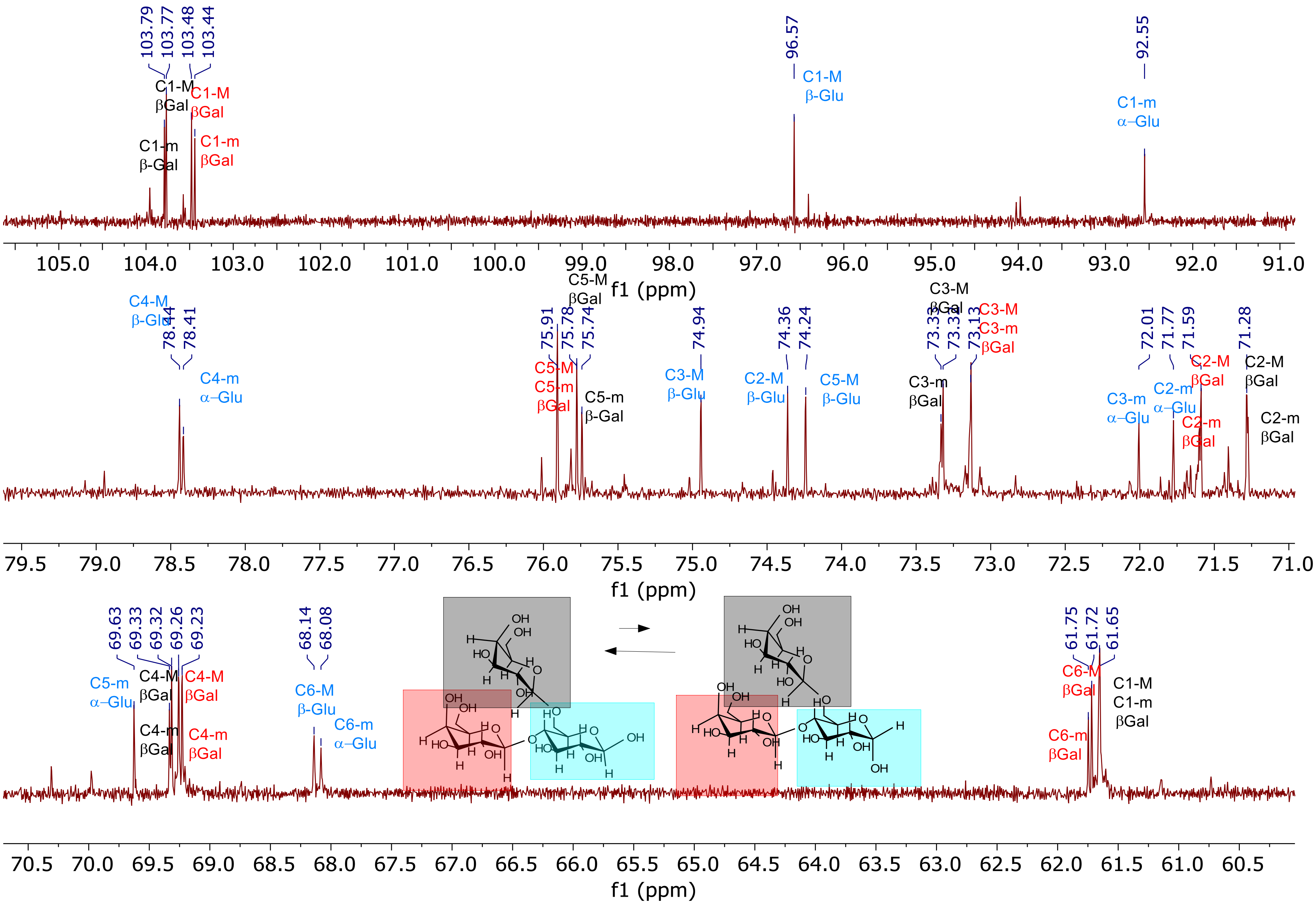

Figure S35. Complete assignment of ^{13}C NMR spectrum of trisaccharide **9** derived from lactose .

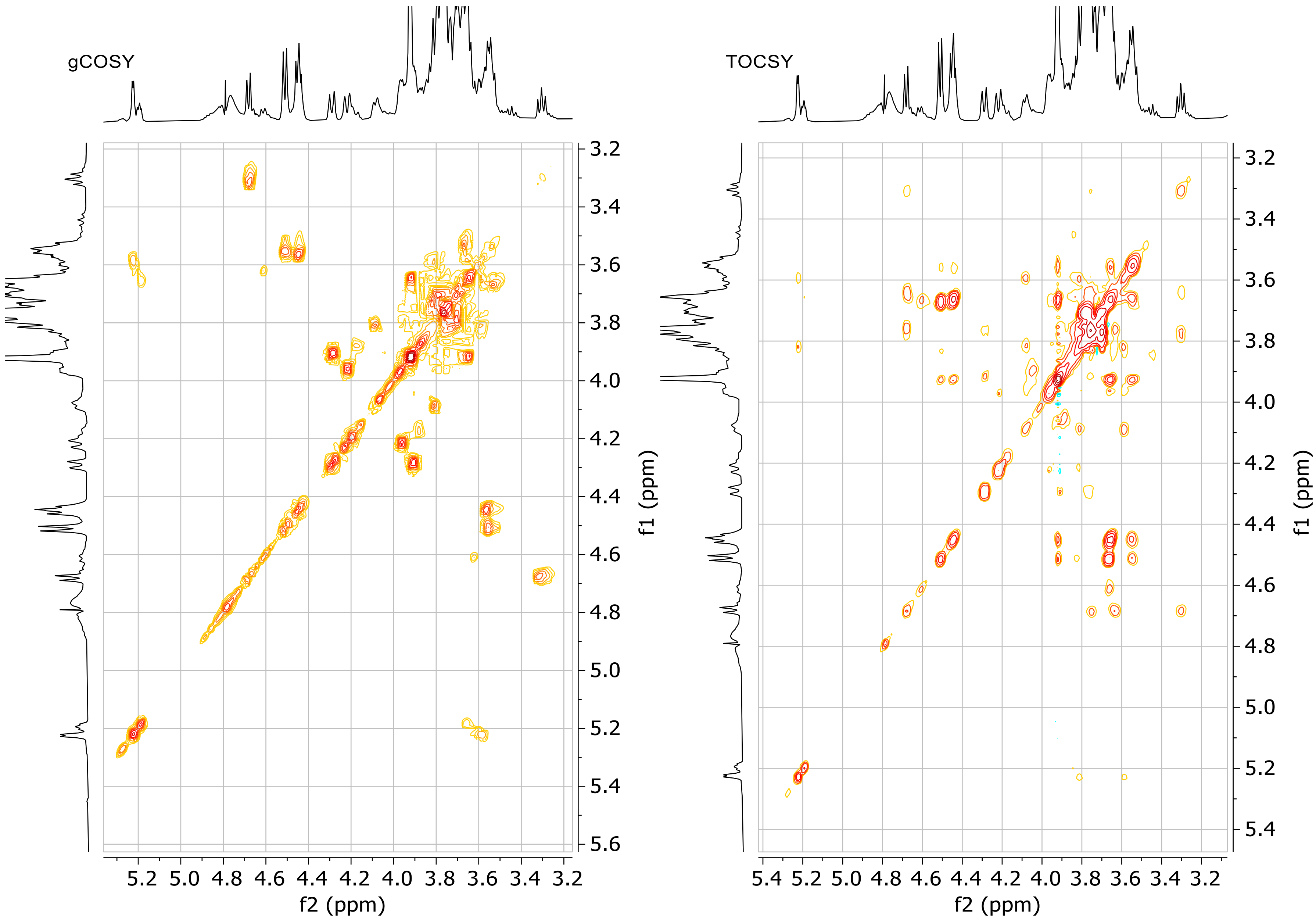

Figure S36. gCOSY and TOCSY (500 MHz, D_2O) of trisaccharide **9** derived from lactose.

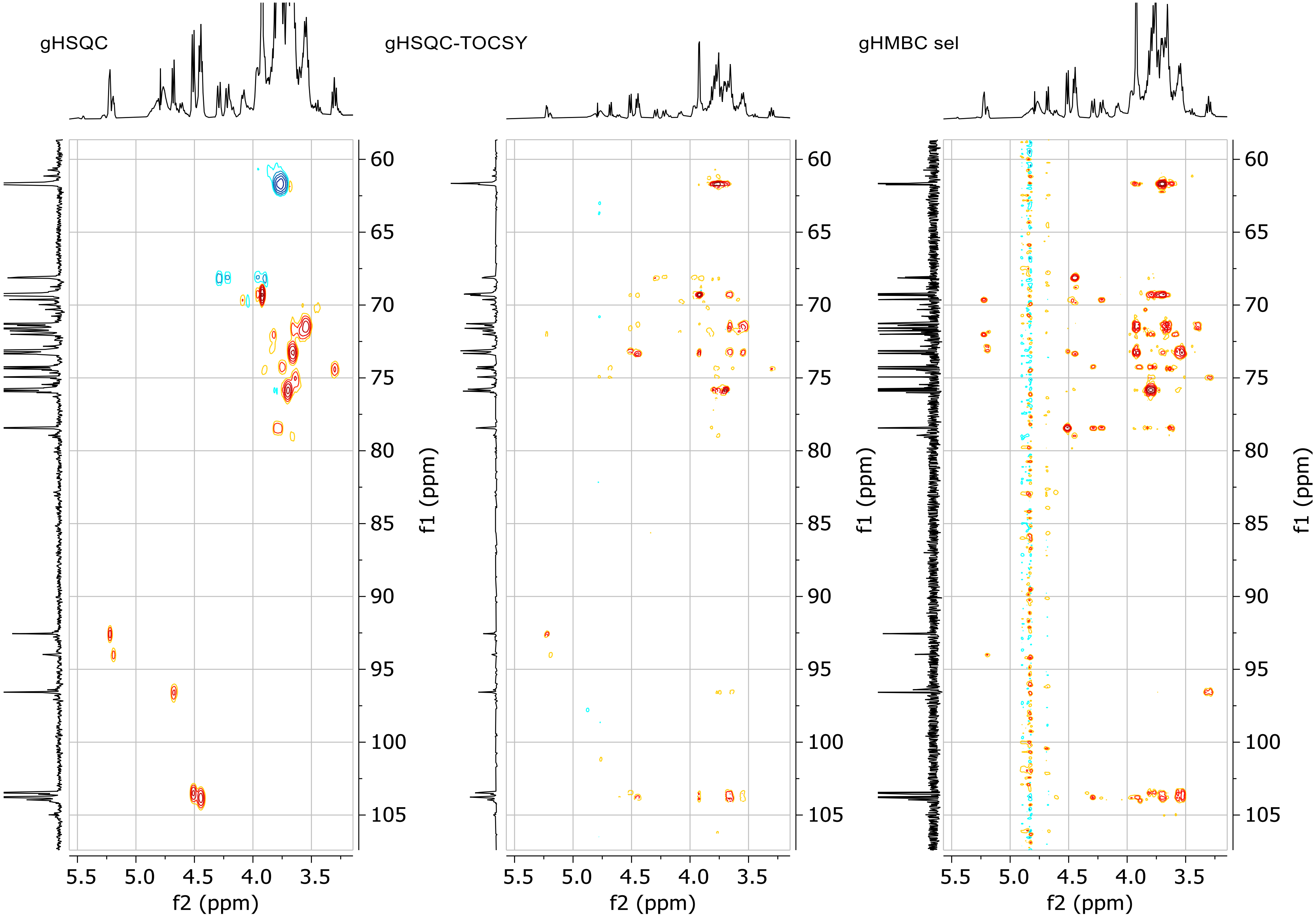

Figure S37. Multiplicity-edited gHSQC (methylene: blue cross peaks; methine: red cross peaks), gHSQC-TOCSY and gHMBC semiselective (500 MHz, D_2O) of trisaccharide **9** derived from lactose.

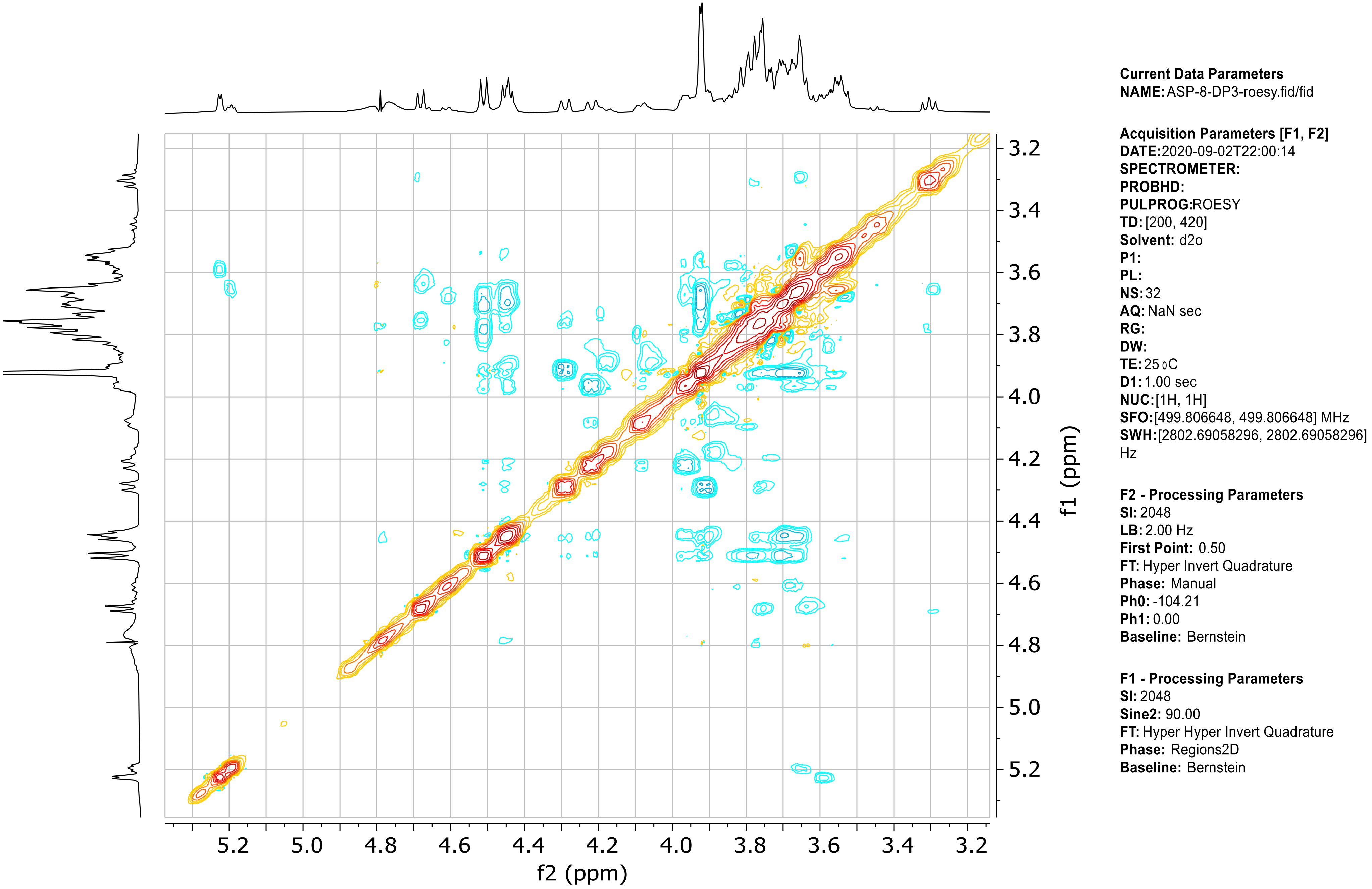

Figure S38. ROESY (500 MHz, D_2O) of trisaccharide **9** derived from lactose.

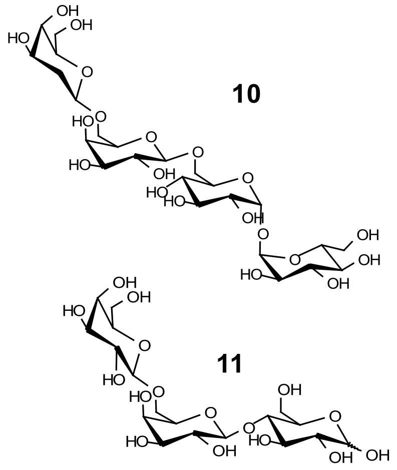

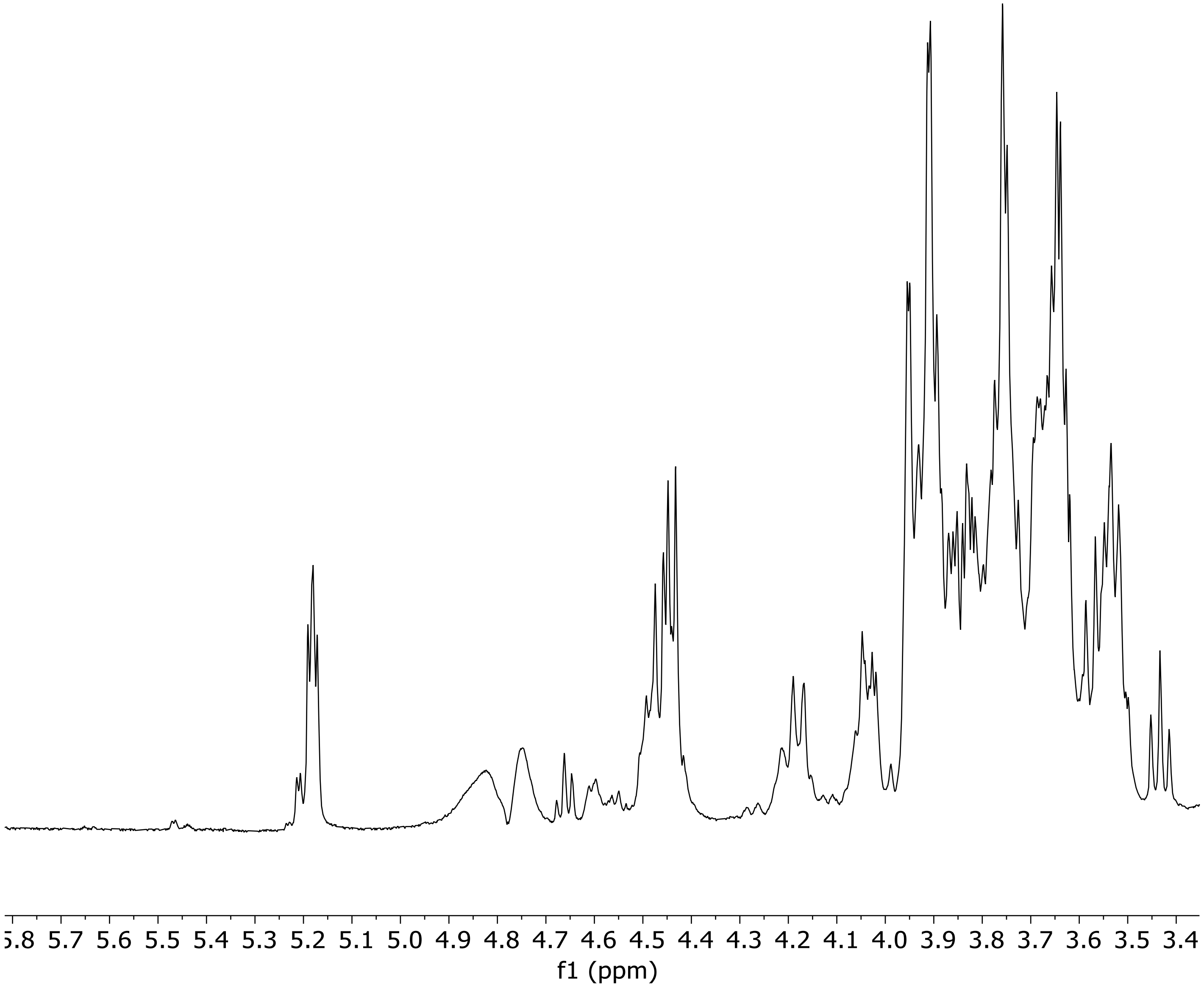

Figure S39. ^{1}H NMR (500 MHz, D_2O)) for the mixture of tetrasaccharide **10** derived from trehalose and trisaccharide **11** derived from lactose.

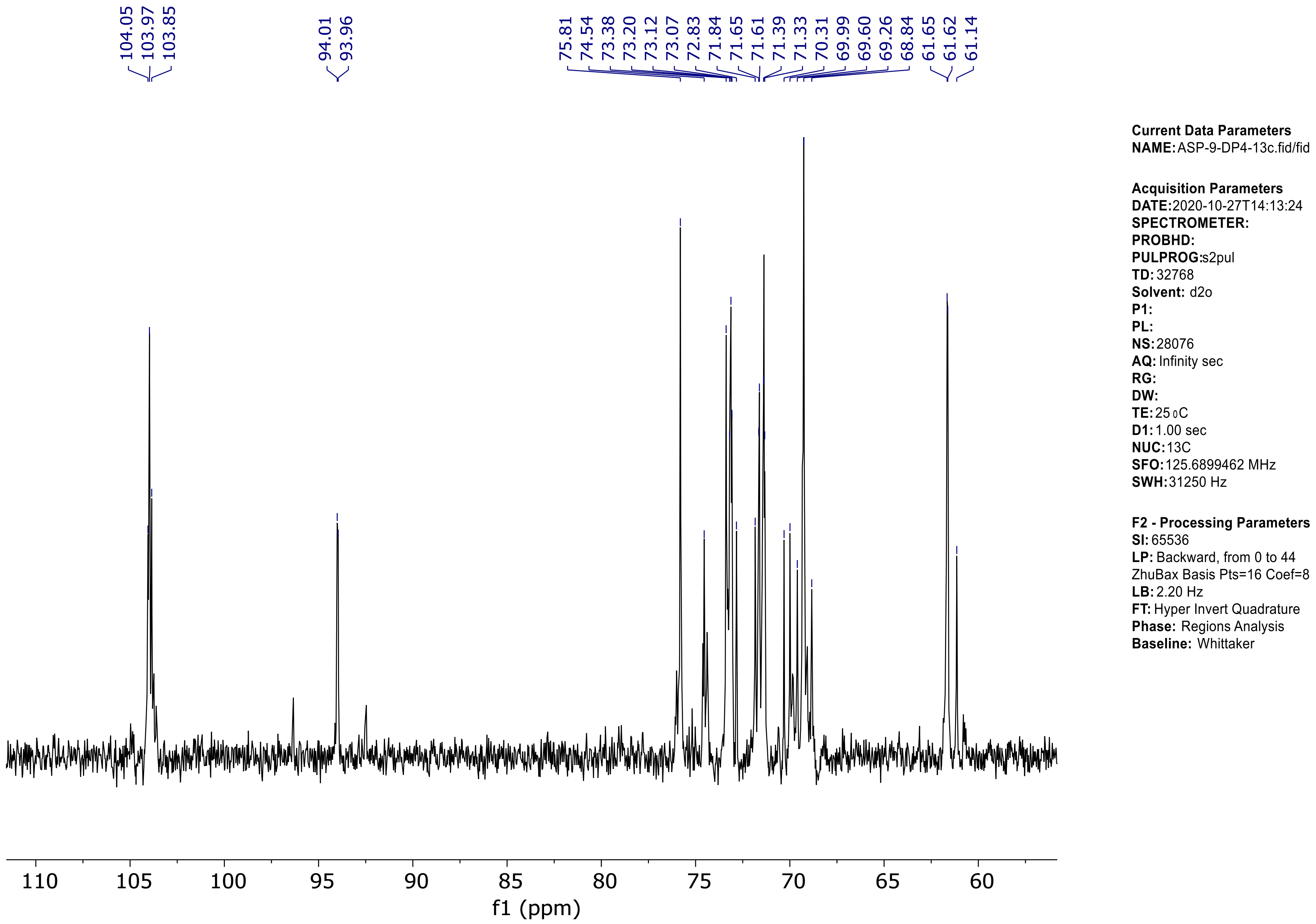

Figure S40. ^{13}C NMR (125 MHz, D_2O) for the mixture of tetrasaccharide **10** derived from trehalose and trisaccharide **11** derived from lactose.

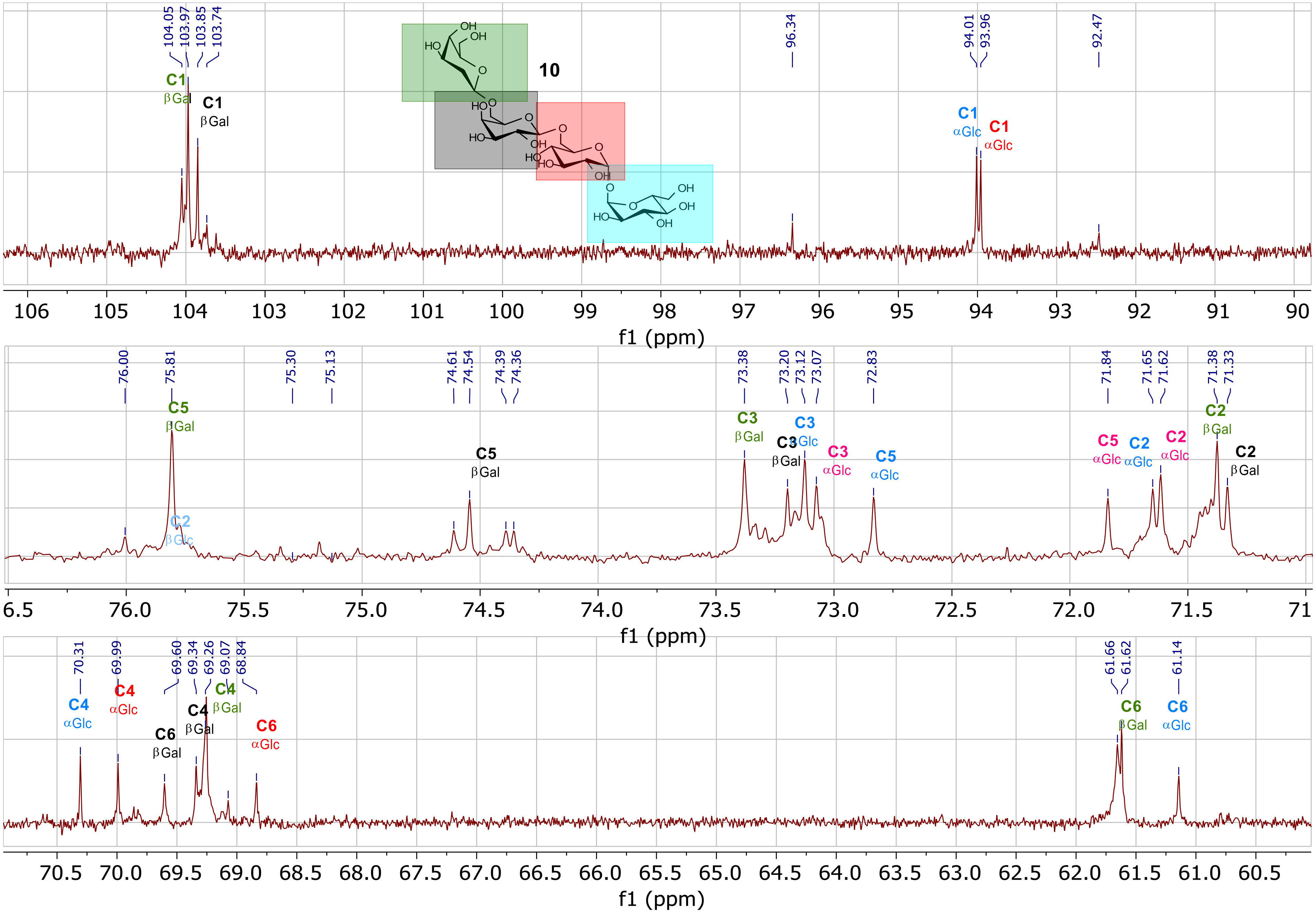

Figure S41. Complete assignment of ^{13}C NMR spectrum of tetrasaccharide **10** derived from trehalose.

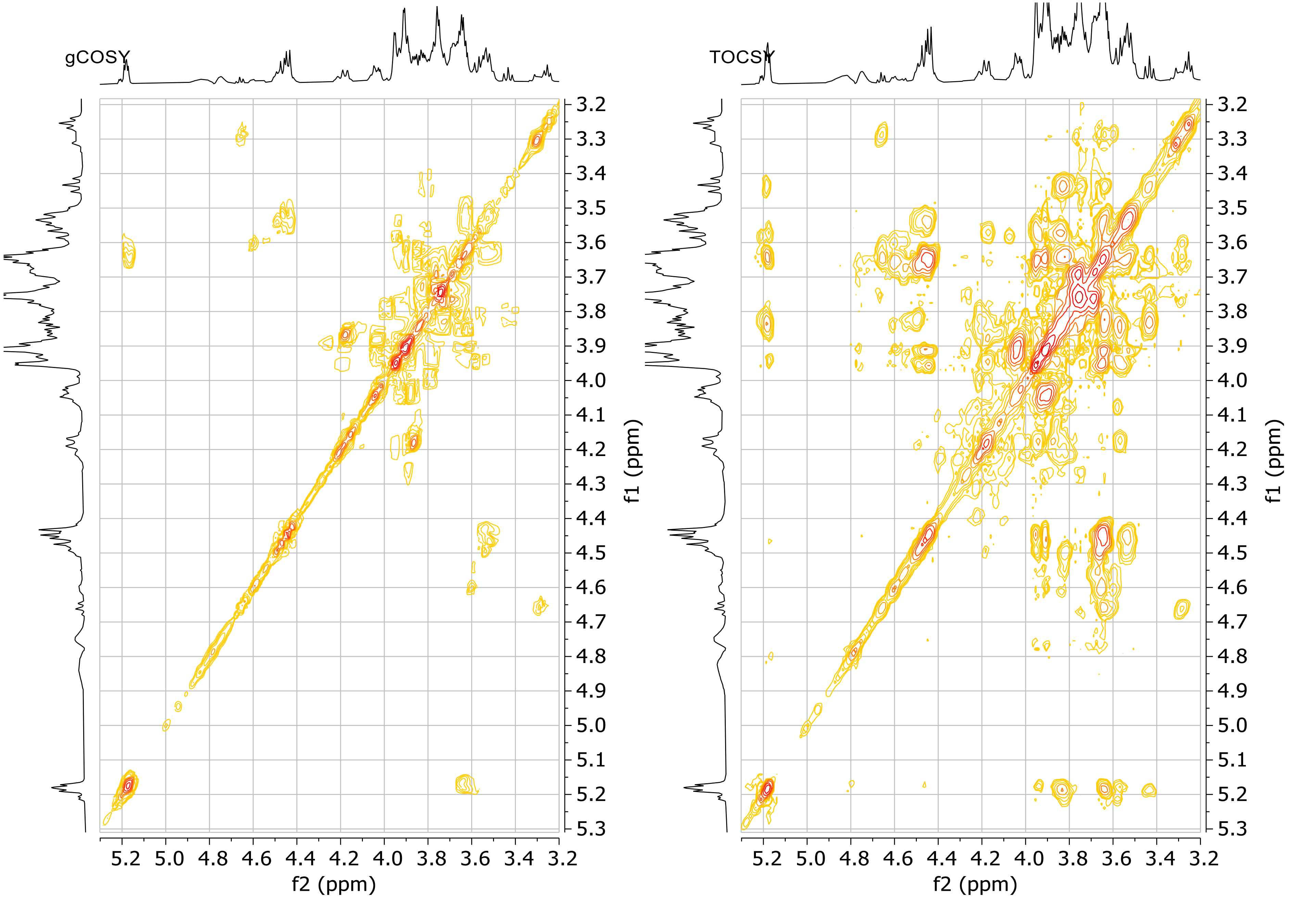

Figure S42. gCOSY and TOCSY (500 MHz, D_2O) for the mixture of tetrasaccharide **10** derived from trehalose. and trisaccharide **11** derived from lactose.

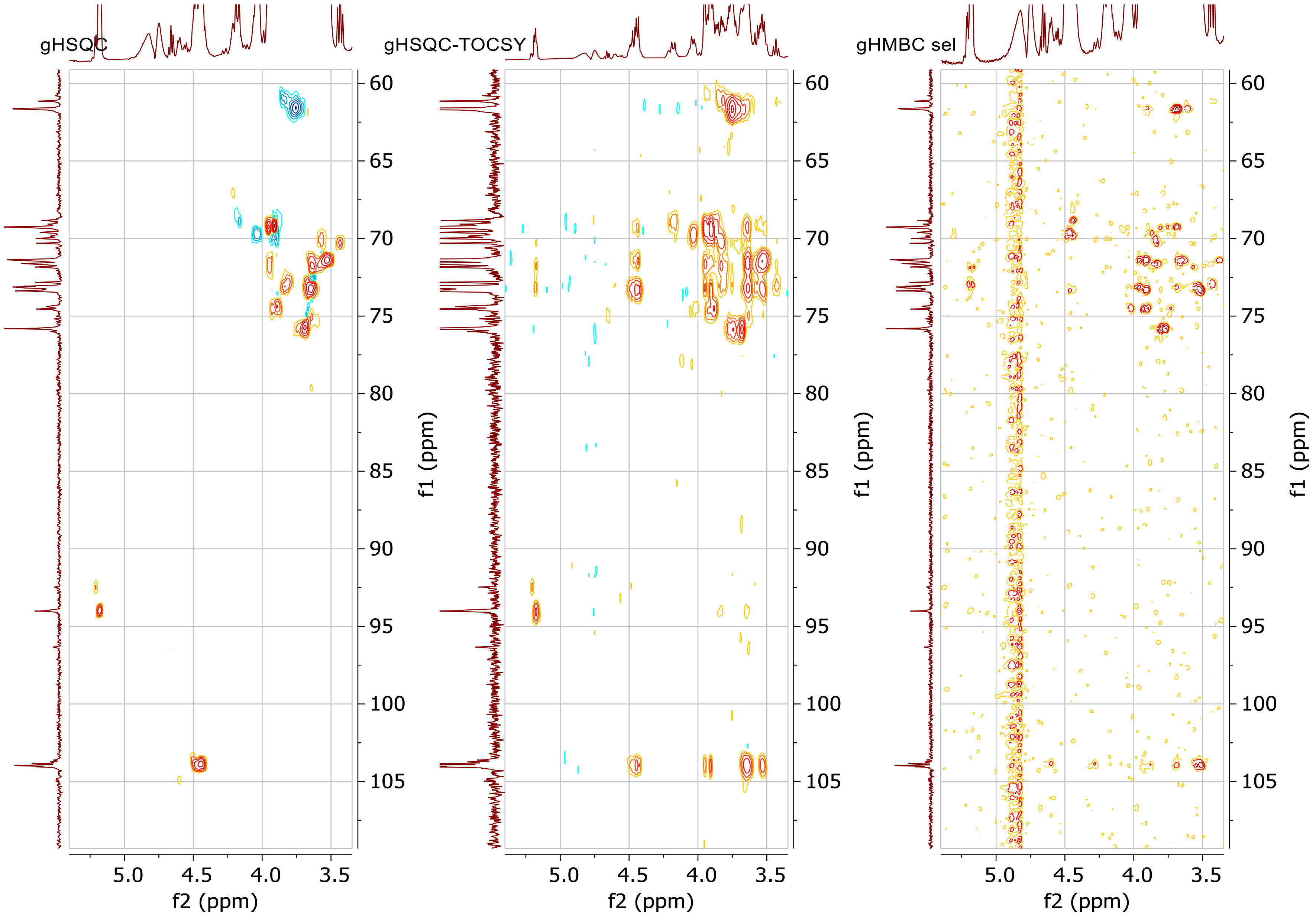

Figure S43. Multiplicity-edited gHSQC (methylene: blue cross peaks; methine: red cross peaks), gHSQC-TOCSY and gHMBC semiselective (500 MHz, D_2O) for the mixture of tetrasaccharide **10** derived from trehalose. and trisaccharide **11** derived from lactose.
